# Polycyclopropanated Lipid-Inspired Ionic Liquids as High Energy-Density Fuel Candidates

**DOI:** 10.1021/acssuschemeng.5c13132

**Published:** 2026-02-23

**Authors:** Christopher M. Butch, Richard A. O’Brien, Raychell A. Jerdo, James H. Davis, Matthias Zeller, Brooks D. Rabideau, Patrick C. Hillesheim, Arsalan Mirjafari

**Affiliations:** † Department of Chemistry, 14828 State University of New York at Oswego, Oswego, New York 13126, United States; ‡ Department of Chemistry, 5557 University of South Alabama, Mobile, Alabama 36688, United States; § Department of Chemistry, 8522 Purdue University, West Lafayette, Indiana 47907, United States; ∥ Department of Chemical & Biomolecular Engineering, University of South Alabama, Mobile, Alabama 36688, United States; ⊥ Department of Chemistry, 6049 Illinois State University, Normal, Illinois 61761, United States

**Keywords:** sustainable aviation fuels, lipid-inspired ionic liquids, high-energy-density fuels, CuAAC click chemistry, polycyclopropanated compounds, molecular engineering, bioinspired design

## Abstract

Climate change necessitates the urgent development of sustainable alternatives to petroleum-derived fuels for energy-intensive applications such as aviation, rocketry, and long-haul transport, where electrification remains impractical. This study presents polycyclopropanated lipid-inspired ionic liquids (PCP-ILs) as the first class of high-energy-density fuel candidates synthesized from renewable bioderived fatty esters. Our design strategy draws inspiration from natural lipid structures, leveraging their inherent fluidity characteristics to create functional ILs. We developed a facile synthesis route using Cu-catalyzed azide–alkyne cycloaddition (CuAAC) click chemistry, which enables direct incorporation of a cyclopropyl ring onto the nitrogen-rich 1,2,3-triazolium headgroup in quantitative yields. The resulting PCP-ILs demonstrate remarkable properties essential for fuel applications, such as negligible vapor pressure, eliminating detectable boiling points, and expected high flash points that enhance safety and storage stability. Strategic placement of cyclopropyl moieties in both the cationic headgroup and aliphatic side chains, mimicking fluidity-conferring features in biological lipids, significantly reduces melting points compared to non-cyclopropanated analogues. Computational and X-ray crystallography studies systematically elucidate how molecular packing and structural organization enable significant melting/freezing point reduction. These PCP-ILs achieve theoretical volumetric energy densities of ca. 30 MJ/L, competitive with conventional aviation fuels, while providing superior safety profiles through negligible vapor pressure and enhanced thermal and chemical stability compared to unsaturated hydrocarbon alternatives. These combined properties demonstrate that bioinspired PCP-ILs can deliver the high technical performance required for demanding energy applications while maintaining sustainability advantages, establishing a pathway for renewable alternatives in difficult-to-decarbonize sectors.

## Graphical abstract

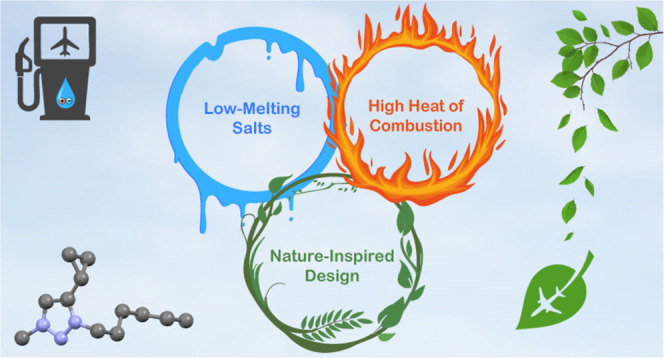

## 1 Introduction

The urgent need to combat climate change requires a transition away from petroleum-based hydrocarbon fuels. Advances in large-scale biofuel production and lithium-ion battery technology have reduced fossil fuel dependence and greenhouse gas emissions in light-duty transportation. However, replacing petroleum-derived hydrocarbon fuels in energy-intensive sectors such as long-haul transport, maritime shipping, aviation, and rocketry remains challenging.^1–3^ Aviation alone contributes 4% of the global temperature increase since the preindustrial era,^4^ exemplifying the disproportionate climate impact of this sector. These applications demand high energy density and energy-to-weight ratios that only petroleum-derived fuels currently provide, creating a persistent fossil fuel dependence.

The aerospace and maritime industries depend heavily on petroleum distillates (C_7_—C_18_) enriched with cyclic and branched alkanes, where ring strain from nontetrahedral bond angles increases the fuel’s net heat of combustion.^2,5^ This dependence on petroleum-based fuels contributes significantly to global emissions, with maritime shipping and aviation together generating 4–5% of global greenhouse gas emissions—nearly two billion tons of CO_2_ annually.^6,7^ While light-duty transportation is gradually transitioning to cleaner technologies, these difficult-to-decarbonize industries are projected to represent an increasing share of global emissions in the coming decades. Additionally, the rapidly growing space sector, including private space tourism, remains largely unregulated despite its expanding environmental footprint that increasingly conflicts with global climate policies.^8^ Therefore, these mounting environmental challenges highlight the urgent need for sustainable, high-energy-density fuel (HEDF) alternatives that can meet the requirements of energy-intensive applications (Figure 1).

**Figure 1:**
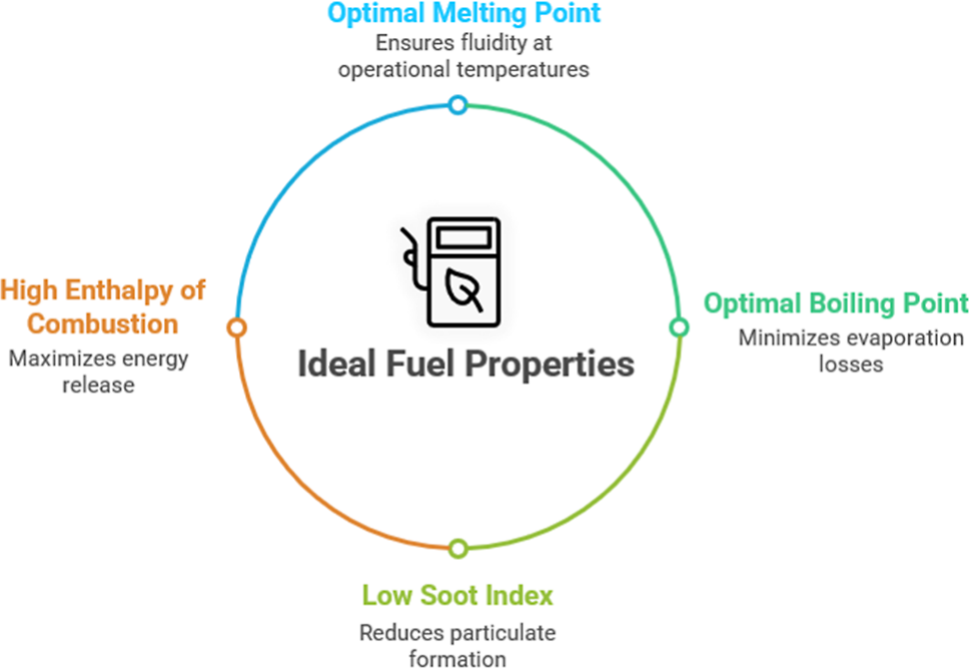
Critical technical design parameters for HEDFs.

Among potential solutions, polycyclopropanated (PCP) compounds represent a promising class of high-energy molecules that could meet the technical requirements of HEDFs (Figure 1) while enabling sustainable production pathways (Figure 2).^9–24^ The cyclopropane ring exhibits notable strains and heat of combustion due to its strained 60° bond angles, storing ∼27 kcal mol^–1^ more energy than its linear counterparts.^24^ This property was demonstrated by Syntin,^25^ a Soviet-era synthetic fuel used in Soyuz and Proton rockets that delivered superior performance compared to kerosene-based fuels. Despite their promising energy properties, PCP synthesis is challenging due to complex, expensive procedures that require hazardous petroleum-derived intermediates. Nevertheless, incorporating multiple cyclopropane rings into hydrocarbon fuels yields high-energy-density compounds. Recently, Cruz-Morales et al. reported that the polycyclopropanated fatty acid methyl esters (fuelimycins, Figure 2) achieve a volumetric enthalpy of combustion of 40.60 MJ/L through theoretical calculations (DFT), exceeding aerospace fuels such as JP-10 (39.6 MJ/L) due to their high density.^26^ However, this biosynthetic pathway produces incompletely cyclopropanated products with residual olefinic motifs,^26^ while optimal fuel performance requires fully saturated derivatives for maximum energy density and thermo-oxidative stability. Very recently, Chowdhury et al. reported a novel class of alkylamine-based PCPs, achieving volumetric energy densities of 38–44 MJ/L that rival or exceed benchmark HEDFs like JP-10 through simple synthetic methods.^24^

**Figure 2:**
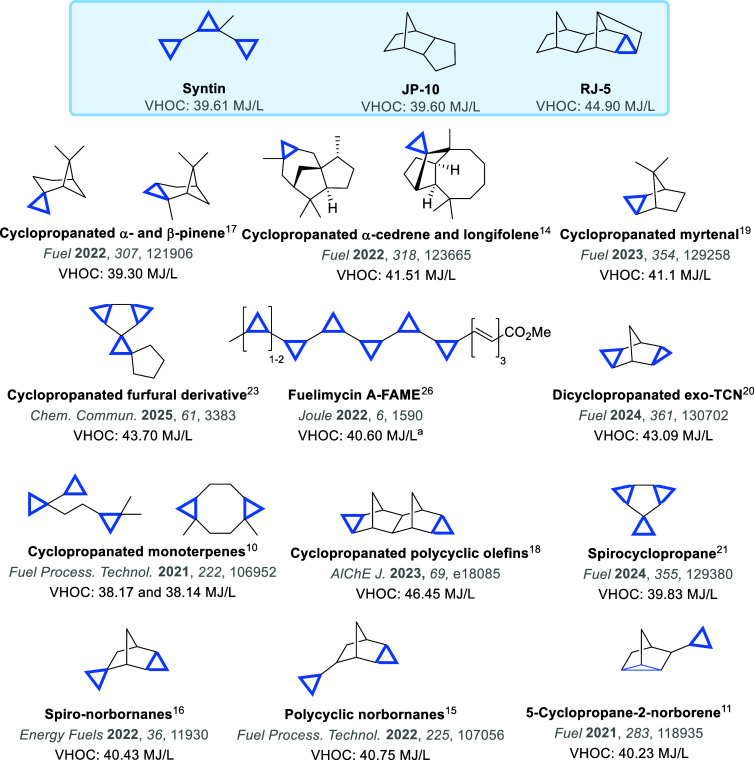
Structures and volumetric heats of combustion (VHOC) of conventional petroleum-derived HEDFs (blue box) compared with cyclopropane-containing synthetic HEDFs. Absolute stereochemistry of cyclopropane substituents is omitted for visual clarity, a theoretical value.

Building on our experience in designing lipid-inspired ionic liquids (ILs) with tailored properties for specific tasks,^27–29^ we have developed, for the first time, a homologous series of polycyclopropanated lipid-inspired ILs (PCP-ILs) as energy-dense fuel candidates. Starting from renewable fatty acid methyl esters, we employed a modular synthetic strategy, including Cu^I^-catalyzed azide–alkyne cycloaddition (CuAAC) click chemistry, that provides complete control over cyclopropanation, eliminating residual olefinic moieties (Figure 3). We hypothesized that PCP-ILs would combine the high energy density of polycyclopropanated structures with the inherent advantages of lipid-inspired ILs. These advantages include negligible vapor pressure that eliminates boiling points and evaporative losses while yielding high flash points for enhanced safety under ambient conditions, high thermal and chemical stability with ultralow melting/freezing points that enable operation across extreme temperature ranges, and long lipophilic side chains that provide additional carbons for fuel applications and increased density. Additionally, these organic materials offer tunable physicochemical properties through the strategic selection of cation–anion pairs and modification of lipophilic side chains.

**Figure 3:**
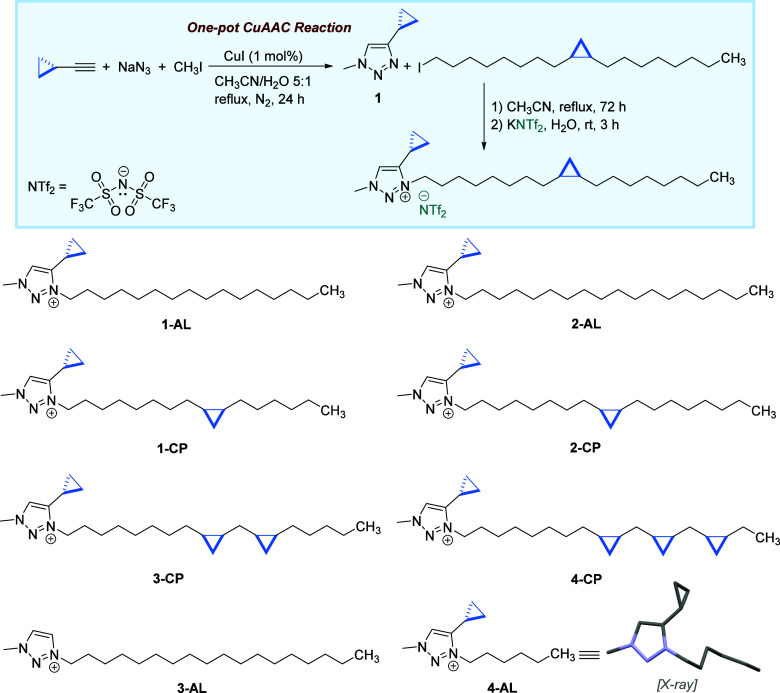
Top: synthetic pathway for the preparation of a PCP-IL, **2-CP**, demonstrating the representative method used to synthesize PCP-IL analogs. CuAAC click chemistry is employed to construct the 4-cyclopropyl-1-methyl-1,2,3-triazole headgroup (**1**). Bottom: structures of the synthesized cyclopropanated ILs. All compounds feature the [NTf_2_]^−^ counteranion, except **4-AL**, which contains [BPh_4_]^−^ (anion structure omitted for clarity). All cyclopropanes of the side chains have *cis* configurations.

## 2 Results and Discussion

### 2.1 Synthesis

To validate our hypothesis, we have synthesized a series of mono- and polycyclopropanated ILs through a multistep process using high-purity (99+%) fatty methyl esters and 4-cyclopropyl-1-methyl-1,2,3-triazole (**1**), as shown in Figure 3. Each of the ILs has a long-saturated alkyl appendage (C_16_ and C_18_), fully saturated side chains, and those containing one to three cyclopropane motifs, similar to structures found in natural fatty acids. This approach builds upon our previous work with lipid-like ILs inspired by homeoviscous adaptation (HVA) mechanisms in biological systems,^30^ where we demonstrated enhanced fluidity and increased lipophilicity.^28^ The cationic headgroup (**1**) was efficiently synthesized via a one-pot CuAAC reaction with near-perfect regioselectivity,^31,32^ incorporating a cyclopropyl moiety directly onto the cationic headgroup (Figure 3). The selection of 1,2,3-triazole as the cationic core was based on its synthetic accessibility through CuAAC click chemistry and its N—N—N arrangement, with the expectation of improved heat of combustion due to increased ring strain and electronic destabilization from the adjacent nitrogen atoms.

Crystals of compound **1** were successfully grown, confirming the formation of the cyclopropanated 1,2,3-triazole structure (Figures 4 and S3). The IL series were synthesized via the 3-*N*-substitution of headgroup **1** with different side chains: saturated palmityl (**1-AL,** C_16_) and stearyl (**2-AL**, C_18_) side chains; monocyclopropanated variants with a single cyclopropane motif at the nature-selected C9–C10 position [**1-CP** (C_16_), **2-CP** (C_18_)]; and PCP compounds with cyclopropane rings at C9–C10, C12–C13, and C15–C16 positions [**3-CP** (C_18_), **4-CP** (C_18_)]. This strategic placement of *cis*-cyclopropane motifs mimics nature’s symmetry-breaking positions while maximizing the potential energy density of these energy-dense fuel candidates.

**Figure 4:**
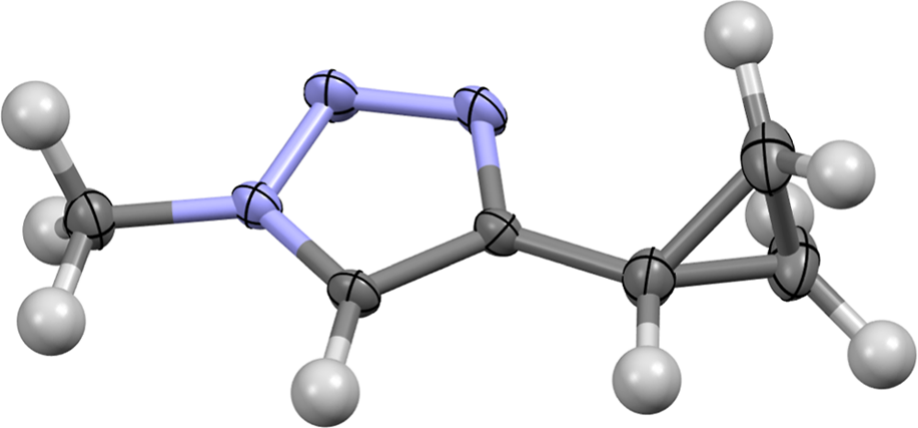
Asymmetric unit of compound **1** shown with 50% probability ellipsoids. Carbon = black; nitrogen = blue; hydrogen = gray.

The formation of our fully cyclopropanated ILs was established through Nuclear Magnetic Resonance (NMR) spectroscopy. We observed multiple signals characteristic of cyclopropane rings on the tails in the ^1^H NMR spectra, particularly the distinctive upfield-shifted resonances of the *exo*-methylene protons (*exo*-CH_2_) and their backbone methine protons. The ^13^C NMR analysis also confirmed the presence of *exo*-CH_2_ carbon signals at their characteristic chemical shift values with no residual alkene moieties observed. High-resolution mass spectrometry data provided additional evidence supporting the successful synthesis of our target ILs (see Supporting Information for the NMR and MS spectra).

### 2.2 Thermal and Thermodynamic Characterization

Melting points (*T*_m_) represent a critical thermophysical property for HEDFs (Figure 1), as low-temperature fluidity determines operational viability under cryogenic conditions. The hydrocarbon fuels demonstrate freezing points around −75 °C, while adamantyl-containing compounds exhibit significantly higher freezing points above −20 °C due to enhanced molecular packing in the solid state.^24^ Unlike enthalpy of combustion, which can be predicted *in silico* from bond energies, *T*_m_ values—particularly for ILs—arise from complex interplays between molecular packing, intermolecular forces, conformational flexibility, and entropic factors.^33,34^ To elucidate how structural modifications impact *T*_m_ values, we analyzed the relationship between the molecular structure and phase transitions through a systematic evaluation of thermodynamic parameters, determining whether changes in *T*_m_ are driven by enthalpic effects or entropic effects.

The synthesized ILs were studied by using Differential Scanning Calorimetry (DSC) to determine their thermophysical properties (Table 1 and Figure 5). To assess the cyclopropanation effect, we compared the *T*_m_ values of our ILs with the reference compound **3-AL** (1-methyl-1,2,3-triazolium cation with a stearyl tail). Due to high viscosity, all measurements required slow ramp rates and multiple heating/cooling cycles for accurate phase transition determination.

**Table 1:** Thermal Stability (*T*_onset5%_), Melting Point (*T*_m_), Glass Transition Temperature (*T*_g_), and Enthalpy (Δ_fus_ *H*) and Entropy (Δ_fus_ *S*) of Fusion Values^a^

compounds	*T*_onset5%_ (°C)	*T*_m_ (°C) ±0.1–0.3	*T*_g_ (°C) ±0.1–0.2	Δ_fus_ *H* (kJ mol^–1^) ±0.2–0.8	Δ_fus_ *S* (J mol^–1^ K^–1^) ±0.9–2.9
**1**	90.1	44.5	–	18.6	58.7
**1-AL**	333.0	23.5	–	39.4	132.8
**2-AL**	315.5	40.9	–	32.5	103.5
**3-AL**	322.0	49.2	–	42.2	130.9
**1-CP**	308.1	–	7.1	–	–
**2-CP**	337.3	–56.5	–	–	–
**3-CP**	327.3	–	1.9	–	–
**4-CP**	311.3	–	2.3	–	–

a Uncertainties are calculated as the standard deviation of the mean. *T*_m_ values represent the onset of the thermal transition, while *T*_g_ values are determined from the midpoint of the transition region.

**Figure 5:**
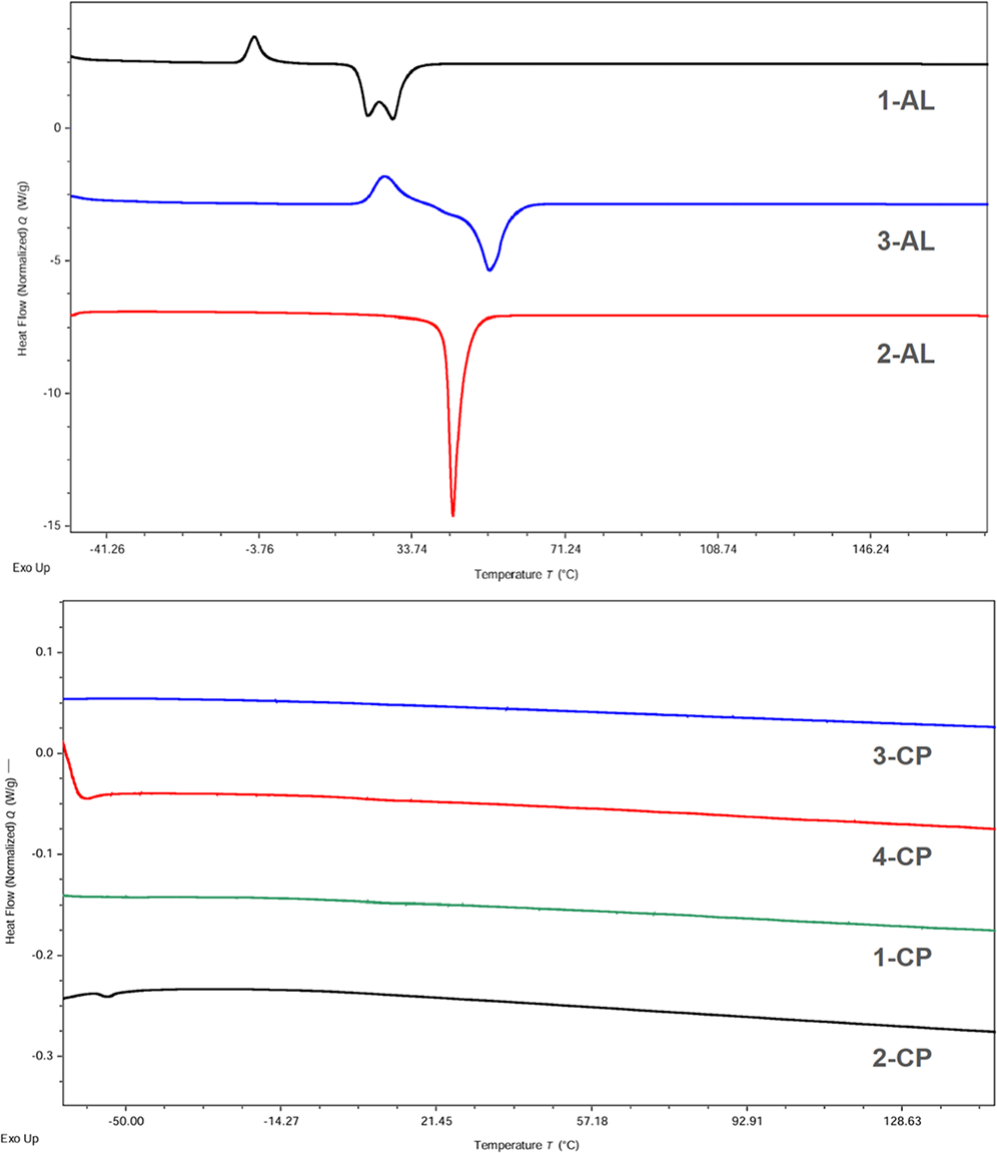
DSC thermograms for **1-AL**—**3-AL** (top) and **1-CP**—**4-CP** (bottom) compounds, with lines offset along the heat flow (*y*) axis for clarity but not rescaled.

Compounds **1-AL**—**3-AL** exhibited multifeatured sharp melting peaks typical of lipidic materials and ILs (Figure 5). IL **2-CP** exhibited a small endothermic peak similar to the first-order phase transitions (i.e., *T*_m_) observed in the **AL** salts. This behavior likely results from frustrated packing in the solid phase, where only a small fraction achieves crystalline order while most adopts a disordered glassy state, creating structural heterogeneity consistent with our previous observations.^29^ In contrast, ILs **1-CP, 3-CP**, and **4-CP** showed no first-order phase transitions, with featureless scans down to −90 °C (Figure 5). For **3-CP** and **4-CP**, the PCP-induced “kink” in the C_18_ side chain reduces chain order and packing efficiency, preventing crystallization and resulting in higher glass transition temperatures. For **1-CP**, we postulate that the shorter chain length (C_16_ vs C_18_) positions the cyclopropane group closer to the chain terminus, intensifying packing frustration by reducing alkyl-chain contacts (i.e., H···H interactions).

Table 1 shows that incorporating cyclopropanes into the stearyl side chain of 1,2,3-triazolium ILs substantially reduces *T*_m_ relative to that of the benchmark IL **3-AL** (Δ*T*_m_ = −105.7 °C). This reduction demonstrates how cyclopropyl moieties disrupt molecular packing and crystalline lattice formation, enhancing fluidity compared to saturated analogs. The cyclopropyl groups follow the HVA mechanism, where cyclopropanated lipids reduce melting temperatures to maintain membrane fluidity across thermal conditions, consistent with previous observations in cyclopropanated imidazolium-based ILs^29^ and fatty acids,^35^ as well as molecular dynamics (MD) simulations.^36^

To further elucidate the relationship between the structure and thermal behavior, we conducted a detailed thermodynamic analysis of phase transitions. For comparison, we examined the C_18_-based IL system, focusing on how cyclopropyl moieties in both the side chain and headgroup affect melting behavior. For each **AL** derivative, we systematically determined whether changes in *T*_m_ were primarily governed by enthalpy or entropy contributions. In general, *T*_m_ values of ILs can typically be modulated through two principal approaches: reduction of surface charge density of the constituent ions or diminution of molecular symmetry—both approaches directly influence the enthalpy (Δ_fus_ *H*) and entropy (Δ_fus_ *S*) of fusion.^37^ For comparative analysis, Table 2 lists relative changes in Δ*T*_m_ alongside the corresponding ratios of Δ_fus_ *H* and Δ_fus_ *S*, thereby enabling the identification of the predominant thermodynamic factor influencing *T*_m_ variations. While comparisons show substantial changes in Δ_fus_ *H* and Δ_fus_ *S* values, it is noteworthy that these thermodynamic shifts occur in opposite directions and consequently counterbalance each other’s impact on the resultant *T*_m_.

**Table 2:** Thermal and Thermodynamic Properties Comparison between C_18_-Based Cyclopropanated ILs and Compound **1** ^a^

comparison *i* vs ref	Δ*T*_ref_ *→*_*i*_ (°C)	Δ_fus,i_ *H*/Δ_fus,ref_ *H* × 100%	Δ_fus,i_ *S*/Δ_fus,ref_ *S* × 100%	driving force
**1-AL** vs **1**	–21.0	212	226	entropy-dominated *T*_m_ decrease
**2-AL** vs **1**	–3.6	174	176	entropy-dominated *T*_m_ decrease
**2-AL** vs **1-AL**	+17.4	82	77	entropy-dominated *T*_m_ increase
**3-AL** vs **2-AL**	+8.3	126	130	enthalpy-dominated *T*_m_ increase

a The data reveals that changes in melting behavior result from simultaneous and nearly offsetting shifts in both entropy and enthalpy of fusion, with both parameters changing in opposite directions.

The primary trend observed is the reduction in *T*_m_ values upon conversion of neutral compound **1** to its corresponding salt derivatives **1-AL** and **2-AL** (with Δ*T*_m_ values of −21.0 °C and −3.6 °C, respectively). Using compound **1** as our reference, the incorporation of long saturated side chains resulted in substantial increases in thermodynamic parameters: for **1-AL**, Δ_fus_ *H* increased by 112% and Δ_fus_ *S* by 126%, while for **2-AL**, Δ_fus_ *H* increased by 74% and Δ_fus_ *S* by 76% (Table 2). Despite strong Coulombic and van der Waals interactions, the *T*_m_ values decreased relative to compound **1**. This thermal behavior is predominantly governed by entropic factors attributed to the enhanced molecular asymmetry introduced by the triazolium cationic moiety. In fact, these findings align with recent MD studies demonstrating that entropic contributions from molecular asymmetry, particularly configurational entropy in the liquid state, are primarily responsible for the low melting points of ILs.^38^

In comparison to **2-AL**, **3-AL** exhibits a modest *T*_m_ increase of 8.3 °C due to the absence of the cyclopropyl group in the cationic headgroup (Table 2). The underlying thermodynamic mechanism remains enthalpy-driven, demonstrated by the modest offsetting changes in the fusion parameter: 30% increase in Δ_fus_ *H* and a 26% increase in Δ_fus_ *S* compared to **2-AL**. The reduced *T*_m_ for **2-AL** results from weakened electrostatic interactions between the ions. The cyclopropyl group adopts a perpendicular orientation to the triazolium ring, blocking approach to the π-system and disrupting headgroup stacking. This steric obstruction weakens cation–anion interactions, thereby lowering the melting point (see the Computational Studies Section).

Specific heat capacity (*C*_p_), measured by DSC, represents an important parameter for fuel evaluation, as it quantifies thermal energy absorption characteristics that directly influence combustion efficiency across various operating conditions. Conventional ILs typically exhibit *C*_p_ values between 1.200 and 1.900 J g^–1^ K^–1^,^39^ whereas our synthesized PCP-ILs demonstrated higher values. This enhancement aligns with established structure–property patterns in which bulkier or more flexible cation structures yield elevated heat capacity values.^40^ We observed a distinct positive correlation between the appended chain length and resulting *C*_p_ values (Figure 6).

**Figure 6:**
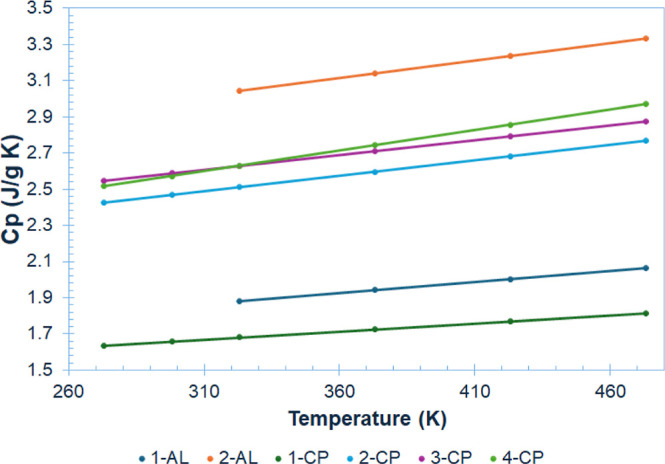
Effect of temperature on the specific heat capacity of the ILs.

HEDFs must exhibit sufficient thermal stability to resist decomposition upon contact with the heated metal surfaces encountered in fuel delivery systems and combustion chambers. We evaluated the thermo-oxidative stability of the compounds via gradient thermogravimetric analysis (TGA) performed under air to obtain data under conditions that more closely simulate real-world environments (Table 1 and Figure 6). We used the 5% mass loss temperature (*T*_onset5%_) as a benchmark for comparing the relative stabilities of these new PCP-IL materials. To maximize thermal stability, we selected bistriflimide ([NTf_2_]^−^) as the counteranion for our PCP-IL systems due to its high thermal stability with a decomposition temperature of ≥420 °C.^41^ We note that while [NTf_2_]^−^ is not suitable for fuel applications due to its fluorine and sulfur content, it serves as an ideal proof-of-concept anion to establish structure–property relationships for the first generation of this new organic-ion materials platform.

The data revealed excellent thermal stability across our compounds, with *T*_onset5%_ ranging from 308.1 to 337.3 °C (Table 1 and Figure 7). A similar IL with a monocyclopropanated C_18_ side chain demonstrated superior performance as a stationary phase for GC × GC analysis of kerosene, exhibiting the highest thermal stability among all ILs evaluated in this study.^42^ The primary decomposition pathway likely involves cleavage of the C—N^+^ bond, resulting in separation of the headgroup from the side chains through a Hofmann E2 elimination.^43^ As expected, neutral headgroup **1** exhibited markedly lower thermal stability (*T*_onset5%_ = 90.1 °C) compared to the IL products, which is primarily attributed to its volatility and subsequent evaporation under thermal stress rather than chemical decomposition.

**Figure 7:**
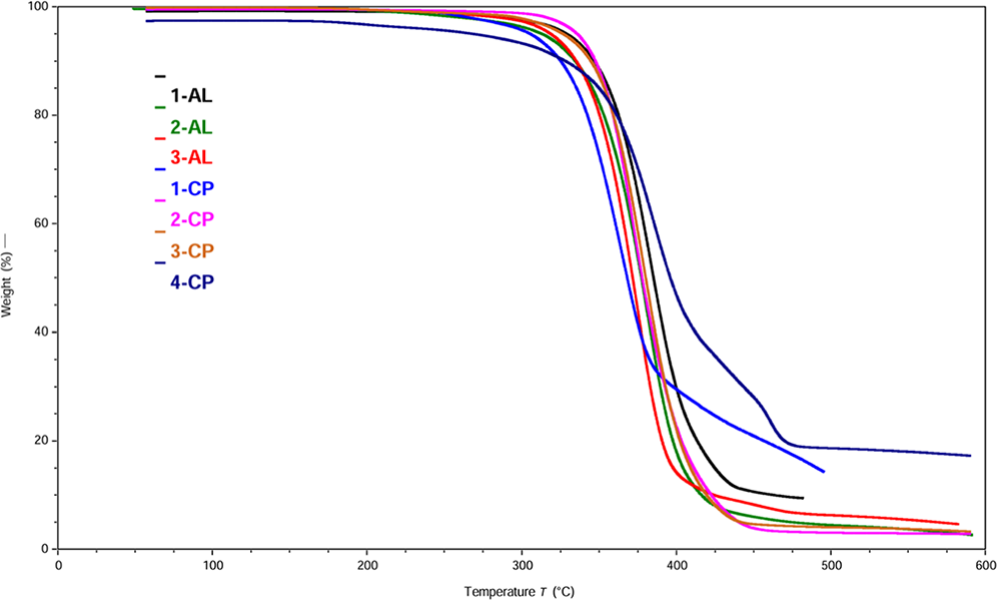
TGA of the synthesized ILs in air. These salts exhibit high stability above 300 °C under aerobic conditions.

### 2.3 Computational Studies

All IL cations were initially drawn in Ketcher and subjected to an initial 3D optimization with RDKit. Given the high flexibility of the long side chains, conformer searches were performed using the GOAT algorithm^44^ in ORCA 6.0 ^45^ with the semiempirical XTB method.^46^ These searches identified hundreds of low-energy conformers within 12 kJ/mol (3 kcal/mol) of the minimum. The lowest-energy conformer was then reoptimized at the B3LYP/6-31++G­(d,p) level in Gaussian 16. Benchmarking of B3LYP/6-31++G­(d,p), G3MP2B3, and M06-2*X*/6-31 + G­(d,p) against experimental heats of combustion (Figure S4) showed that M06-2X offers the best balance of accuracy and computational feasibility for these large systems; this level of theory was therefore used throughout.

The [NTf_2_]^−^ anion was optimized at the same computational level. Ion pairs were constructed using PACKMOL^47^ to position the anion around the 1,2,3-triazolium headgroup. The resulting ion pairs underwent conformer searches with the GOAT/XTB method in ORCA, and the lowest-energy structures were reoptimized at the B3LYP/6-31++G­(d,p) level in Gaussian 16. Pairing energies (Δ*E*_pair_) were calculated as the difference between the energy of the ion pair and the sum of the isolated ions.

Representative optimized structures illustrate the structural and electronic changes resulting from the cyclopropanation of the side chains. A comparison of the same triazolium headgroup with a linear alkyl side chain (**2-AL**) and a fully cyclopropanated side chain (**4-CP**) is shown in Figure 8. The cyclopropyl groups introduce substantial bending and kinking in the chain, providing computational validation for the disrupted packing and reduced melting point values observed in the DSC measurements.

**Figure 8:**
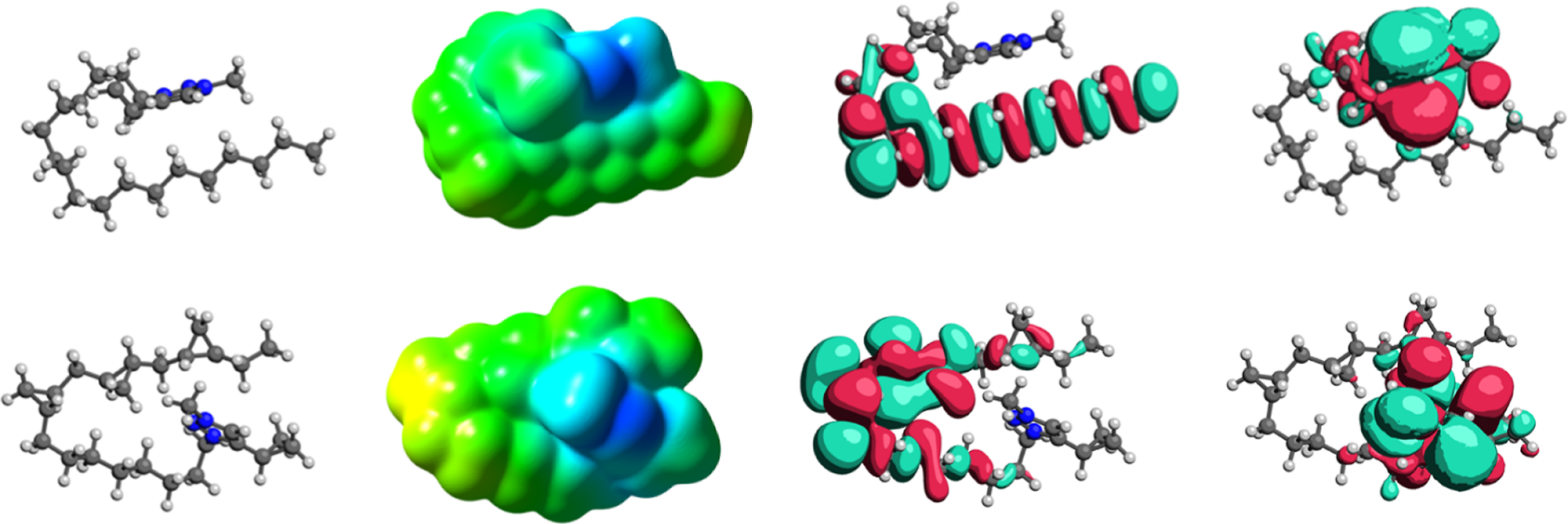
Optimized ion-pair geometries, ESP surfaces, and frontier orbitals (HOMO/LUMO) for **2-AL** (top) and **4-CP** (bottom) at the M062*X*/6-31 + G­(d,p) level.

Overall, the electronic effects are modest. Electrostatic potential (ESP) maps show only a slight charge density redistribution near the cyclopropanated sites, while HOMO/LUMO plots reveal no significant changes aside from minor orbital reorganization. Additional comparisons of the other cations (**2-AL**, **3-AL**, **2-CP**, **3-CP**, and **4-CP**) are provided in the Supporting Information.

Representative optimized geometries of the cation–anion pairs are shown in Figure 9. The structures indicate that cyclopropanation substantially alters cation–anion organization. In all cases, the oxygens of the [NTf_2_]^−^ anion localize near the triazolium ring, with the cation’s side chain folding back toward the anion. For **3-AL**, the side chain wraps smoothly and fully around the anion. In **2-AL**, the tail adopts an irregular serpentine wrapping. In **4-CP**, the multiple kinks introduced by the cyclopropyl groups block the chain from enclosing the anion. This behavior parallels the DSC results, where cyclopropanation disrupts packing and favors glass formation over crystallization.

**Figure 9:**
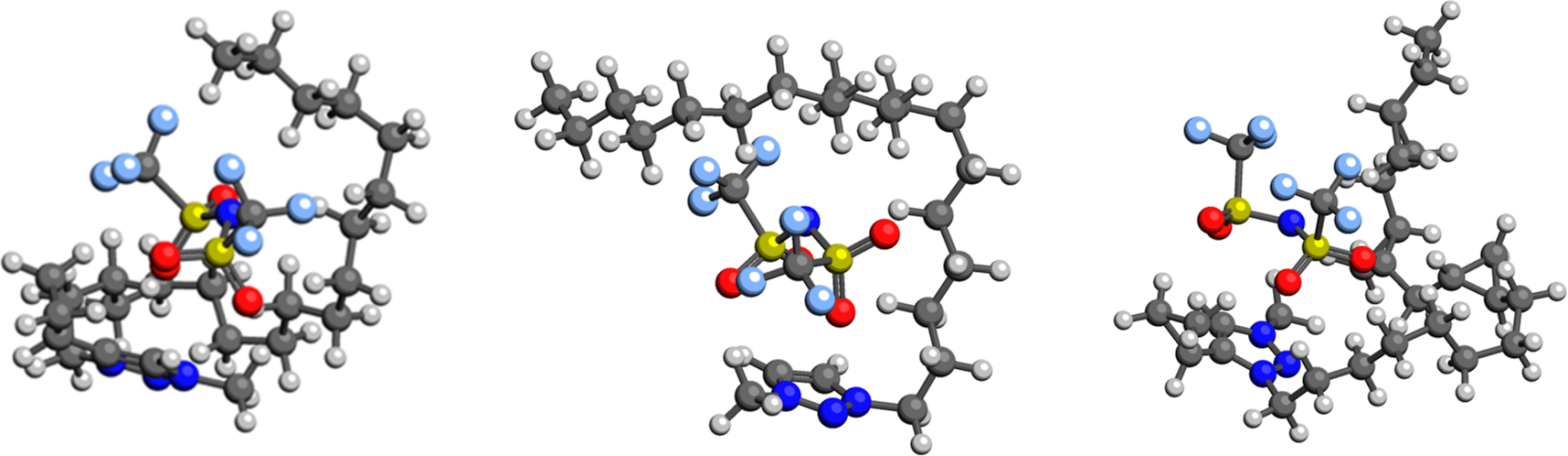
Gas-phase minimum-energy structures of the optimized ion pairs at the M062*X*/6-31 + G­(d,p) level. For **3-AL** (left), **2-AL** (middle), and **4-CP** (right).

The calculated pairing energies across all cation–anion pairs remain closely grouped within a narrow range of −343 to −360 kJ/mol. Therefore, the primary effect of cyclopropanation is conformational frustration rather than weakened cation–anion binding interactions.

Energetically, the mass-normalized combustion enthalpies of gas-phase pairs increase linearly with each additional cyclopropyl group (Table 3). From **2-AL** to **4-CP**, there is a consistent increment of ∼0.3 kJ/g per cyclopropyl unit, rising from 21.9 to 22.8 kJ/g. The pairing energies remain confined to the aforementioned narrow range, confirming that cyclopropyl substitution affects molecular packing arrangements more significantly than the ionic binding strength. The energetic contribution of cyclopropanation arises primarily from the release of ring strain during combustion, while its dominant structural effect involves the disruption of molecular packing, providing computational validation for the thermal behavior observed in DSC measurements.

**Table 3:** Calculated Gas-Phase Ion-Pairing Energies and Estimated Standard-State Gas-Phase Combustion Enthalpies for Each IL Pair with C_18_ Tails^a^

IL	*M*_w_ (g mol^–1^)	Δ*E*_pair_ (kJ mol^–1^)	Δ_c_ *H* ^o^ (MJ mol^–1^)	Δ_c_ *H* ^o^_mass_ (kJ g^–1^)
**3-AL**	616.72	–360.4	–12.8	–20.7
**2-AL**	656.79	–348.2	–14.4	–21.9
**2-CP**	668.80	–345.3	–14.8	–22.2
**3-CP**	680.81	–342.5	–15.3	–22.5
**4-CP**	692.82	–356.4	–15.8	–22.8

a Pairing energies (Δ*E*_pair_) were computed as the difference between ion-pair and isolated ion electronic energies at the M062*X*/6-31+G­(d,p) level. Combustion enthalpies (Δ_c_ *H* ^o^) were calculated from thermal enthalpies and balanced stoichiometric combustion reactions in the gas phase.

### 2.4 Enthalpy of Combustion Calculations

We employed density functional theory (DFT) calculations to determine the enthalpy of combustion for the PCP-ILs. The enthalpy of combustion (Δ_c_ *H*°) was determined from balanced combustion reactions and all combustion species (O_2_, CO_2_, H_2_O, HF, N_2_, and SO_2_) were optimized at the same level of theory (see Supporting Information). Thermal enthalpies were obtained from frequency calculations.

To establish a reliable computational method for calculating the heats of combustion, we first benchmarked three common DFT approaches: B3LYP/6-31++G­(d,p), G3MP2B3, and M06–2*X*/6-31 + G­(d,p) against the benchmark HEDFs illustrated in Figure 2 with well-established volumetric heats of combustion (VHOC). The comparison (Figure S4) showed a clear trend: M06-2*X*/6-31 + G­(d,p) consistently matched the experimental data most closely, especially for strained hydrocarbons, where bond-strain release dominates the combustion energy.

Our theoretical calculations reveal that cyclopropanated ILs containing C_18_ side chains exhibit enthalpy of combustion values comparable to those of conventional HEDFs (Table 3). For comparison, conventional aviation fuels such as JP-10 and RJ-5 have gravimetric heats of combustion of 42–43 kJ/g (39.6 MJ/L–44.9 MJ/L), respectively.^48^ Although the PCP-ILs studied here have somewhat lower gravimetric energies of 21.9–22.8 kJ/g, their typically high liquid densities (>1.3 g/mL for similar salts^49^) lead to volumetric energy densities on the order of ∼30 MJ/L, demonstrating that significant energy can be stored in dense, ionic structures. The systematic increase in Δ_c_ *H*° observed upon incorporating additional cyclopropyl groups to both the headgroup ring and side chain (**4-CP** vs **3-AL**) validates our initial hypothesis that cyclopropyl functionalization enhances energetic performance. Notably, the counterion is essential for maintaining low *T*_m_ and liquid-state properties characteristic of ILs, inherently dilutes the overall energy density relative to pure hydrocarbon fuels—a trade-off between energetic performance and the practical handling advantages (negligible vapor pressure, thermal stability, and nonflammability) that IL provide. Thus, while their specific energies are modest, cyclopropanation offers a systematic route to increase energy content, and when coupled with the high density and tunable phase behavior of these salts, positions PCP-ILs as strong candidates where volumetric performance and thermal robustness are critical.

Beyond their energetic properties, PCP-ILs offer several practical advantages for demanding environments. They have negligible vapor pressure, high thermal stability in air, greater oxidative stability than their olefinic counterparts, and melting points that can be tuned over a broad range. Together, these attributes outline a new design space: low-*T*_m_, tunable ionic fuels that pair modest but systematic energy gains from cyclopropanation with exceptional stability and handling.

### 2.5 X-ray Crystallographic Analysis

To better understand the structure–property relationships of our ILs, we conducted single crystal X-ray diffraction (SCXRD) studies. Despite numerous attempts under various crystallization conditions, our synthesized ILs did not yield suitable single crystals for X-ray analysis. We therefore adopted an alternative strategy by synthesizing a shorter-chain analog (**4**-**AL**) containing a C_6_ saturated alkyl chain paired with the [Ph_4_B]^−^ anion—an approach we have successfully employed in previous studies to obtain crystallographic data when cyclopropanated ILs do not readily crystallize.^27,29^ Using the same synthetic methods (Figure 2), **4-AL** formed suitable single crystals at room temperature (Figure 10). The resulting crystal structure clearly revealed the characteristic kinking effect induced by the cyclopropyl, which disrupts the molecular symmetry compared with compound **1**. These structural features observed in the shorter-chain cation—particularly the irregular side chain conformations—likely represent torsional characteristics that would also be present in our longer-chain IL analogs, providing valuable insights into their molecular organization and packing behavior.

**Figure 10:**
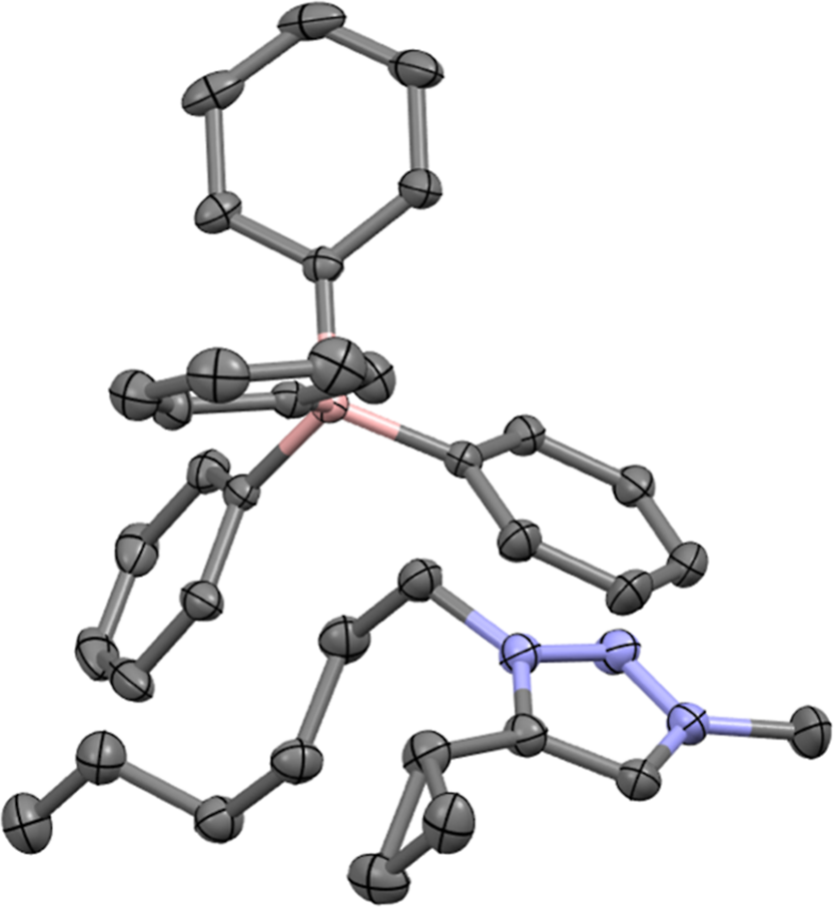
Asymmetric unit of compound **4-AL** shown with 50% probability ellipsoids. Hydrogens and disorder are omitted for clarity. Carbon = gray; nitrogen = blue; and boron = pink.

We examined the crystal structure of a prototypical cyclopropanated IL with the asymmetric unit containing a single cation–anion pair (Figure 10). This structure is particularly significant when compared with the extensive literature on imidazolium-based ILs. While the Cambridge Structural Database (CSD)^50,51^ contains hundreds of dialkylated imidazolium-based IL crystal structures, only 18 dialkylated 1,2,3-triazolium-based crystal structures have been documented to date. The majority of the reported structures come from Mudring and colleagues and establish foundational insights into the structure and properties of ILs bearing this cation.^52–54^ Given the novelty of the cationic core, the structure reported here offers a unique opportunity to evaluate not only how cyclopropyl incorporation affects the cation core but also the resulting inter- and intramolecular interactions. Four key structural features emerge from our analysis.

First, the cation exhibits positional disorder in the alkyl chain—a common feature in IL cations that contributes to their low melting points, especially for shorter chains (*C*_*n*_ ≤ 7).^28^ In **4-AL**, the cyclopropyl ring introduces additional complexity into this disorder. Detailed examination of the disordered fragments (Figure S2) reveals that the cyclopropyl ring constrains the alkyl chain’s rotational freedom through steric interactions between the ring’s C–H groups and the alkyl chain. This constraint is evidenced by the chain’s disorder occurring concurrently with rotation of the cyclopropyl ring. Consequently, carbons C8–C9–C10–C11 in the alkyl chain adopt *gauche* conformations with torsion angles of −67.54° and 69.86° in the alternate rotational orientations. As previously established,^55^ these variations in chain conformations within the symmetry-breaking region are crucial for disrupting long-range order and frustrating crystal packing.^28^ Thus, the cyclopropyl ring bound to the cation appears to influence the formation of gauche conformations of the alkyl chain.

Second, the cyclopropyl ring engages in multiple close-contact interactions with symmetry-related cationic and anionic units. Figure 10 highlights interactions shorter than the sum of van der Waals radii involving the cyclopropyl portion of the cation. These interactions include H···C|C···H (π interactions) and H···H contacts with neighboring ions with a notable preference for H···C|C···H interactions involving the π-systems of the anion’s phenyl rings (Figure 11).

**Figure 11:**
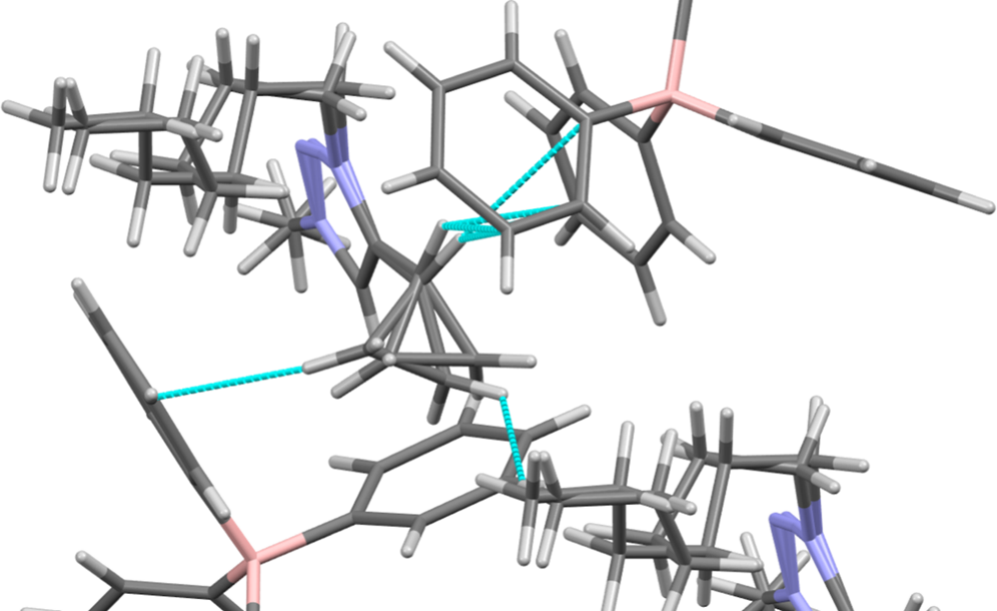
Depiction of the H···C | C···H and H···H interactions with adjacent anions and cations arising from the cyclopropyl moiety on the cation in **4-AL**. Interactions are shown in blue and are less than or equal to the vdW radii of the atoms. Only portions of the surrounding molecules are shown for clarity. Disorder is intentionally included in the image.

Third, from a molecular engineering perspective for IL cations, close contacts involving aromatic hydrogens are often pivotal interionic interactions, particularly for the C2–H in imidazolium cations.^56^ The incorporation of the cyclopropyl ring on the triazolium core eliminates a potential aromatic C–H hydrogen-bond donor site. Moreover, the increased steric bulk from the cyclopropyl ring likely impedes, or at least weakens, the formation of certain cation–anion interactions on the adjacent aromatic ring via steric blocking of the π system of the triazolium moiety. Nonetheless, the remaining aromatic C–H moiety still interacts with the π-system of a neighboring phenyl ring. Importantly, while cyclopropyl incorporation restricts some traditional cation–anion contacts, it simultaneously introduces new intermolecular interactions as described above.

Finally, the central N2 atom accounts for approximately 3.5% of the cation-based interactions (Figure 12), primarily through H···N|N···H contacts with symmetry-related aromatic C–H units on the anion. However, the variable angles and distances of these N···H interactions suggest that they are incidental outcomes of other dominant stabilizing forces within the crystal lattice rather than hydrogen bonds.

**Figure 12:**
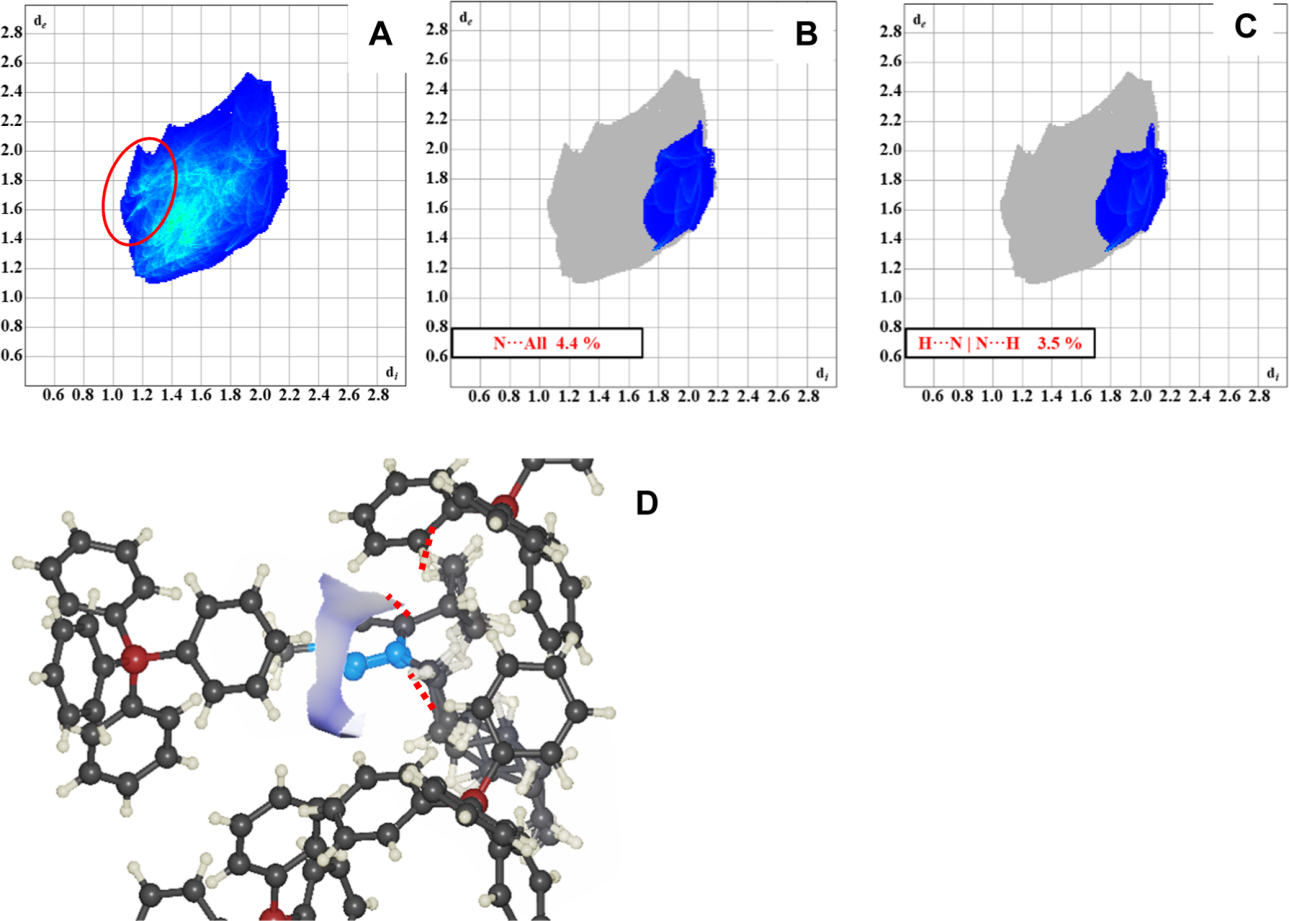
(A) Complete interaction fingerprint for **4-AL**. Features such as π interactions manifest as the wings (indicated region). (B) Fingerprint of interactions arising only from N atoms on the cation and (C) fingerprint of only the H···N|N···H interactions, which are depicted in (D). The portion of the Hirshfeld surface corresponding to these interactions is shown as the blue and white surface in D.

## 3 Experimental Section

The detailed synthetic procedure and the crystallographic and computational methods used in this work are described in the Supporting Information.

## 4 Conclusions

In conclusion, we have developed a novel class of polycyclopropanated ionic liquids (PCP-ILs) from renewable fatty acid feedstocks that demonstrate promising performance as high-energy-density fuels. These lipid-inspired materials exhibit negligible vapor pressure, tunable ultralow melting points, and high thermal stability, properties that are challenging to achieve simultaneously in conventional fuel systems. X-ray crystallographic analysis, thermodynamic studies, and DFT calculations reveal that incorporated cyclopropyl groups significantly reduce melting temperatures through the disruption of solid-state packing and increased alkyl chain kinking, paralleling observations in analogous fatty acids.

The PCP-ILs deliver volumetric energy densities of ∼30 MJ/L, competitive with conventional aviation fuels, while offering superior handling characteristics, including negligible vapor pressure and enhanced oxidative stability relative to hydrocarbon fuels. The synthetic methodology achieves complete cyclopropanation at targeted positions on the fatty acid chains, providing structural control beyond current biosynthetic approaches. Structural studies demonstrate that cyclopropyl substitution on the cationic headgroup further lowers melting points, establishing clear design principles for tuning thermal properties in this materials platform.

While the [NTf_2_]^−^ anion used in this proof-of-concept study can generate acidic decomposition products at elevated temperatures (e.g., HF and SO_*x*_),^57^ future implementations require high-energy, thermally stable, sulfur- and fluoride-free, and cost-effective anions^58,59^ that form low-melting IL systems to unlock the full potential of this materials platform. Our laboratories continue to actively pursue the systematic development of improved PCP-IL systems as a foundation for developing the next generation of sustainable HEDF alternatives.

## Supplementary Material





## Data Availability

The data supporting this study are available within the article and its Supporting Information (SI). Crystallographic data were deposited with the Cambridge Crystallographic Data Centre (CCDC) under deposition numbers 2491537 and 2491538.

## Notes

Synthesis and characterizations of the new compounds (^1^H, ^13^C, and ^19^F NMR spectra, and MS data); DSC,TGA, and *C*_p_thermograms; and crystallographic and computational data (PDF)
CIFs for compounds **1** and **4-AL** with check CIF reports (ZIP)

## Notes

This article is dedicated to the memory of Dr. Barbara P. Shineman (1928–2024), whose generous contributions have had immeasurable impacts on research and education at SUNY Oswego and beyond.
