# Supplementary material for: Polycyclopropanated Lipid-Inspired Ionic Liquids as High Energy-Density Fuel Candidates
Source: ACS Sustain Chem Eng. 2026 Feb 23;14(9):4596–607. doi: 10.1021/acssuschemeng.5c13132 (PMC12977155; doi:10.1021/acssuschemeng.5c13132)
Supplement: Supplementary file 1 [file sc5c13132_si_001.pdf]

SUPPORTING INFORMATION

Polycyclopropanated Lipid-Inspired Ionic Liquids as High Energy Density Fuel Candidates

Christopher M. Butch,[a] Richard A. O'Brien,[b] Raychell A. Jerdo,[a] James H. Davis, Jr.,[b] Matthias Zeller,[c] Brooks D. Rabideau,*[,d] Patrick C. Hillesheim,*[,e] Arsalan Mirjafari*[,a]

[a] Department of Chemistry, State University of New York at Oswego, Oswego, New York 13126, United States

[b] Department of Chemistry, University of South Alabama, Mobile, Alabama 36688, United States

[c] Department of Chemistry, Purdue University, West Lafayette, Indiana 47907, United States

[d] Department of Chemical & Biomolecular Engineering, University of South Alabama, Mobile, Alabama 36688, United States

[e] Department of Chemistry, Illinois State University, Normal, Illinois 61761, United States

Supporting Information Summary:

- **Pages:** 46
- **Figures:** 10 (Figures S1-S10)
- **Tables:** 2 (Tables S1-S2)

Additional Content:

- NMR spectra (^1H, ^{13}C, and ^{19}F) for all compounds
- DSC and TGA thermograms for all products
- Heat capacity measurements for all compounds
- Crystallographic data and computational details

Corresponding Authors:
Arsalan Mirjafari, arsalan.mirjafari@oswego.edu
Patrick Hillesheim, pchille@ilstu.edu
Brooks Rabideau, brabideau@southalabama.edu

EXPERIMENTAL PROCEDURES AND CHARACTERIZATION DATA

Materials and Instrumentation

All commercial chemicals were purchased at the highest quality and used as received unless otherwise noted. Commercial methyl esters of the C_{16} and C_{18} olefins were purchased from Nu-Chek Prep (99%+ purity). Solvents such as cyclohexane, hexanes, acetonitrile (CH_3CN), ethyl acetate (EtOAc), and dichloromethane (DCM) were obtained from Acros Organics and Aldrich Chemical.

Structural characterizations of the final cyclopropanated ionic liquids for ^{1}H and ^{13}C NMR were performed on a BRUKER 300 and 500 MHz NMR using $CDCl_3$ as the deuterated solvent ($CDCl_3$ ^{1}H NMR = 7.28 ppm, ^{13}C NMR = 77.45, 77.03, 76.60). The following abbreviations were used to explain NMR peak multiplicities: s = singlet, d = doublet, t = triplet, q = quarter, m = multiplet.

The mass spectrometry (MS) data were obtained using a Thermo Scientific Altis TSQ triple-quadrupole mass spectrometer. Samples were prepared at 10 ppm in LCMS grade acetonitrile (Fisher Optima) and introduced into the MS directly by syringe pump set to mix 5 uL/min of this solution into a 0.2 mL/min flow from the HPLC consisting of acetonitrile with 0.1% formic acid (Fisher Optima). Data were collected using the automated optimization for "selected reaction monitoring" (SRM) analysis in the Chromeleon software, using argon (1.5 mTorr) in the collision chamber and a capillary temperature of 325 °C. Each type of gas flow was optimized for each molecular ion and was within a 20% range of the default values.

The reported optimal isolation mass for the molecular m/z is the peak of this band; this is a low-resolution mass spectrometer, so the mass agreement is uniformly excellent. This was optimized for source voltage (CID), which was 0 V, except where otherwise noted. The capillary voltage (VCAP) was also optimized and is reported. The top 5 SRM reactions are reported with their max intensity at optimized collision energy/ voltage (CV) for that product. These are reported with their relative maximum intensity as well as the intensity of the SRM ion relative to the molecular ion maximum intensity at CV = 0.

Melting points, glass transition temperatures, and specific heat capacities were measured using a TA Discovery 250 DSC, calibrated using indium (melting point) and sapphire (heat capacity) references.

Thermogravimetric analyses were performed on a TA instrument TGA 550 under air flow using a platinum pan. The samples were heated from room temperature at a rate of 10 °C/min to a maximum temperature of 600 °C under air.

Single crystal XRD experiments were carried out with a Bruker AXS D8 Quest diffractometer with a PhotonII charge-integrating pixel array detector (CPAD) and Mo-*Kα* sealed X-ray tube. Absorption was corrected by multi-scan methods using *SADABS*. Additional details are provided in the Crystallographic Data section.

General Synthetic Procedures

*Synthesis of 4-cyclopropyl-1-methyl-1,2,3-triazole (**1**)*

An oven-dried 250 mL round bottom flask was charged with cyclopropyl acetylene (2.72 mL, 0.03 mol, 1.0 equiv.), sodium azide (2.08 g, 0.03 mol, 1.0 equiv.), methyl iodide (2.00 mL, 0.03 mol,

1.0 equiv.), CuI (0.07 g, 1 mol%), and a 5:1 mixture of CH_3CN:H_2O (100:20 mL). The reaction was stirred and refluxed under a continuous flow of N_2 gas for 24 hours. The resulting mixture was transferred to a 500 mL Erlenmeyer flask containing 200 mL of 2.0 M $NH_4OH_{(aq)}$ and stirred for 30 minutes. The product was extracted with DCM (3 × 100 mL), and the combined organic extracts were dried over anhydrous $MgSO_4$. The drying agent was removed by vacuum filtration, and the solvent was removed *in vacuo* to yield 3.72 g (96%) of pure product as off-white powder. TLC analysis indicated the complete consumption of the reactants and the formation of a single product. Recrystallizations with cyclohexane instantly afforded needle-like crystals (Figure S1).

T_m = 44.5 ºC; TLC: Rf = 0.23 (50% EtOAc in hexanes); ^{1}H NMR (300 MHz, $CDCl_3$): δ_H 7.22 (s, 1H), 4.05 (s, 3H), 2.01-1.92 (m, 1H), 0.98-0.84 (m, 4H). ^{13}C NMR (500 MHz, $CDCl_3$): δ_C 150.2, 121.0, 36.8, 7.9, 6.4.; MS (ESI): Calcd for $C_6H_9N_3$ + H^+ $[M+H]^+$: 124.080, found 124.080.

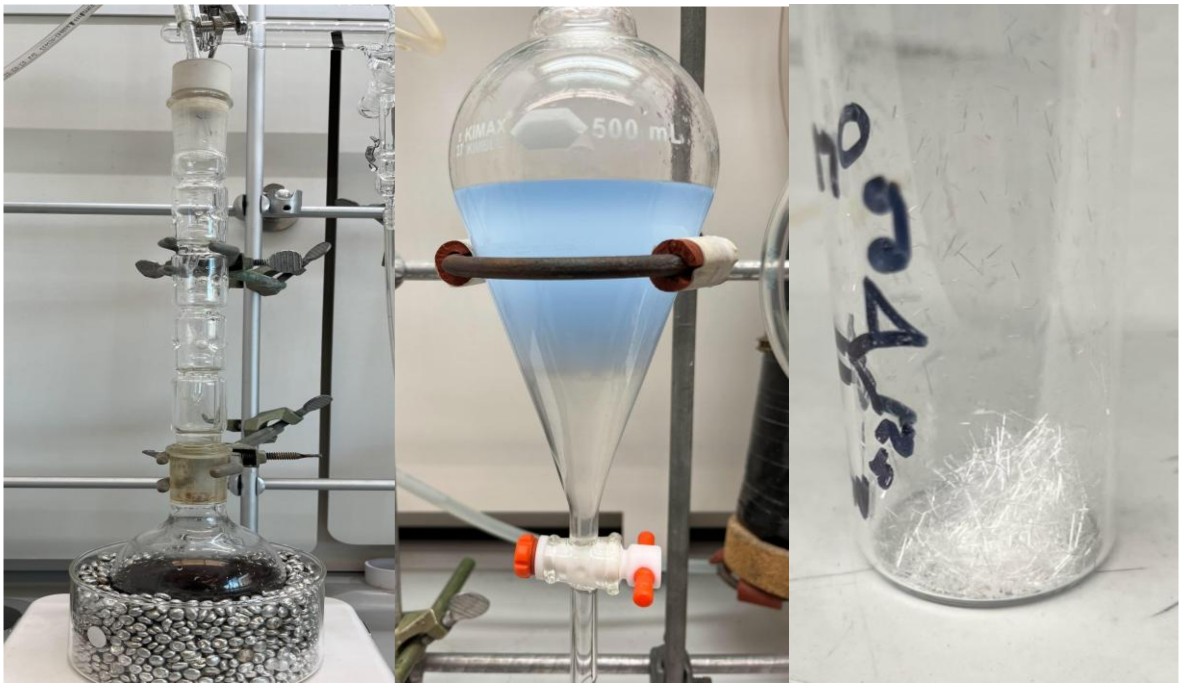

Figure S1. Left: reaction set-up of the one-pot synthesis. Middle: liquid-liquid extraction between DCM and 2.0 M NH_4OH. Right: Sample of the final product.

*Synthesis of ILs **1-AL** and **2-AL***

In an oven-dried 100 mL round bottom flask equipped with a stir bar, 4-cyclopropyl-1-methyl-1,2,3-triazole (0.30 g, 0.003 mol, 1.2 equiv.) and 1-bromohexadecane/1-bromooctadecane (0.0021 mol, 1.0 equiv.) were dissolved in CH_3CN (60 mL). The reaction was refluxed for 72 hours, and the solvent was then removed *in vacuo* to afford yellowish solids. Next, in an oven-dried 250 mL Erlenmeyer flask equipped with a stir bar, the bromide salt from the previous crude reaction mixture (1.0 g, 0.002 mol, 1.0 equiv.) and potassium triflimide (0.003 mol, 1.1 equiv.) were dissolved in deionized water (100 mL). The reaction was stirred at ambient temperature for three hours, during which time the product precipitates. The aqueous phase is carefully decanted, and the precipitate is washed with deionized water (3 × 50 mL) and hexanes (5 × 30 mL). During each

wash, the mixture is cooled in a refrigerator to minimize emulsion formation. Residual hexanes were removed *in vacuo* to afford the desired products as a white solid in excellent yield.

1-AL. T_m = 23.5 ºC; ^{1}H NMR (500 MHz, $CDCl_3$): δ_H 8.09 (s, 1H), 4.56-4.53 (t, *J* = 7.5 Hz, 2H), 4.29 (s, 3H), 2.05-1.92 (m, *J* = 7.4 Hz, 2H), 1.91-1.87 (td, *J* = 8.3, 4.1 Hz, 1H), 1.39-1.28 (m, 26H), 1.05-1.04 (m, 4H), 0.91-0.89 (t, *J* = 6.9 Hz, 3H); ^{13}C NMR (500 MHz, $CDCl_3$): δ_C 146.3, 127.7, 123.6, 121.1, 118.5, 116.0, 51.3, 40.1, 31.9, 29.7, 29.6, 29.6, 29.5, 29.3, 29.3, 29.1, 28.8, 28.6, 26.2, 26.1, 22.7, 14.1, 8.2, 8.0, 3.9, 3.8; δ_F -78.9; MS (ESI): Calcd for $C_{22}H_{42}N_3^+$ [M+H]$^+$: 348.337, found 348.310.

2-AL. T_m = 40.9 ºC; ^{1}H NMR (500 MHz, $CDCl_3$): δ_H 8.16 (s, 1H), 4.56-4.53 (t, *J* = 7.6 Hz, 2H), 4.31 (s, 3H), 2.06-2.03 (m, *J* = 7.5 Hz, 2H), 1.89-1.86 (m, 1H), 1.43-1.28 (m, 32H), 1.09-1.07 (dd, *J* = 5.0, 1.7 Hz, 2H), 0.92-0.89 (t, *J* = 7.0 Hz, 3H); ^{13}C NMR (500 MHz, $CDCl_3$): δ_C 146.3, 127.9, 123.6, 121.1, 118.5, 116.0, 60.3, 51.3, 40.2, 31.9, 29.7, 29.6, 29.6, 29.6, 29.5, 29.3, 29.3, 29.2, 28.8, 28.6, 28.6, 26.3, 22.7, 21.0, 14.2, 14.0, 8.0, 3.8; δ_F -78.8; MS (ESI): Calcd for $C_{24}H_{46}N_3^+$ [M+H]$^+$: 376.368, found 376.350.

Synthesis of ILs ***1-CP – 4-CP***

In an oven-dried 100 mL round bottom flask equipped with a stir bar 4-cyclopropyl-1-methyl-1,2,3-triazole (0.20 g, 0.002 mol, 1.2 equiv.), and mono and polycyclopropanated alkyl iodide[1] (1 equiv.) were dissolved in CH_3CN (60 mL). The reaction was refluxed for 72 hours and the solvent was then removed *in vacuo* to afford a dark brown liquid. Next, in an oven-dried 250 mL Erlenmeyer flask equipped with stir bar, the iodide salt from the previous crude reaction mixture (0.001 mol, 1.0 equiv.) and potassium triflimide (0.001 mol, 1.1 equiv.) were dissolved in deionized water (100 mL). The reaction was stirred at ambient temperature for 3 hours, during which time the product precipitates. The aqueous phase is carefully decanted, and the precipitate is washed with deionized water (3 × 50 mL) and hexanes (5 × 30 mL). During each wash, the mixture is cooled in a refrigerator to minimize emulsion formation. Residual hexanes were removed *in vacuo* to afford the desired products as a viscous, amber-colored liquid.

1-CP. T_g = 7.1 ºC; ^{1}H NMR (500 MHz, $CDCl_3$): δ_H 8.18 (s, 1H), 4.57-4.54 (t, *J* = 7.6 Hz, 2H), 4.34 (s, 3H), 2.07-2.04 (m, *J* = 7.5 Hz, 2H), 1.89-1.86 (m, 1H), 1.41-1.32 (m, 20H), 1.17-1.15 (d, *J* = 6.9 Hz, 2H), 1.10-1.09 (d, *J* = 4.9 Hz, 2H), 0.93-0.90 (t, *J* = 7.0 Hz, 3H), 0.69-0.66 (q, *J* = 5.3 Hz, 2H), 0.62-0.57 (td, *J* = 8.3, 4.0 Hz, 2H), -0.29 – -0.32 (q, *J* = 5.3 Hz, 2H); ^{13}C NMR (500 MHz, $CDCl_3$): δ_C 146.3, 127.9, 123.6, 121.1, 118.5, 116.0, 51.3, 40.2, 31.9, 30.2, 30.1, 29.5, 29.5, 29.3, 28.9, 28.7, 28.7, 28.7, 28.6, 26.3, 22.7, 15.8, 15.7, 14.1, 10.9, 8.0, 3.8; δ_F -78.9; MS (ESI): Calcd for $C_{23}H_{42}N_3^+$ [M+H]$^+$: 360.337, found 360.330.

2-CP. T_m = -56.5 ºC; ^{1}H NMR (500 MHz, $CDCl_3$): δ_H 8.15 (s, 1H), 4.56-4.53 (t, *J* = 7.5 Hz, 2H), 4.31 (s, 3H), 2.06-2.03 (m, *J* = 7.5 Hz, 2H), 1.90-1.87 (m, 1H), 1.40-1.30 (m, 26H), 1.16-1.15 (d, *J* = 6.3 Hz, 2H), 1.08-1.06 (m, 2H), 0.92-0.89 (t, *J* = 7.0 Hz, 3H), 0.61-0.57 (td, *J* = 8.1, 4.0 Hz, 2H), -0.29 – -0.32 (q, *J* = 5.4 Hz, 2H); ^{13}C NMR (500 MHz, $CDCl_3$): δ_C 146.3, 127.7, 123.6, 121.1, 118.5, 116.0, 51.3, 40.1, 31.9, 30.2, 30.1, 30.0, 29.7, 29.6, 29.4, 29.3, 29.3, 28.8, 28.7, 28.6, 28.6,

26.2, 22.6, 15.8, 15.7, 14.0, 10.9, 8.0, 3.8; δ_F -78.9; MS (ESI): Calcd for $C_{25}H_{46}N_3^+$ [M+H]$^+$: 388.368, found 388.360.

3-CP. T_g = 1.9 ºC; ^{1}H NMR (500 MHz, $CDCl_3$): δ_H 8.10 (s, 1H), 4.56-4.53 (t, *J* = 7.5 Hz, 2H), 4.29 (s, 3H), 2.05-2.02 (m, *J* = 7.5 Hz, 2H), 1.91-1.88 (m, 1H), 1.42-1.28 (m, 20H), 1.18-1.14 (m, 2H), 1.05-1.04 (dd, *J* = 5.0, 1.7 Hz, 2H), 0.93-0.90 (t, *J* = 7.0 Hz, 3H), 0.82-0.78 (m, 2H), 0.73-0.69 (td, *J* = 8.3, 5.2 Hz, 2H), 0.65-0.61 (dd, *J* = 8.3, 4.2 Hz, 2H), -0.22 – -0.28 (m, 4H). ^{13}C NMR (500 MHz, $CDCl_3$): δ_C 146.3, 127.7, 123.6, 121.1, 118.5, 115.9, 51.3, 40.1, 31.9, 30.1, 29.9, 29.5, 29.3, 28.9, 28.7, 28.6, 28.0, 27.9, 26.3, 22.7, 16.0, 15.9, 15.9, 15.7, 15.6, 14.1, 11.0, 10.8, 8.0, 3.8; δ_F -78.9; MS (ESI): Calcd for $C_{26}H_{46}N_3^+$ [M+H]$^+$: 400.368, found 400.350.

4-CP. T_g = 2.3 ºC; ^{1}H NMR (500 MHz, $CDCl_3$): δ_H 8.15 (s, 1H), 4.57-4.54 (t, *J* = 7.6 Hz, 2H), 4.31 (s, 3H), 2.6-2.03 (m, *J* = 7.5 Hz, 2H), 1.90-1.87 (m, 1H), 1.35-1.30 (m, 20H), 1.08-1.07 (d, *J* = 5.2 Hz, 2H), 1.03-1.00 (t, *J* = 7.5 Hz, 3H), 0.86-0.83 (m, 4H), 0.70-0.66 (m, 2H), 0.66-0.64 (dd, *J* = 8.4, 4.3 Hz, 2H), -0.23 – -0.24 (m, 6H); ^{13}C NMR (500 MHz, $CDCl_3$): δ_C 146.3, 127.9, 123.6, 121.1, 118.5, 115.9, 51.3, 40.2, 30.1, 29.5, 28.9, 28.7, 28.2, 28.1, 27.9, 26.3, 22.1, 17.9, 17.6, 16.3, 16.2, 16.1, 16.0, 15.9, 15.6, 14.5, 11.0, 10.8, 10.7, 8.0, 3.8; δ_F -78.9; MS (ESI): Calcd for $C_{27}H_{46}N_3^+$ [M+H]$^+$: 412.368, found 412.360.

Synthesis of IL ***3-AL***

Preparation of 1-methyl-1,2,3-triazole was adapted from the literature[2] with slight modifications, followed by the same procedure we used for **1-AL** and **2-AL** for *N*-substitution and metathesis.

3-AL. T_m = 49.2 ºC; ^{1}H NMR (500 MHz, $CDCl_3$): δ_H 8.50 (s, 1H), 8.45 (s, 1H), 4.60-4.57 (t, *J* = 7.5 Hz, 2H), 4.38 (s, 3H), 2.04-2.01 (m, *J* = 7.5 Hz, 2H), 1.36-1.27 (m, 30H), 0.91-0.89 (t, *J* = 7.0 Hz, 3H); ^{13}C NMR (500 MHz, $CDCl_3$): δ_C 131.7, 130.6, 123.6, 121.0, 118.4, 115.9, 54.3, 40.3, 31.9, 29.7, 29.7, 29.7, 29.7, 29.6, 29.6, 29.6, 29.5, 29.5, 29.5, 29.4, 29.2, 28.8, 26.1, 22.7, 14.1; ^{19}F NMR (300 MHz, $CDCl_3$): δ_F -79.0; MS (ESI): Calcd for $C_{21}H_{42}N_3^+$ [M+H]$^+$: 336.337, found 336.331.

Synthesis *of IL* ***4-AL***

The synthesis of **4-AL** was specifically designed for SCXRD studies and followed the procedure established for **1-AL** and **2-AL** with minor reagent modifications. 4-Cyclopropyl-1-methyl-1,2,3-triazole (1.2 equiv.) and 1-bromohexane (1.0 equiv.) were refluxed in acetonitrile for 72 hours. Following solvent removal, the resulting bromide salt was dissolved in deionized water (100 mL) and treated with aqueous sodium tetraphenylborate (1.1 equiv., 25 mL). The tetraphenylborate anion was selected to promote crystallinity through enhanced π-π stacking interactions and increased molecular rigidity compared to the $[NTf_2]^-$ analogs. After stirring for 3 hours at ambient temperature, the precipitated product was collected, washed with water and hexanes, and dried *in vacuo* to afford **4-AL** as a white solid in excellent yield. Single crystals suitable

for X-ray diffraction were obtained by slow evaporation of an CH_3CN (10 mg in 2 mL) in a capped NMR tube with a needle vent over six weeks.

Differential Scanning Calorimetry

Melting points and glass transition temperatures are reported as the transition from crystalline solid state to the isotropic liquid state, distinguished by the magnitude of enthalpy for the transition and the shape of the DSC curve. For each experiment, 5−15 mg of the samples were cooled to -90 °C, equilibrated for 5 min and then heated at a ramp rate of 5 °C/min to 200 °C. Three heating/cooling cycles were performed to ensure reproducibility, with the first cycle used to remove thermal history. Determined by the TRIOS analysis software, melting points are reported as the melting onset temperature and glass transition temperatures are reported as the midpoints of the phase transitions. Reported values are the average of three measurements. All measurements were carried out under a nitrogen atmosphere (50 mL/min) and were reproducible to within ±1 °C.

At the melting point, equilibrium exists between the solid and liquid phases and the change in Gibbs free energy is zero:

$$\Delta_{fus}G = 0 = \Delta_{fus}H - T_m\Delta_{fus}S \quad \Rightarrow \quad T_m = \frac{\Delta_{fus}H}{\Delta_{fus}S}$$

$\Delta_{fus}H$ is the enthalpy of fusion, T_m is the melting point, and $\Delta_{fus}S$ is the entropy of fusion. The T_m is determined as a delicate balance between the enthalpy and entropy of fusion. Note that decreases in the enthalpy and increases in entropy result in a melting point reduction.

Thermogravimetric Analysis

Thermogravimetric Analysis (TGA) was performed using a TA Instruments TGA 550. Samples of 5–15 mg were loaded onto the sample pan, and a preheated isotherm of 130 °C for 15 minutes followed by a cooling to 50 °C was utilized as the thermal history. Each sample underwent a temperature ramp of 10 °C/min to 600 °C under air. The *TRIOS* analysis software was used to determine a 5% mass loss over temperature to represent thermal decomposition.

Specific Heat Capacity Measurement

Specific heat capacity (C_p) was performed using a TA Discovery DSC 250 under nitrogen atmosphere (50 mL/min flow rate) with calibrations between each sample run. Samples of 5-15 mg were sealed in aluminum pans, and an empty pan was used as a reference. A reference pan and empty sample pan labeled “baseline run” followed a series of procedures: equilibrate to -50 °C with a 10-minute isotherm and ramp of 20 °C/min to 200 °C with a 10-minute isotherm.

Measurements followed a standard protocol of three heating/cooling cycles at 10°C/min across 25-200°C, with the first cycle eliminating thermal history. Three heating/cooling cycles were performed to ensure reproducibility. The specific heat capacity was calculated using the following equation:

$$C_p = (Q/m)/\Delta T$$

Where C_p is the specific heat capacity (J/g·K), Q is the heat flow (J), m is the sample mass (g), and ΔT is the temperature change (K).

Sapphire (α-Al_2O_3) was used as a calibration standard. A reference pan and sample pan containing a pre-weighed sapphire disk labeled "sapphire" followed the same procedure. The final run, a reference pan and sample pan containing sample labeled "sample run," followed the same procedure. TRIOS analysis software was used to transform the combination of each run into a figure displaying ASTM Heat Capacity *vs.* Temperature. C_p values were reported at 0 °C, 25 °C, 100 °C, and 150 °C in units of J g^{-1} K^{-1}.

Crystallographic Data and Discussion

Single crystal data of **1** and **4-AL** samples were collected on a goniometer of a Bruker D8 Quest diffractometer with Mo Kα wavelength (λ = 0.71073 Å) and a Photon II area detector. Examination and data collection were performed at 150 K. Data were collected, reflections were indexed and processed, and the files scaled and corrected for absorption using APEX4 or 5[3], SAINT and SADABS.[4]

For all compounds the space groups were assigned using XPREP within the SHELXTL suite of programs[5,6] and the structures were solved by dual methods using SHELXT[7] and refined by full matrix least squares against F^2 with all reflections using Shelxl2019[8] using the graphical interfaces Olex2[9] and/or ShelXle.[10] Carbon bound H atoms were positioned geometrically and constrained to ride on their parent atoms. C--H bond distances were constrained to 0.95 Å for aromatic and alkene C-H moieties, and to 1.00, 0.99 and 0.98 Å for aliphatic CH, CH_2 and CH_3 moieties, respectively. Methyl H atoms were allowed to rotate, but not to tip, to best fit the experimental electron density. U_{iso}(H) values were set to a multiple of U_{eq}(C) with 1.5 for CH_3, and 1.2 for C-H and CH_2.

The alkyl chain of **4-AL** is disordered over two conformations. The disorder extends to part of the triazole and the adjacent cyclopropane ring. The two disordered moieties were restrained to have similar geometries. U_{ij} components of ADPs for disordered atoms closer to each other than 2.0 Å were restrained to be similar. Subject to these conditions the occupancy ratio refined to 0.619(3) to 0.318(3). The methyl H atoms of C1 were refined as disordered (AFIX 127). The disorder ratio refined to 0.695(17) to 0.305(17).

Hirshfeld surfaces, the resultant images, energy frameworks, and fingerprint plots were calculated and produced using *CrystalExplorer21*.[11] Distance analysis of the structures was accomplished using *Olex2*[9] and *Mercury*.[12]

Complete crystallographic data, in CIF format, were deposited with the Cambridge Crystallographic Data Centre. CCDC numbers 2491537 and 2491538 contain the supplementary crystallographic data for this paper. These data can be obtained free of charge from The Cambridge Crystallographic Data Centre via www.ccdc.cam.ac.uk/data_request/cif.

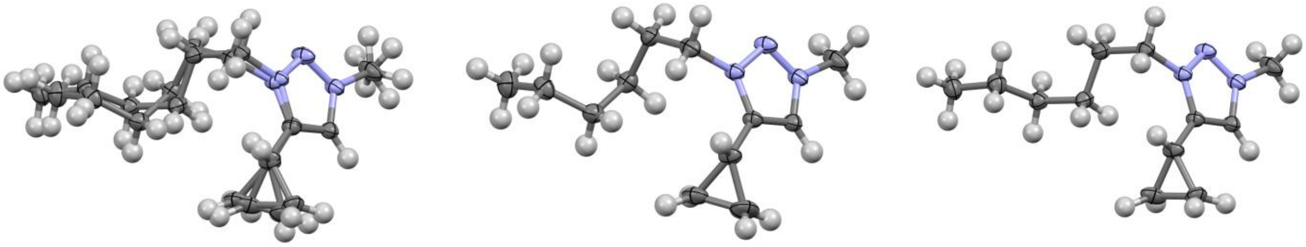

Figure S2. Depiction of the chain disorder in **4-AL**. The two disordered moieties are shown separately (left and middle) and the combined moiety at the right.

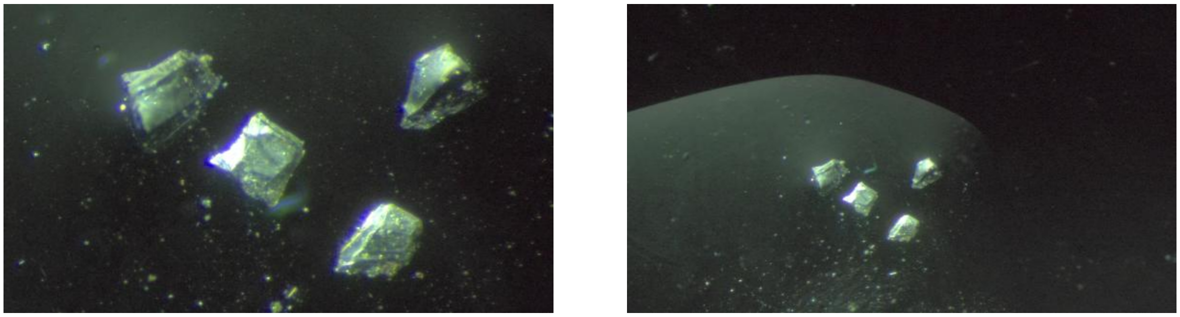

Figure S3. Images of the crystals of compound **1** viewed under a microscope.

Table S1. Crystal data and structure refinement information for compounds **4-AL** and **1.**

**Empirical formula**	$\mathbf{C_{36}H_{42}BN_3}$	$\mathbf{C_6H_9N_3}$
Formula weight	527.53	123.159
Temperature/K	150(2)	150.00
Crystal system	monoclinic	monoclinic
Space group	$P2_1/c$	$P2_1/c$
a/Å	17.3261(7)	10.6759(6)
b/Å	8.9838(4)	5.5233(3)
c/Å	20.9610(7)	11.1634(7)
α/°	90	90
β/°	111.7091(14)	92.689(3)
γ/°	90	90
Volume/Å^3	3031.3(2)	657.54(7)
Z	4	4
ρ_{calc}g/cm^3	1.156	1.244

μ/mm^{-1}	0.067	0.081
F(000)	1136.0	264.2
Crystal size/mm^3	0.42 × 0.18 × 0.12	0.55 × 0.13 × 0.11
Radiation	MoKα (λ = 0.71073)	Mo Kα (λ = 0.71073)
2Θ range for data collection/°	5.062 to 66.436	7.3 to 66.34
Index ranges	-26 ≤ h ≤ 26, -13 ≤ k ≤ 13, -32 ≤ l ≤ 30	-15 ≤ h ≤ 16, -8 ≤ k ≤ 8, -17 ≤ l ≤ 17
Reflections collected	96483	13504
Independent reflections	11593 [R_{int} = 0.0453, R_{sigma} = 0.0272]	2520 [R_{int} = 0.0396, R_{sigma} = 0.0292]
Data/restraints/parameters	11593/385/450	2520/0/83
Goodness-of-fit on F^2	1.025	1.028
Final R indexes [I>=2σ (I)]	R_1 = 0.0451, wR_2 = 0.1177	R_1 = 0.0324, wR_2 = 0.0693
Final R indexes [all data]	R_1 = 0.0663, wR_2 = 0.1318	R_1 = 0.0516, wR_2 = 0.0774
Largest diff. peak/hole / e Å^{-3}	0.36/-0.20	0.20/-0.32

Gas-Phase Ion Geometries and Energetics

Initial cation and anion structures were drawn in Ketcher[13] and subjected to preliminary 3D optimization using RDKit.[14] Given the considerable conformational flexibility of the ionic liquid cations, low-energy conformers were explored using the GOAT global optimization algorithm[15] implemented in ORCA 6.0[16] with the semi-empirical XTB method.[17] As expected, hundreds of conformers within 3 kcal/mol of the lowest-energy structure were identified for each cation. The global minimum conformer was then re-optimized in Gaussian 16[18] using DFT at the M062X/6-31+G(d,p) level of theory.

To choose a reliable method for calculating heats of combustion, we first benchmarked three common methods: B3LYP/6-31++G(d,p), G3MP2B3, and M06-2X/6-31+G(d,p), against a small set of benchmark fuels with well-established experimental VHOC values (Figure 2).[19] The comparison (Figure S4) showed a clear trend: M06-2X/6-31+G(d,p) consistently matched the experimental data most closely, especially for strained hydrocarbons where bond-strain release dominates the combustion energy. B3LYP tended to underestimate these values, and while G3MP2B3 performed reasonably well, it is simply not practical for molecules the size of our PCP-IL ion pairs. A single ion pair contains well over 150 atoms, and running a full G3 calculation at that scale is computationally prohibitive. In contrast, M06-2X provides good accuracy for strained systems while remaining feasible for the large systems studied here. For these reasons, all production-level optimizations and combustion-enthalpy calculations were carried out at the M06-2X/6-31+G(d,p) level.

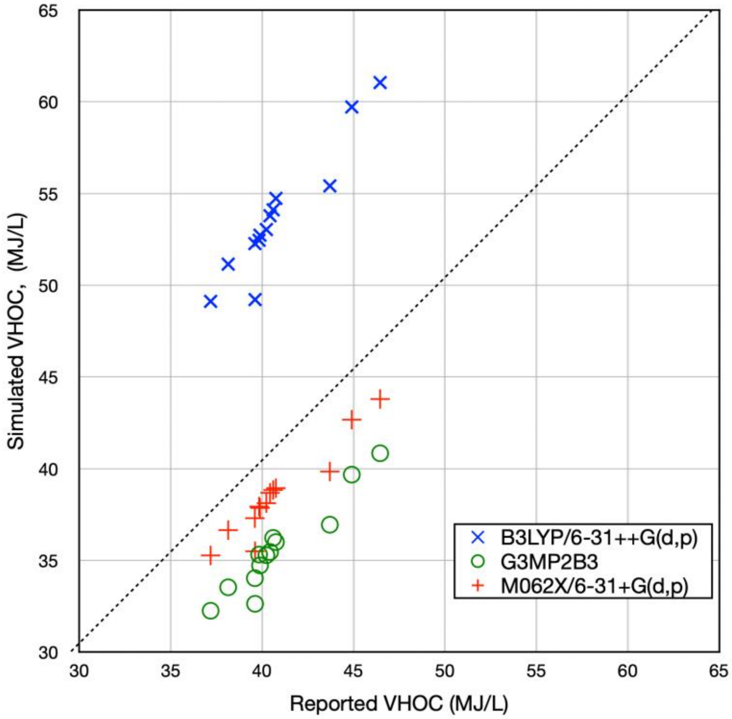

Figure S4. Parity plot comparing calculated and experimental volumetric heats of combustion (VHOC) for the benchmark fuel set using B3LYP/6-31++G(d,p), G3MP2B3, and M06-2X/6-31+G(d,p).

Gas-phase cation–anion pairs were constructed by packing the optimized anion geometry around the charged headgroup of the optimized cation using Packmol.[20] GOAT was again used to identify low-energy ion-pair conformers at the XTB level, and the global minimum structure was reoptimized with B3LYP/6-31++G(d,p) in Gaussian 16.

The pairing energy was estimated by subtracting the sum of the electronic and zero-point energies of the isolated cation and anion from that of the ion pair. To estimate the standard gas phase enthalpy of combustion, all relevant species (including O_2, CO_2, H_2O, HF, N_2, and SO_2) were optimized and analyzed using the same level of theory. The enthalpy change was approximated from the difference in the sum of electronic and thermal enthalpies between products and reactants, based on stoichiometric balancing of the combustion reaction.

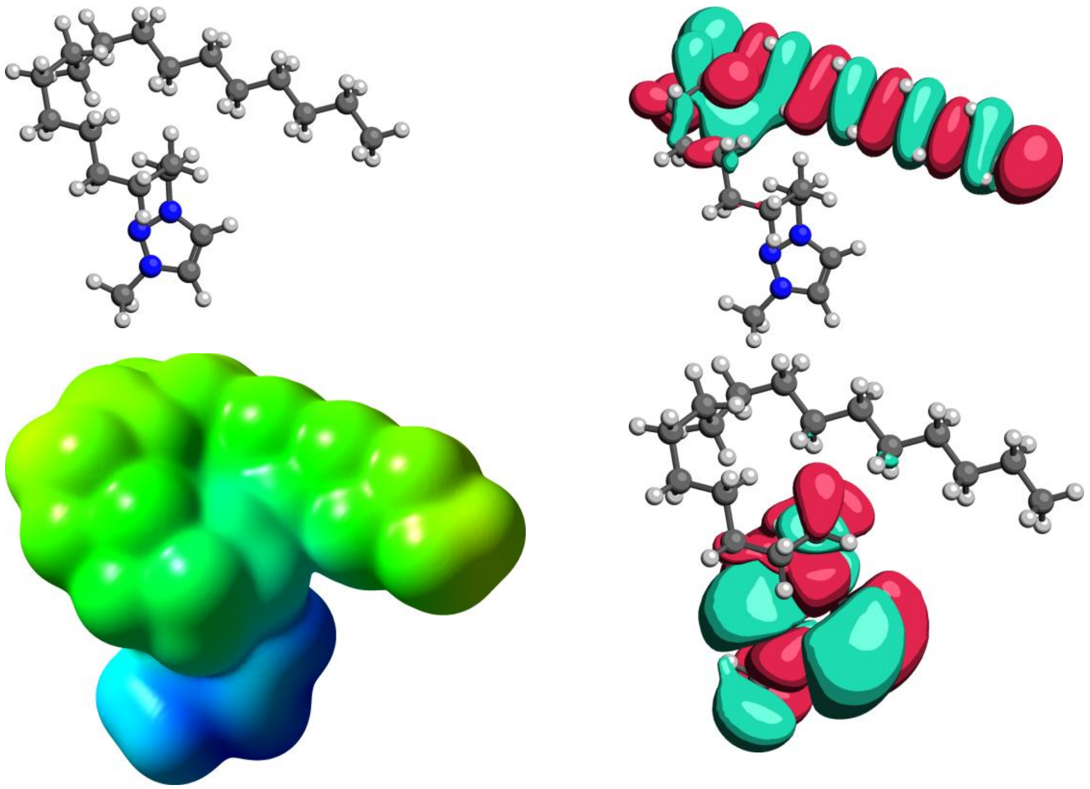

Figure S5. Optimized geometry and electronic properties of **3-AL** cation. Clockwise from top left: (1) ball-and-stick representation of the M062X/6-31+G(d,p) optimized geometry; (2) highest occupied molecular orbital (HOMO); (3) lowest unoccupied molecular orbital (LUMO); and (4) electrostatic potential (ESP) surface mapped onto the electron density, with color indicating relative charge density (blue = positive, green = neutral, red = negative) over the range 0 to +441 kJ/mol.

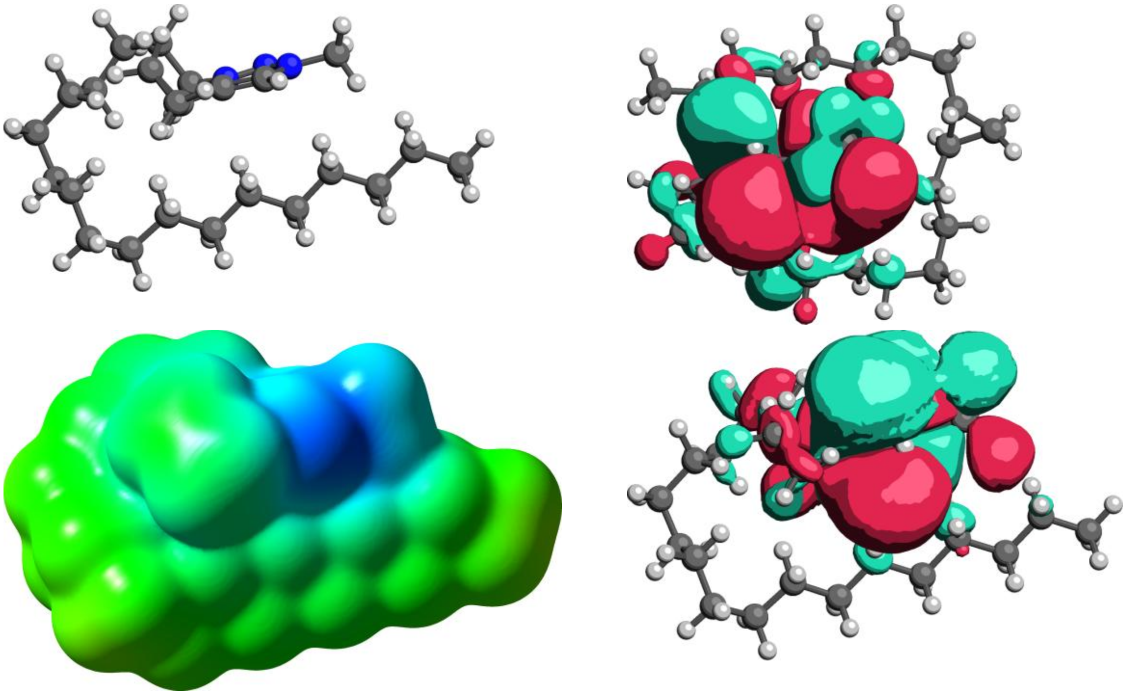

Figure S6. Optimized geometry and electronic properties of **2-AL** cation. Clockwise from top left: (1) ball-and-stick representation of the M062X/6-31+G(d,p) optimized geometry; (2) highest occupied molecular orbital (HOMO); (3) lowest unoccupied molecular orbital (LUMO); and (4) electrostatic potential (ESP) surface mapped onto the electron density, with color indicating relative charge density (blue = positive, green = neutral, red = negative) over the range 0 to +441 kJ/mol.

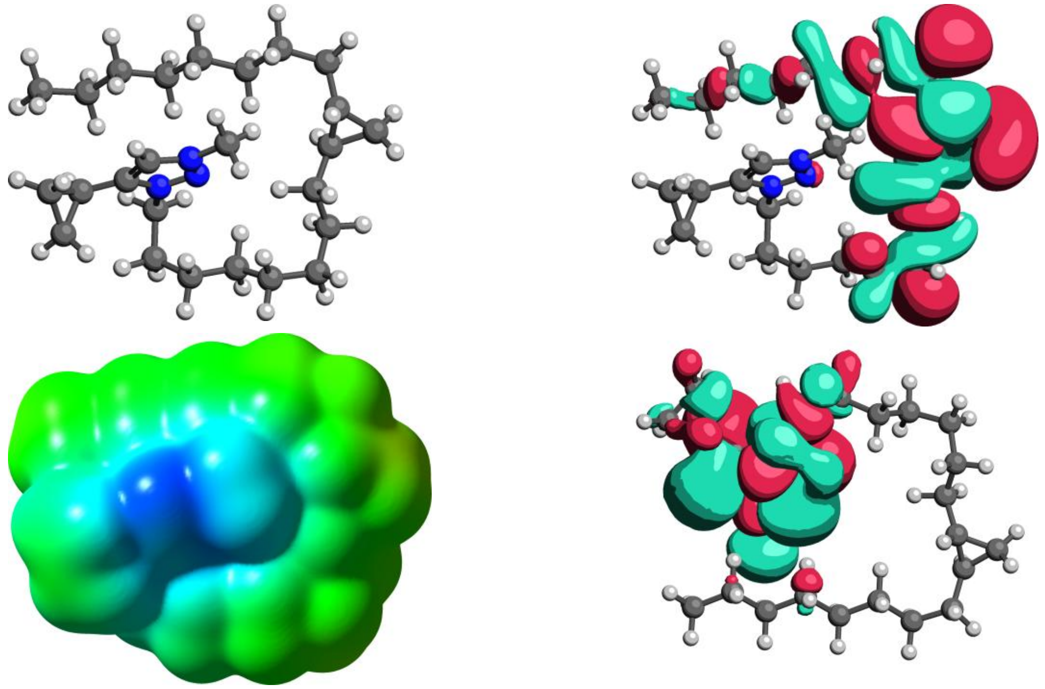

Figure S7. Optimized geometry and electronic properties of **2-CP** cation. Clockwise from top left: (1) ball-and-stick representation of the M062X/6-31+G(d,p) optimized geometry; (2) highest occupied molecular orbital (HOMO); (3) lowest unoccupied molecular orbital (LUMO); and (4) electrostatic potential (ESP) surface mapped onto the electron density, with color indicating relative charge density (blue = positive, green = neutral, red = negative) over the range 0 to +441 kJ/mol.

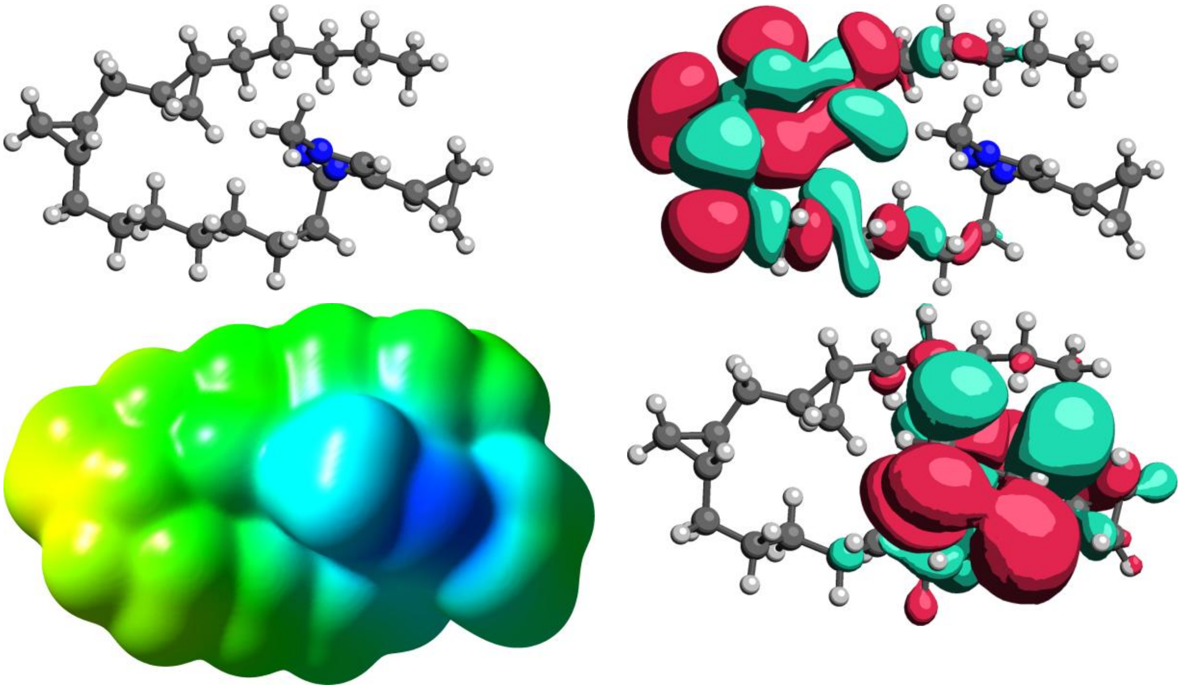

Figure S8. Optimized geometry and electronic properties of **3-CP** cation. Clockwise from top left: (1) ball-and-stick representation of the M062X/6-31+G(d,p) optimized geometry; (2) highest occupied molecular orbital (HOMO); (3) lowest unoccupied molecular orbital (LUMO); and (4) electrostatic potential (ESP) surface mapped onto the electron density, with color indicating relative charge density (blue = positive, green = neutral, red = negative) over the range 0 to +441 kJ/mol.

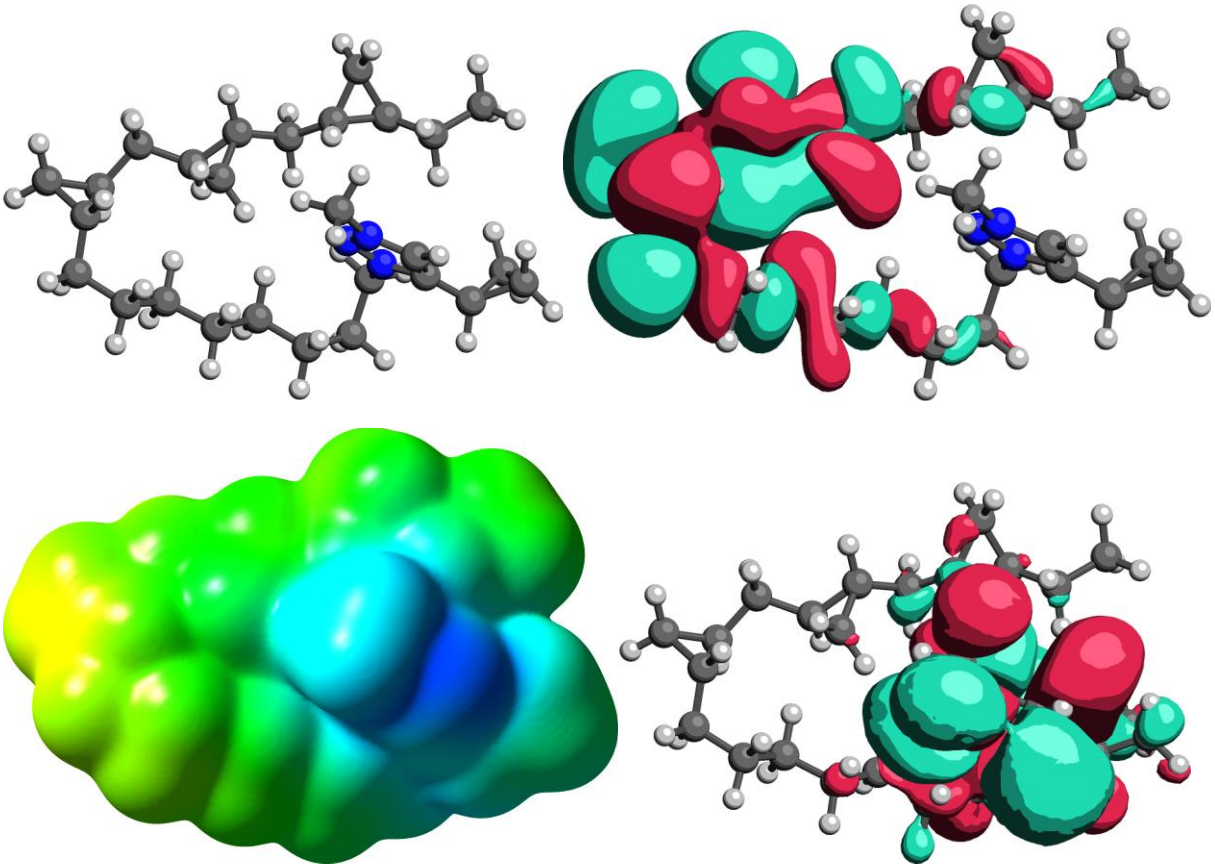

Figure S9. Optimized geometry and electronic properties of **4-CP** cation. Clockwise from top left: (1) ball-and-stick representation of the M062X/6-31+G(d,p) optimized geometry; (2) highest occupied molecular orbital (HOMO); (3) lowest unoccupied molecular orbital (LUMO); and (4) electrostatic potential (ESP) surface mapped onto the electron density, with color indicating relative charge density (blue = positive, green = neutral, red = negative) over the range 0 to +441 kJ/mol.

Table S1. Quantum chemical energies of isolated species computed at the M062X/6-31+G(d,p) level of theory. Energies are reported in Hartree. The number of conformers within 12.6 kJ/mol of the global minimum was determined using the XTB/GOAT conformer search implemented in ORCA.

Species	E_{elec} + ZPE	$E_{enthalpy}$	Conformers
**3-AL**	-988.514776	-988.485504	753
**2-AL**	-1105.127503	-1105.655559	406
**2-CP**	-1143.191702	-1143.159526	326
**3-CP**	-1181.252521	-1181.219594	226
**4-CP**	-1219.313922	-1219.280406	660
$[NTf_2]^-$	-1826.808885	-1826.793765	4
O_2	-150.259899	-150.256593	1
CO_2	-188.504166	-188.500604	1
H_2O	-76.373367	-76.369587	1
HF	-100.401044	-100.438814	1
N_2	-109.524188	-109.482945	1
SO_2	-548.490037	-548.486029	1

Table S2. Quantum chemical energies of gas-phase ion pairs computed at the M062X/6-31+G(d,p) level of theory. Energies are reported in Hartree. The number of conformers within 12.6 kJ/mol of the global minimum was determined using the XTB/GOAT conformer search implemented in ORCA.

Species	E_{elec} + ZPE	$E_{enthalpy}$	Conformers
**3-AL**	-2815.460913	-2815.415821	149
**2-AL**	-2932.069009	-2932.021927	70
**2-CP**	-2970.132101	-2970.084251	39
**3-CP**	-3008.191847	-3008.143393	276
**4-CP**	-3046.258564	-3046.210245	193

Combustion stoichiometries:

3-AL + $32O_2 \rightarrow 23CO_2 + 18H_2O + 6HF + 2N_2 + 2SO_2$ (1)

2-AL + $36O_2 \rightarrow 26CO_2 + 20H_2O + 6HF + 2N_2 + 2SO_2$ (2)

2-CP + $37O_2 \rightarrow 27CO_2 + 20H_2O + 6HF + 2N_2 + 2SO_2$ (3)

3-CP + $38O_2 \rightarrow 28CO_2 + 20H_2O + 6HF + 2N_2 + 2SO_2$ (4)

4-CP + $39O_2 \rightarrow 29CO_2 + 20H_2O + 6HF + 2N_2 + 2SO_2$ (5)

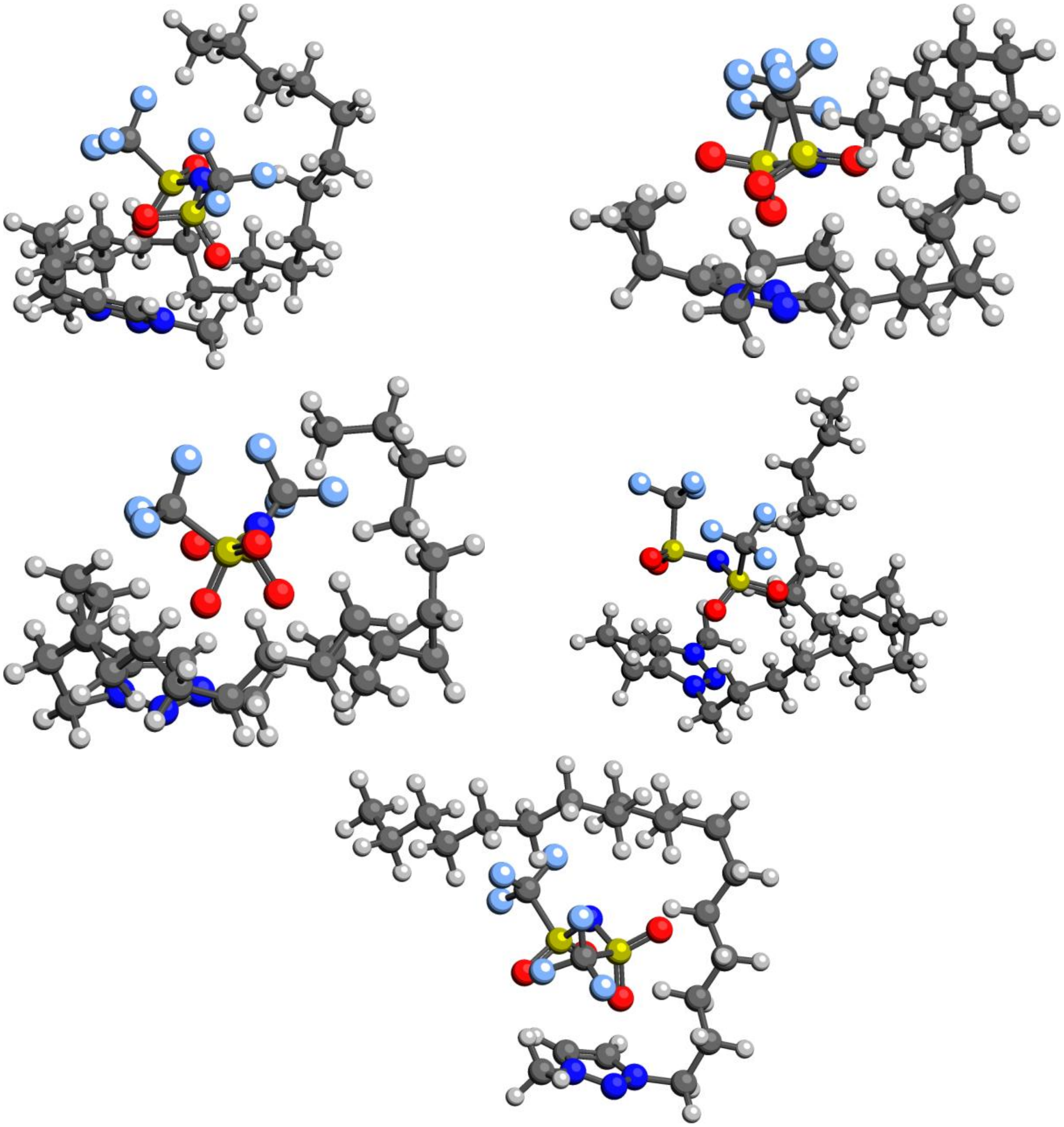

Figure S10. Optimized gas-phase geometries of IL ion pairs. Shown clockwise from top left are **2-AL, 2-CP, 3-CP**, **4-CP**, and **3-AL** each optimized at the M062X/6-31+G(d,p) level of theory.

References

(1) O'Brien, R. A.; Hillesheim, P. C.; Soltani, M.; Badilla-Nunez, K. J.; Siu, B.; Musozoda, M.; West, K. N.; Davis, J. H. Jr.; Mirjafari, A. Cyclopropane as an Unsaturation "Effect Isostere": Lowering the Melting Points in Lipid-like Ionic Liquids. *J. Phys. Chem. B* **2023**, *127* (6), 1429–1442.

(2) Fletcher, J. T.; Walz, S. E.; Keeney, M. E. Monosubstituted 1,2,3-Triazoles from Two-Step One-Pot Deprotection/Click Additions of Trimethylsilylacetylene. *Tetrahedron Letters* **2008**, *49* (49), 7030–7032.

(3) Apex4 v2022.10-1, Apex5 v2023.9-2, SAINT V8.40B, 2022/2023.

(4) Krause, L.; Herbst-Irmer, R.; Sheldrick, G. M.; Stalke, D. Comparison of Silver and Molybdenum Microfocus X-Ray Sources for Single-Crystal Structure Determination. *J. Appl. Crystallogr.* **2015**, *48* (1), 3–10.

(5) SHELXTL Suite of Programs, Version 6.14, 2000-2003, Bruker Advanced X-Ray Solutions.

(6) Sheldrick, G. M. A Short History of *SHELX*. *Acta Crystallographica Section A Foundations of Crystallography* **2008**, *64* (1), 112–122.

(7) Sheldrick, G. M. *SHELXT* – Integrated Space-Group and Crystal-Structure Determination. *Acta Crystallographica Section A Foundations and Advances* **2015**, *71* (1), 3–8. https://doi.org/10.1107/S2053273314026370.

(8) Sheldrick, G. M. Crystal Structure Refinement with *SHELXL*. *Acta Crystallographica Section C Structural Chemistry* **2015**, *71* (1), 3–8. https://doi.org/10.1107/S2053229614024218.

(9) Dolomanov, O. V.; Bourhis, L. J.; Gildea, R. J.; Howard, J. A. K.; Puschmann, H. *OLEX2* : A Complete Structure Solution, Refinement and Analysis Program. *J. Appl. Crystallogr.*, *42* (2), 339–341. https://doi.org/10.1107/S0021889808042726.

(10) Hübschle, C. B.; Sheldrick, G. M.; Dittrich, B. *ShelXle* : A Qt Graphical User Interface for *SHELXL*. *J. Appl. Crystallogr.* **2011**, *44* (6), 1281–1284.

(11) Spackman, P. R.; Turner, M. J.; McKinnon, J. J.; Wolff, S. K.; Grimwood, D. J.; Jayatilaka, D.; Spackman, M. A. *CrystalExplorer* : A Program for Hirshfeld Surface Analysis, Visualization and Quantitative Analysis of Molecular Crystals. *J. Appl. Crystallogr.* **2021**, *54* (3), 1006–1011.

(12) Macrae, C. F.; Bruno, I. J.; Chisholm, J. A.; Edgington, P. R.; McCabe, P.; Pidcock, E.; Rodriguez-Monge, L.; Taylor, R.; van de Streek, J.; Wood, P. A. *Mercury CSD 2.0* – New Features for the Visualization and Investigation of Crystal Structures. *J. Appl. Crystallogr.* **2008**, *41* (2), 466–470.

(13) Karulin, B.; Kozhevnikov, M. Ketcher: Web-Based Chemical Structure Editor. *J. Cheminf.* **2011**, *3*, P3.

(14) RDKit Development Team. RDKit: Open-Source Cheminformatics. http://www.rdkit.org (accessed September 24, 2025).

(15) de Souza, B. GOAT: A Global Optimization Algorithm for Molecules and Atomic Clusters. *Angew. Chem., Int. Ed.* **2025**, *64,* e202500393

(16) Neese, F. The ORCA Program System, Version 5.0. *WIREs Comput. Mol. Sci.* **2022**, *12* (5), e1606.

(17) Bannwarth, C.; Ehlert, S.; Grimme, S. GFN2-xTB—An Accurate and Broadly Parametrized Self-Consistent Tight-Binding Quantum Chemical Method with Multipole Electrostatics and

Density-Dependent Dispersion Contributions. *J. Chem. Theory Comput.* **2019**, *15* (3), 1652–1671.

(18) Gaussian 16, Revision C.01, Frisch, M. J.; Trucks, G. W.; Schlegel, H. B.; Scuseria, G. E.; Robb, M. A.; Cheeseman, J. R.; Scalmani, G.; Barone, V.; Petersson, G. A.; Nakatsuji, H.; Li, X.; Caricato, M.; Marenich, A. V.; Bloino, J.; Janesko, B. G.; Gomperts, R.; Mennucci, B.; Hratchian, H. P.; Ortiz, J. V.; Izmaylov, A. F.; Sonnenberg, J. L.; Williams-Young, D.; Ding, F.; Lipparini, F.; Egidi, F.; Goings, J.; Peng, B.; Petrone, A.; Henderson, T.; Ranasinghe, D.; Zakrzewski, V. G.; Gao, J.; Rega, N.; Zheng, G.; Liang, W.; Hada, M.; Ehara, M.; Toyota, K.; Fukuda, R.; Hasegawa, J.; Ishida, M.; Nakajima, T.; Honda, Y.; Kitao, O.; Nakai, H.; Vreven, T.; Throssell, K.; Montgomery, J. A., Jr.; Peralta, J. E.; Ogliaro, F.; Bearpark, M. J.; Heyd, J. J.; Brothers, E. N.; Kudin, K. N.; Staroverov, V. N.; Keith, T. A.; Kobayashi, R.; Normand, J.; Raghavachari, K.; Rendell, A. P.; Burant, J. C.; Iyengar, S. S.; Tomasi, J.; Cossi, M.; Millam, J. M.; Klene, M.; Adamo, C.; Cammi, R.; Ochterski, J. W.; Martin, R. L.; Morokuma, K.; Farkas, O.; Foresman, J. B.; Fox, D. J. Gaussian, Inc., Wallingford CT, 2016.

(19) Chowdhury, D.; Latchipatula, B. R.; Flaxer, E.; Yakovchuk, A.; Das, J.; Zevenbergen, J. F.; Gozin, M. Cyclopropyl-Rich Amines as High Energy Density Fuels for Advanced Propulsion. *ChemRxiv*, **2025**, https://doi.org/10.26434/chemrxiv-2025-mr52d.

(20) Martínez, L.; Andrade, R.; Birgin, E. G.; Martínez, J. M. PACKMOL: A Package for Building Initial Configurations for Molecular Dynamics Simulations. *J. Comput. Chem.* **2009**, *30* (13), 2157–2164.

^{1}H, ^{13}C and ^{19}F NMR Spectra of the Products

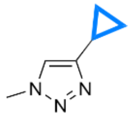

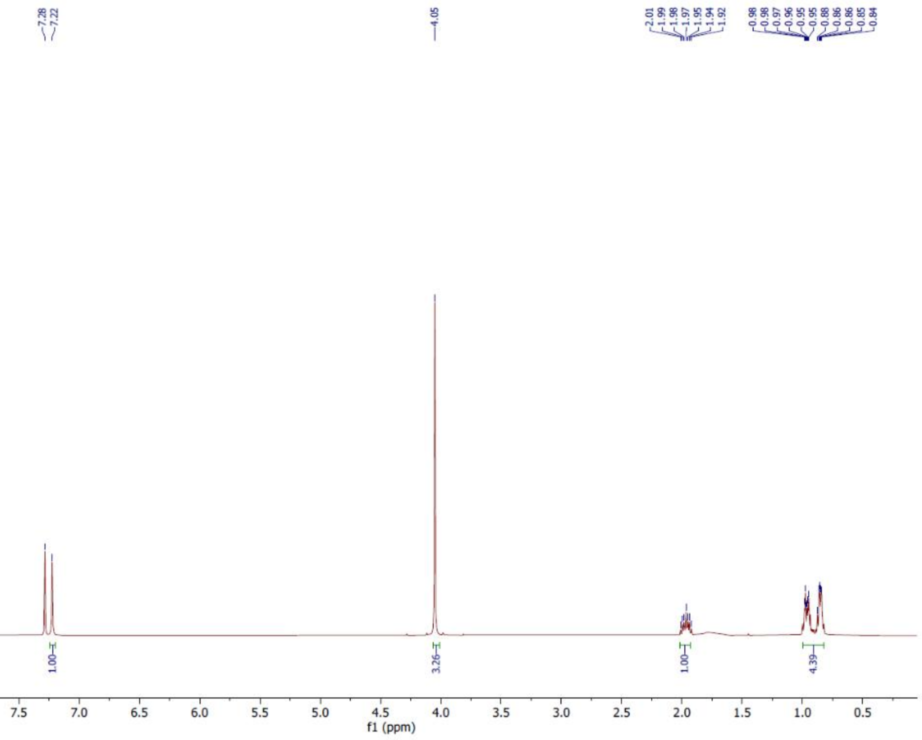

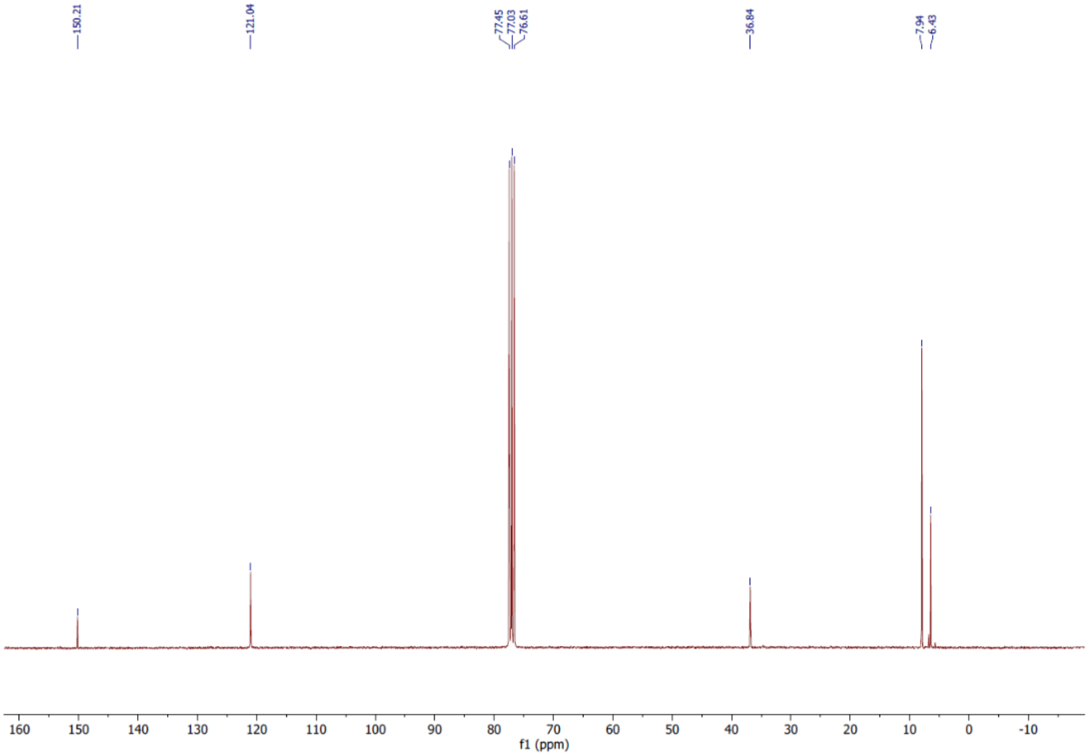

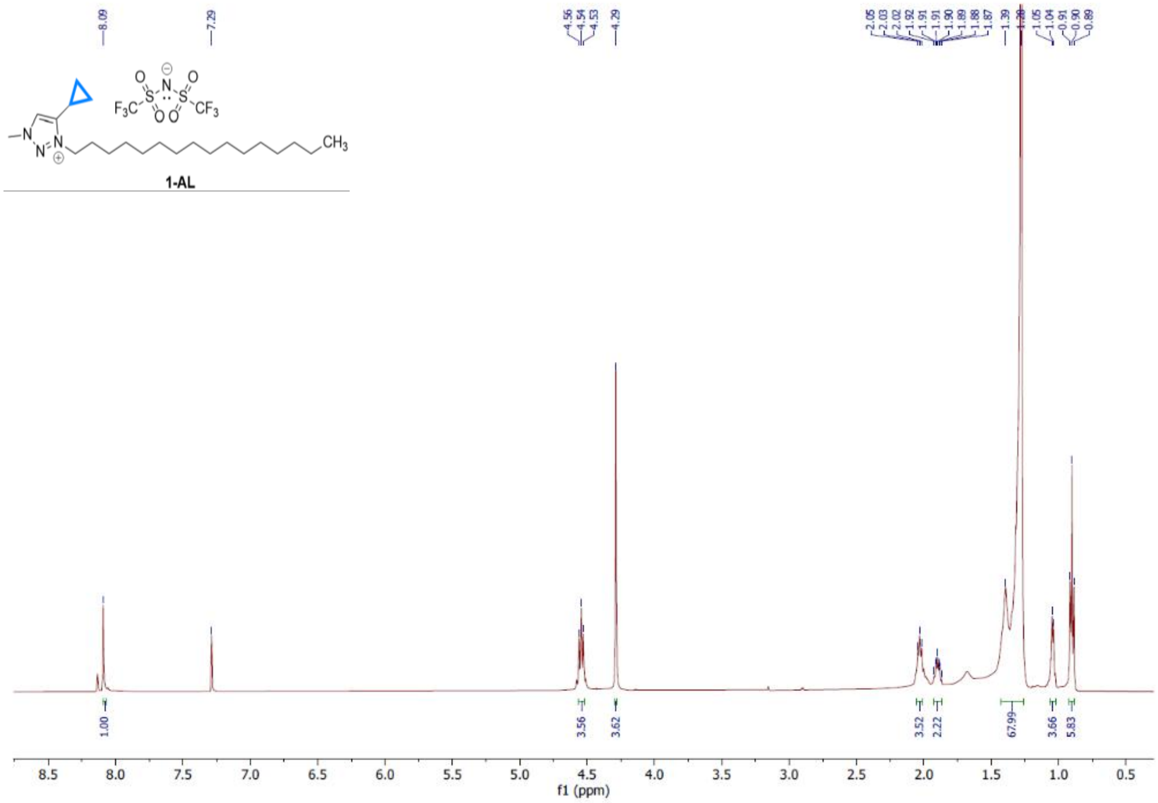

1-AL

8.09
7.29
4.56
4.54
4.53
4.29
2.05
2.03
2.02
1.92
1.91
1.91
1.90
1.89
1.88
1.87
1.39
1.05
1.04
0.91
0.90
0.89
1.00
3.56
3.62
3.52
2.22
67.99
3.66
5.83
8.5
8.0
7.5
7.0
6.5
6.0
5.5
5.0
4.5
4.0
3.5
3.0
2.5
2.0
1.5
1.0
0.5
f1 (ppm)

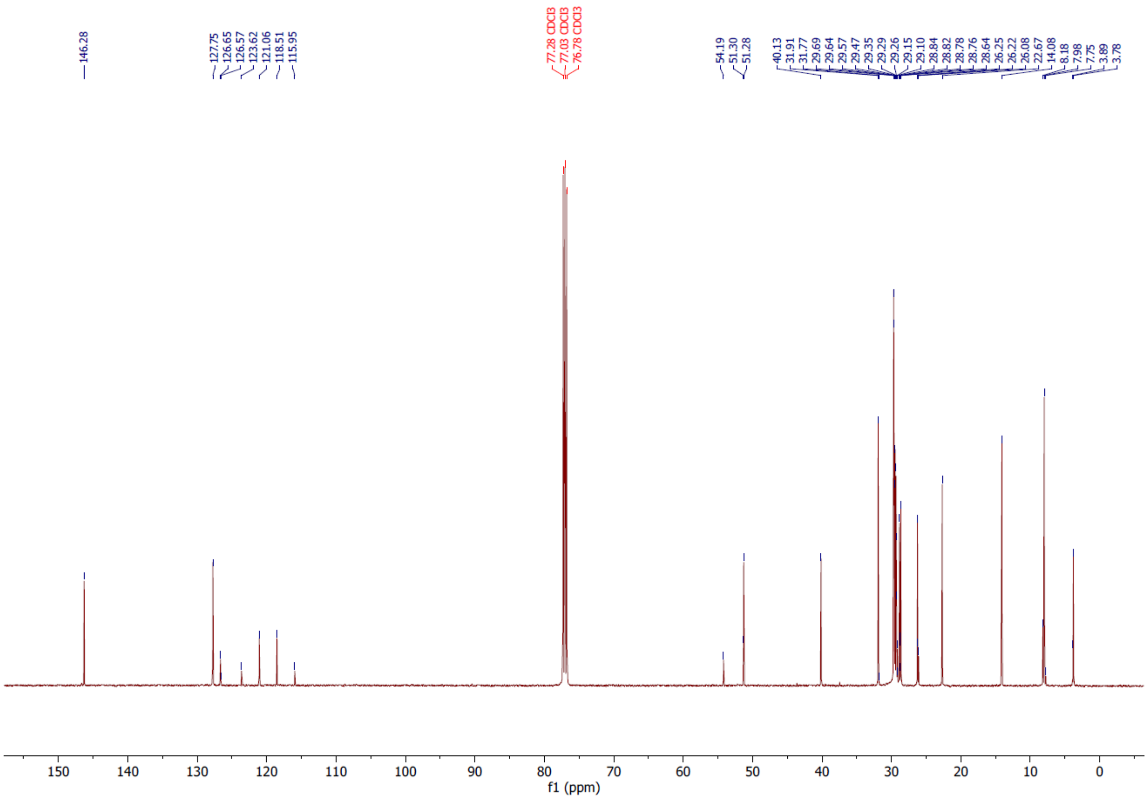

146.28
127.75
126.65
126.57
123.62
121.06
118.51
115.95
77.28 CDCl3
77.03 CDCl3
76.78 CDCl3
54.19
51.30
51.28
40.13
31.91
31.77
29.69
29.64
29.57
29.47
29.35
29.29
29.26
29.15
29.10
28.84
28.82
28.78
28.76
28.64
26.25
26.22
26.08
22.67
14.08
8.18
7.98
7.75
3.89
3.78
150
140
130
120
110
100
90
80
70
60
50
40
30
20
10
0
f1 (ppm)

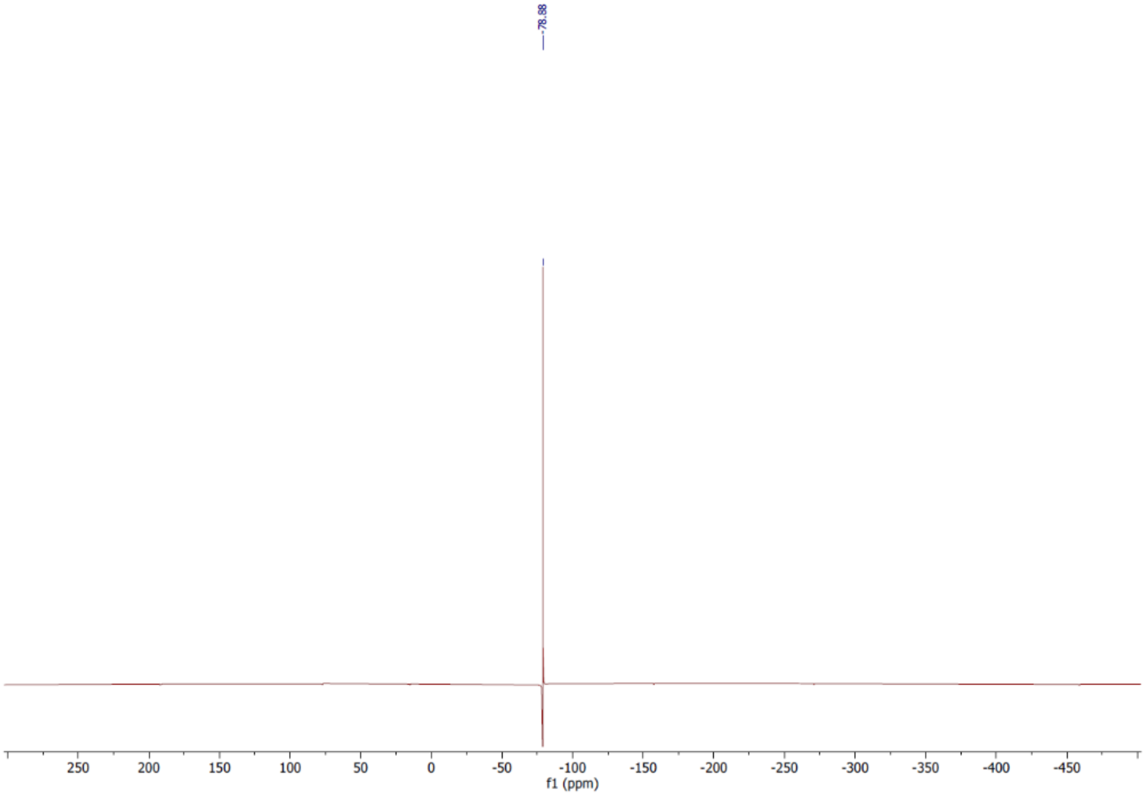

-78.88
250 200 150 100 50 0 -50 -100 -150 -200 -250 -300 -350 -400 -450
f1 (ppm)

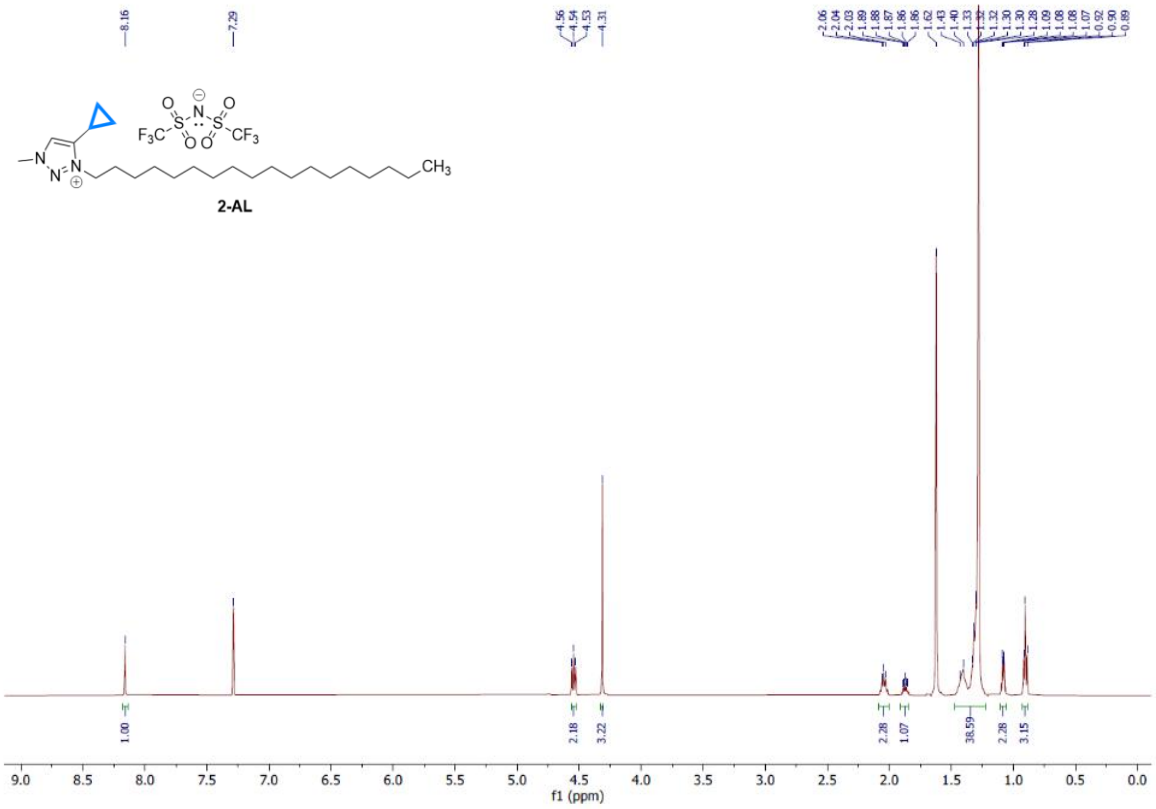

2-AL

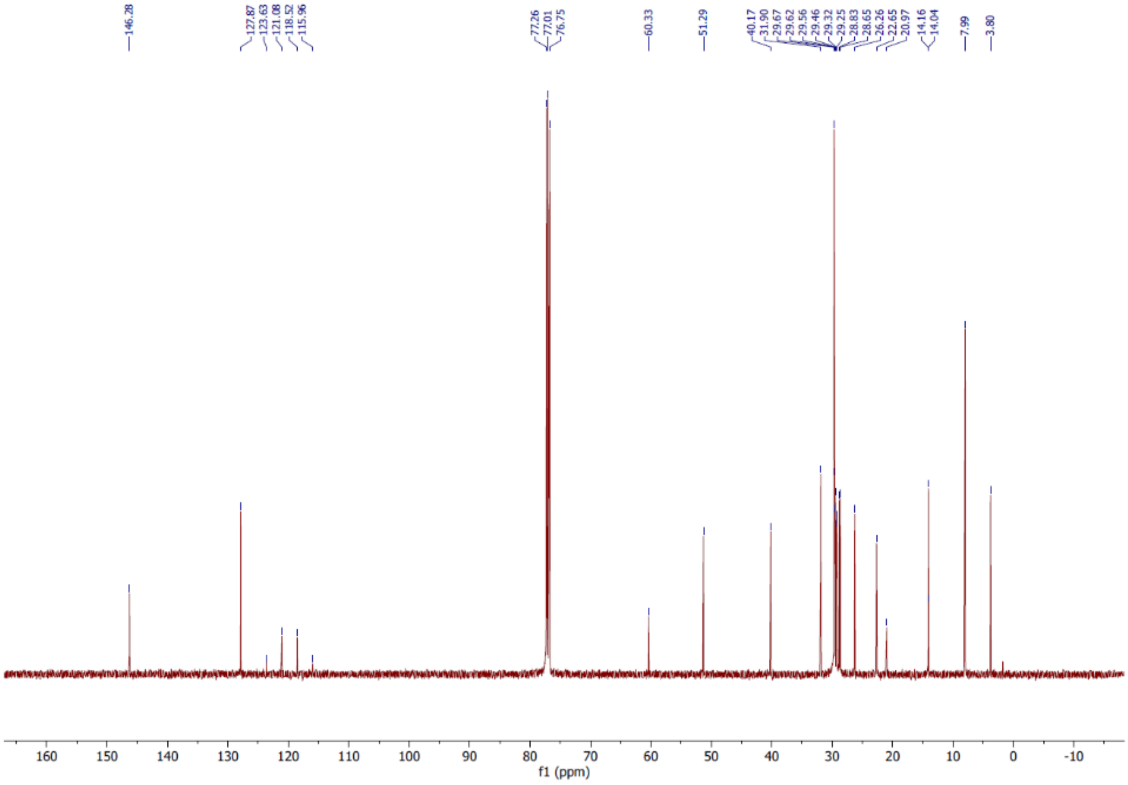

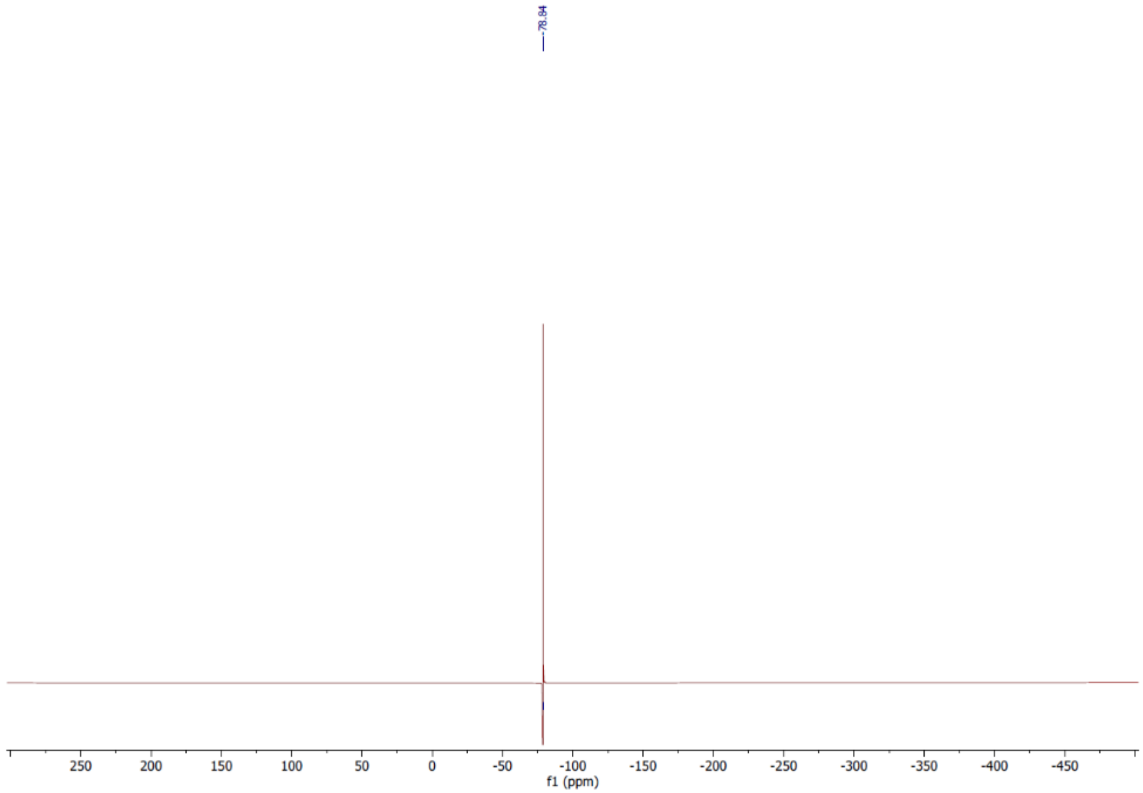

-78.84
250
200
150
100
50
0
-50
-100
-150
-200
-250
-300
-350
-400
-450
f1 (ppm)

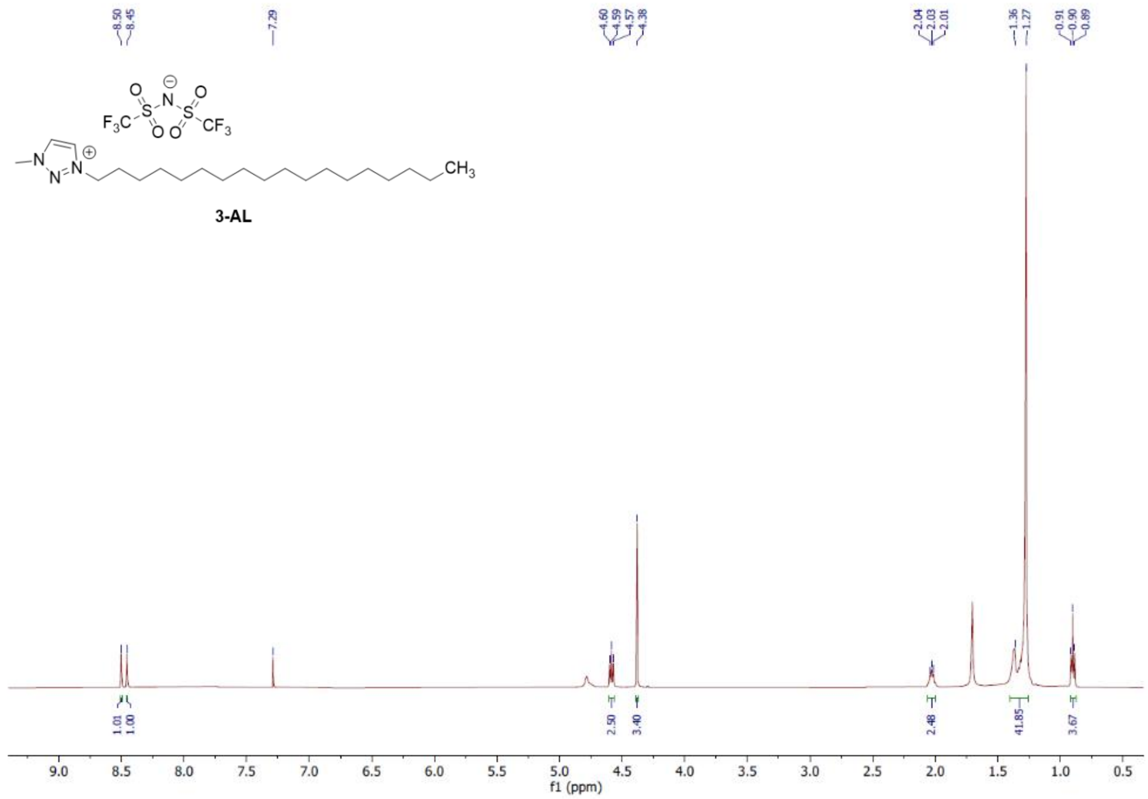
3-AL
f1 (ppm)

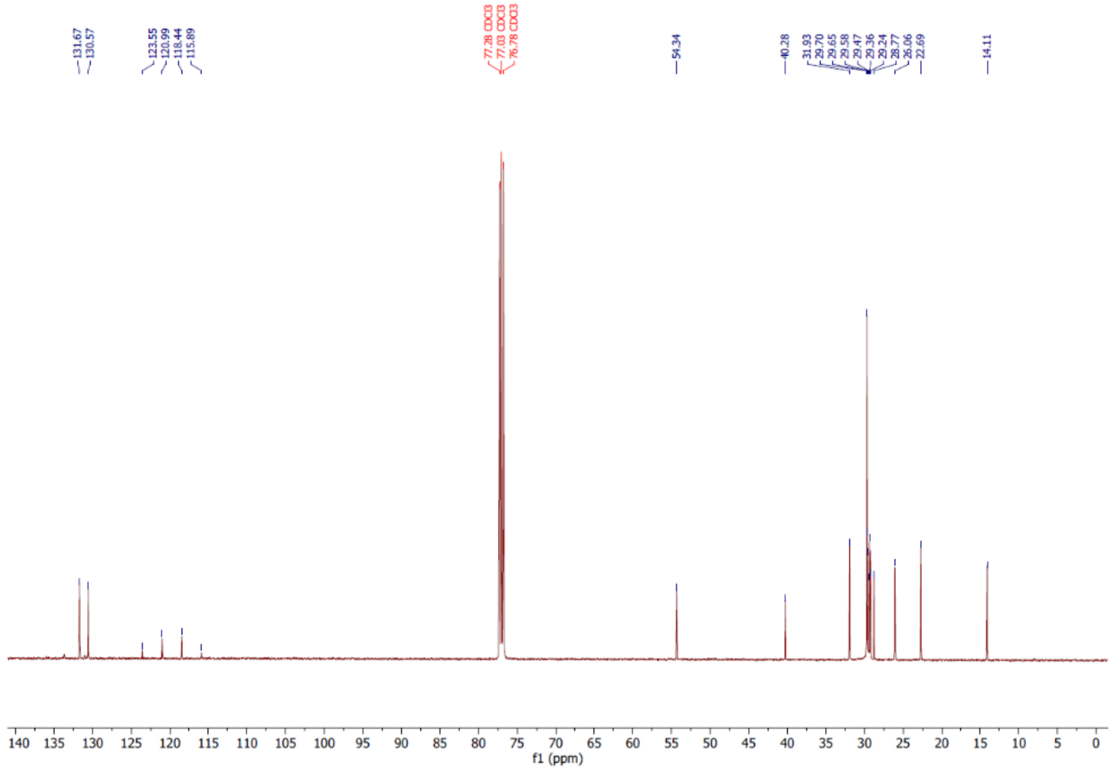
f1 (ppm)

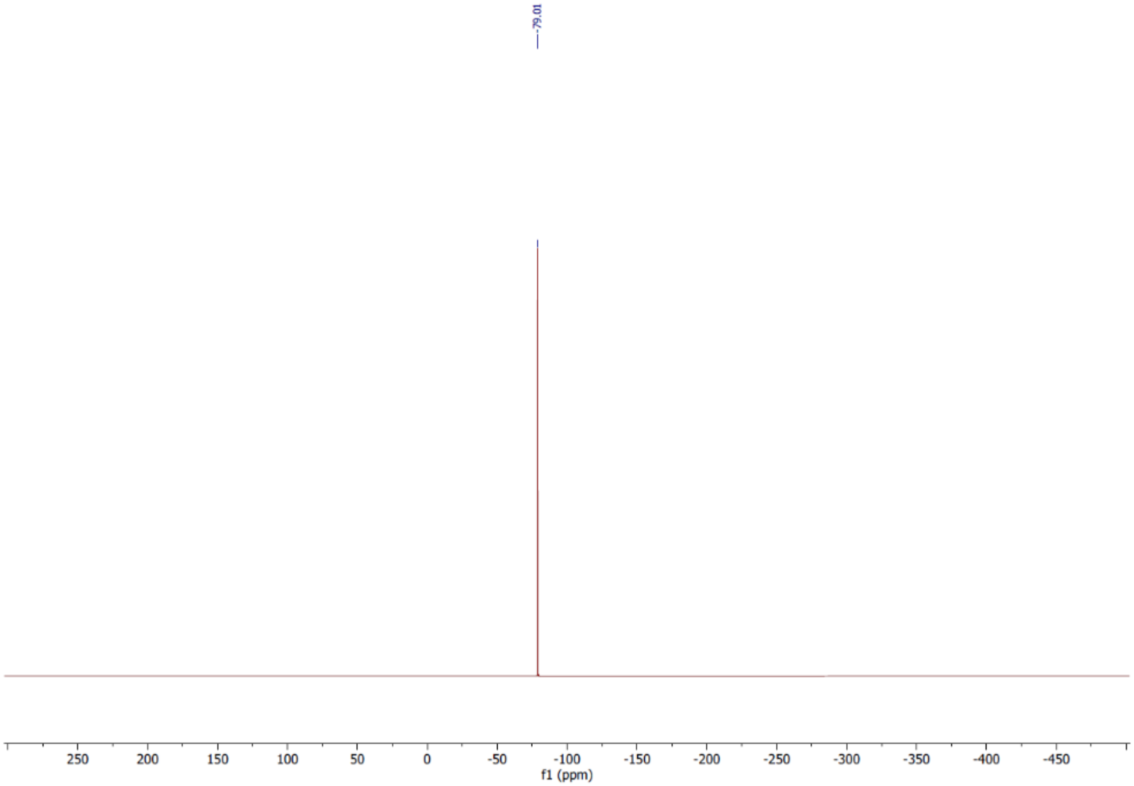
-79.01
250
200
150
100
50
0
-50
-100
-150
-200
-250
-300
-350
-400
-450
f1 (ppm)

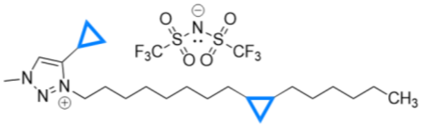
F3C
CF3
CH3

1-CP

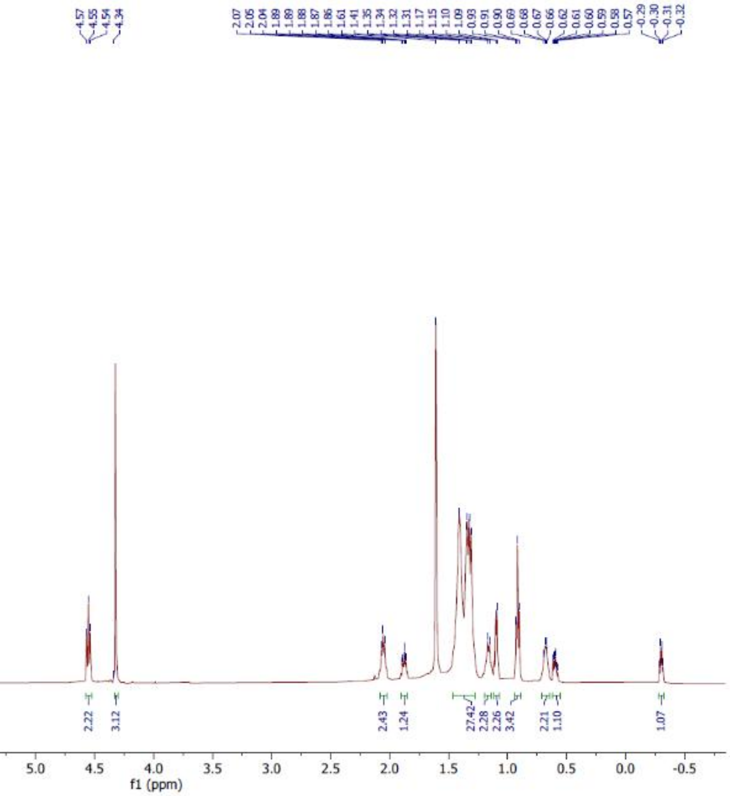
4.57 4.55 4.54 4.34
2.07 2.05 2.04 1.89 1.89 1.88 1.87 1.86 1.61 1.41 1.35 1.34 1.32 1.31 1.17 1.15 1.10 1.09 0.93 0.91 0.90 0.69 0.68 0.67 0.66 0.62 0.61 0.60 0.59 0.58 0.57 -0.29 -0.30 -0.31 -0.32
1.00 2.22 3.12 2.43 1.24 27.42 2.28 2.26 3.42 2.21 1.10 1.07
f1 (ppm)

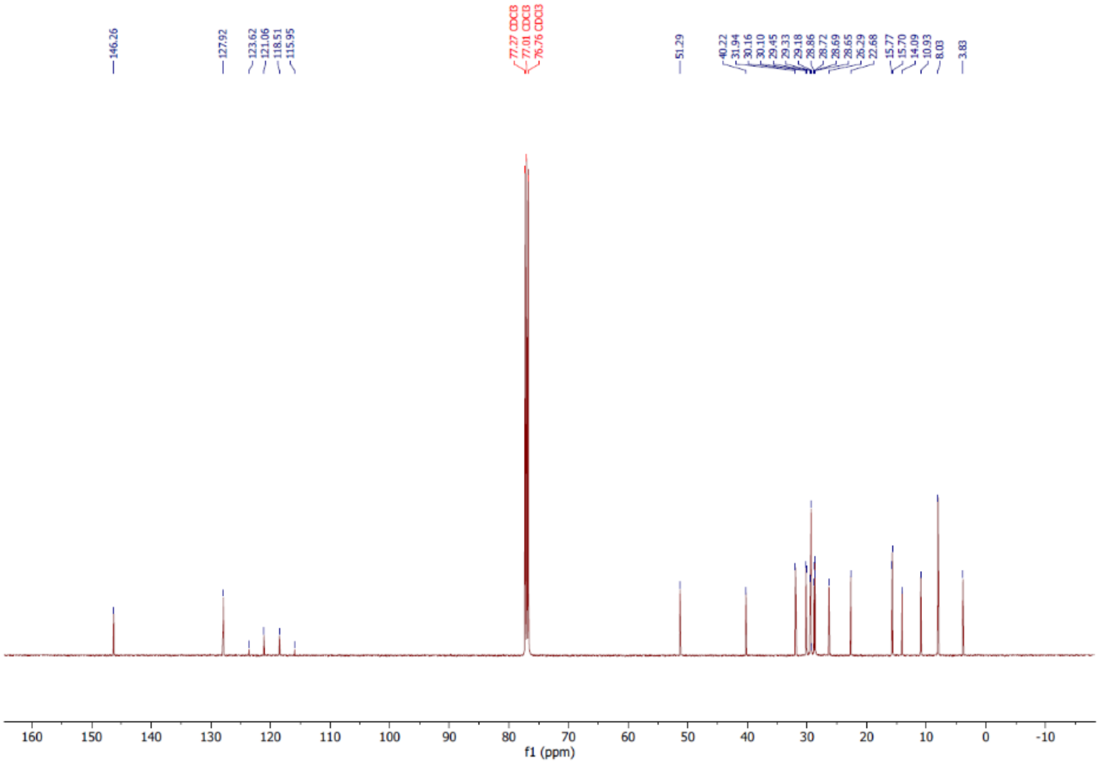
146.26 127.92 123.62 121.06 118.51 115.95
77.27 CDCl3 77.01 CDCl3 76.76 CDCl3
51.29 40.22 31.94 30.16 30.10 29.45 29.33 29.18 28.86 28.72 28.69 28.65 26.29 22.68 15.77 15.70 14.09 10.93 8.03 3.83
f1 (ppm)

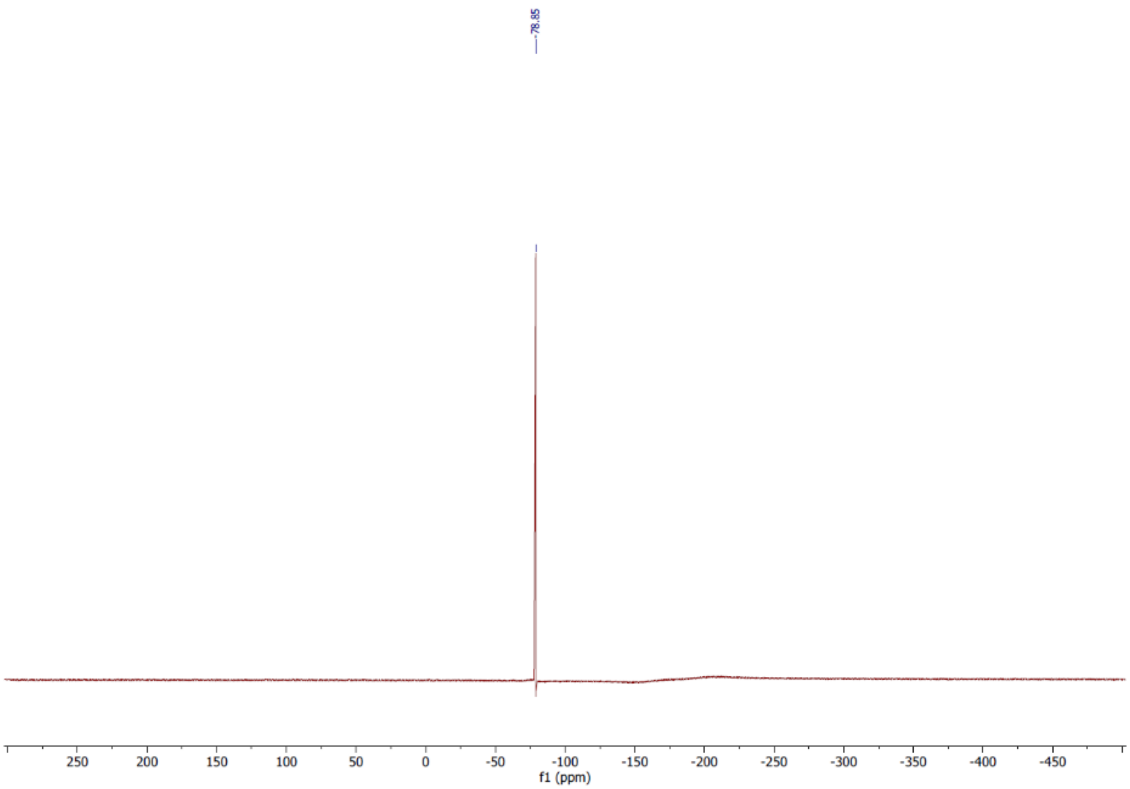
-78.85
250
200
150
100
50
0
-50
-100
-150
-200
-250
-300
-350
-400
-450
f1 (ppm)

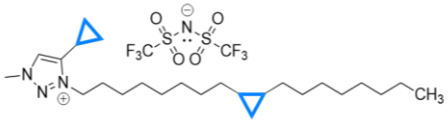

2-CP

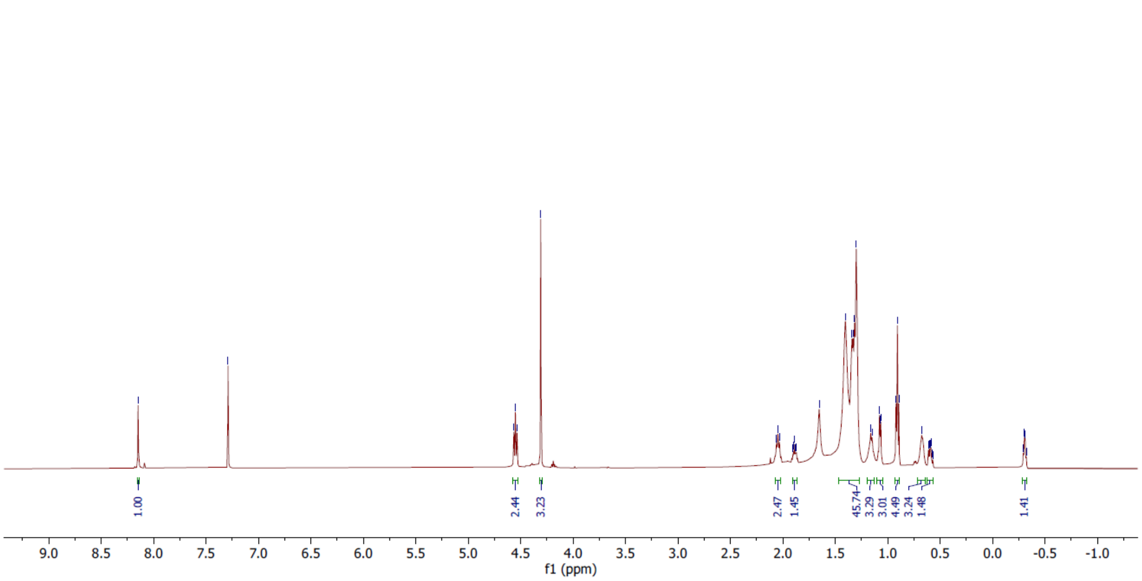
8.15
7.29
4.56 4.55 4.53 4.31
2.06 2.05 2.03 1.90 1.90 1.89 1.88 1.87 1.65 1.40 1.34 1.33 1.32 1.30 1.16 1.15 1.08 1.07 1.06 0.92 0.91 0.89 0.68 0.61 0.60 0.59 0.59 0.58 0.57
-0.29 -0.30 -0.31 -0.32
1.00
2.44
3.23
2.47
1.45
45.74
3.29
3.01
4.49
3.24
1.48
1.41
9.0 8.5 8.0 7.5 7.0 6.5 6.0 5.5 5.0 4.5 4.0 3.5 3.0 2.5 2.0 1.5 1.0 0.5 0.0 -0.5 -1.0
f1 (ppm)

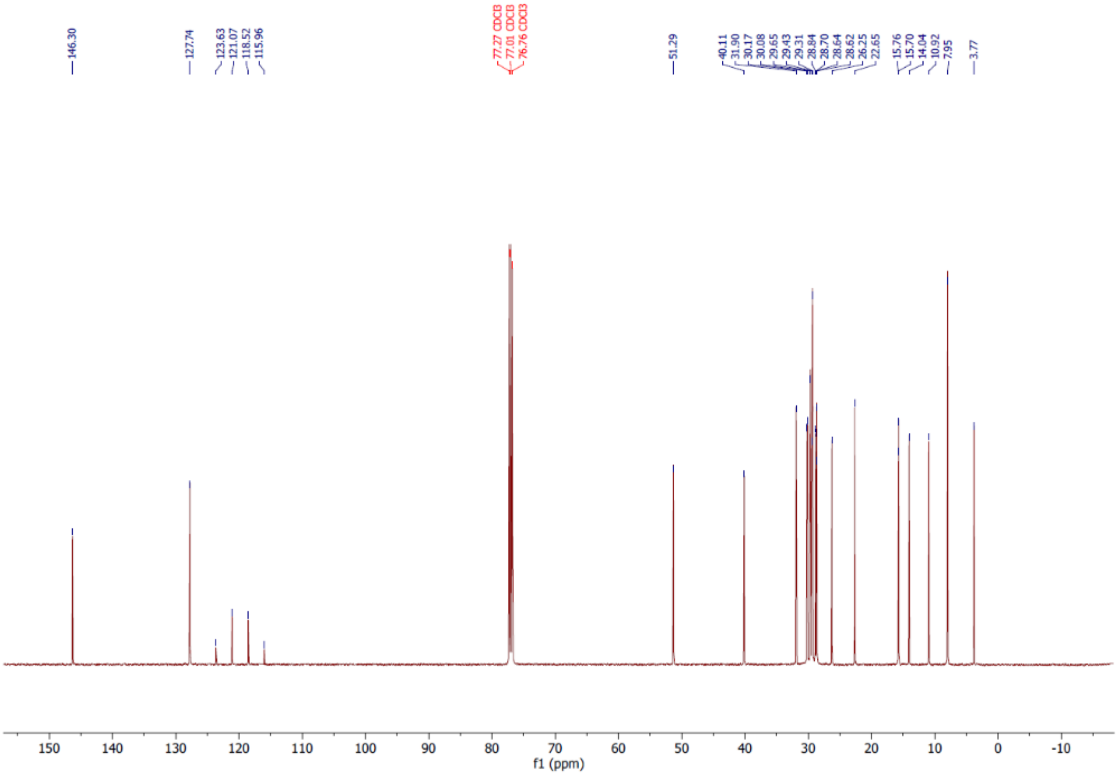
146.30
127.74 123.63 121.07 118.52 115.96
77.27 CDCl3
77.01 CDCl3
76.76 CDCl3
51.29
40.11 31.90 30.17 30.08 29.65 29.43 29.31 28.84 28.70 28.64 28.62 26.25 22.65
15.76 15.70 14.04 10.92 7.95
3.77
150 140 130 120 110 100 90 80 70 60 50 40 30 20 10 0 -10
f1 (ppm)

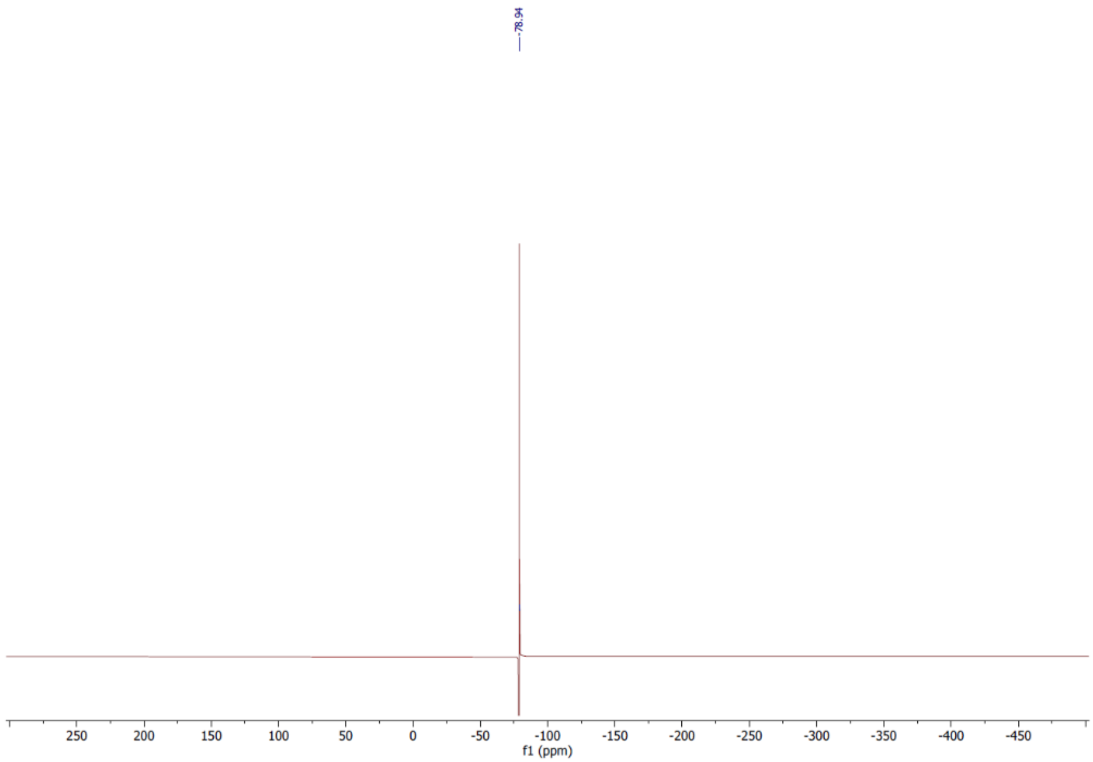

-78.94
250
200
150
100
50
0
-50
-100
-150
-200
-250
-300
-350
-400
-450
f1 (ppm)

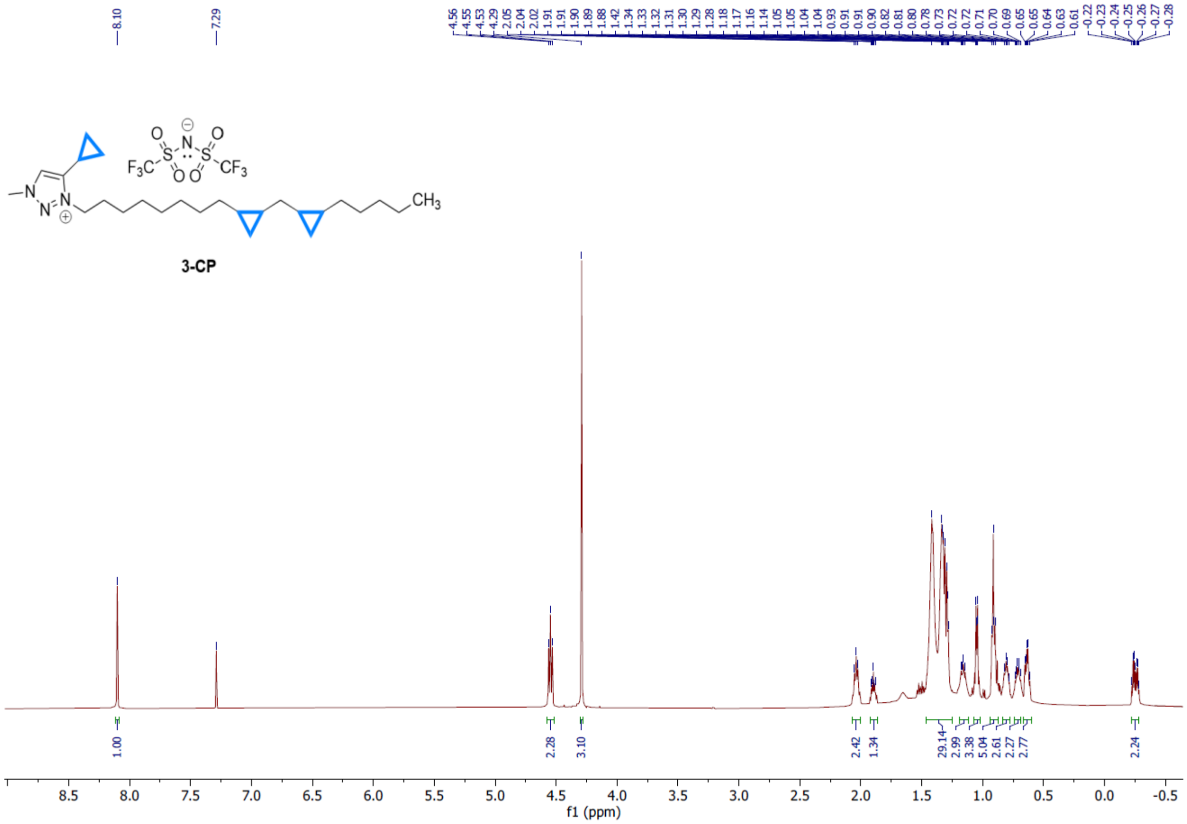

3-CP

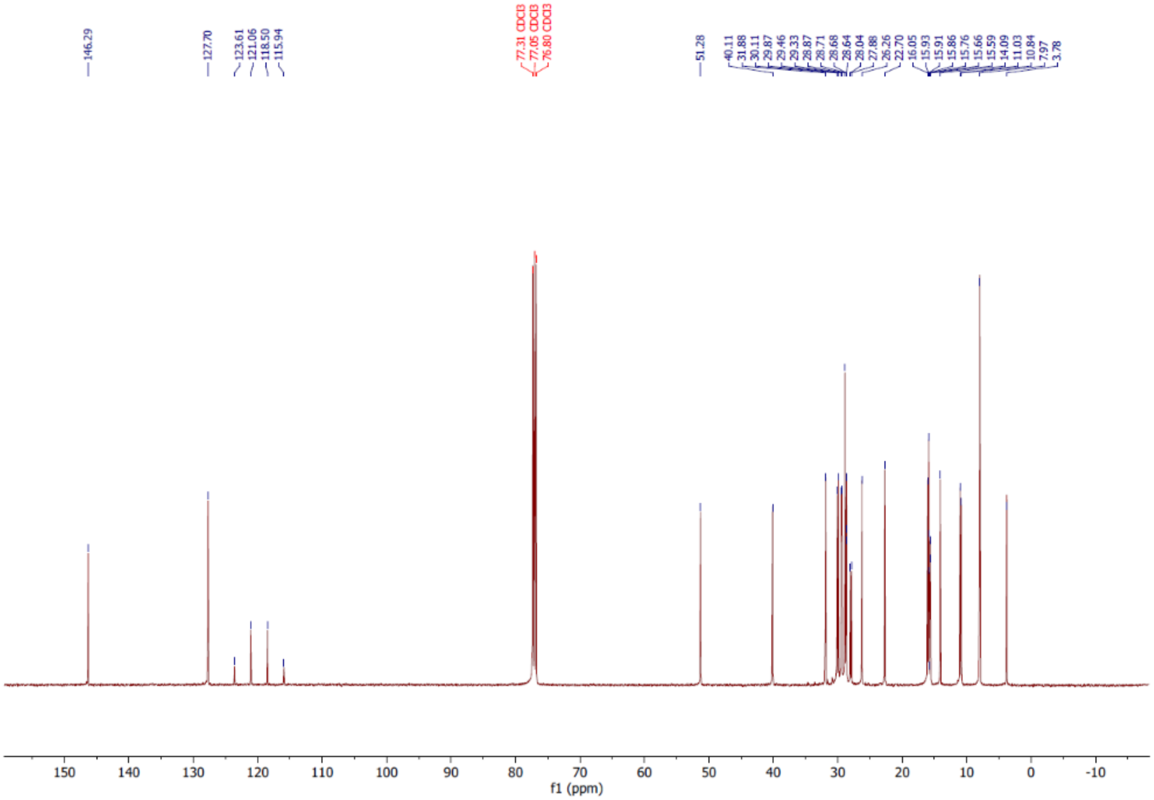

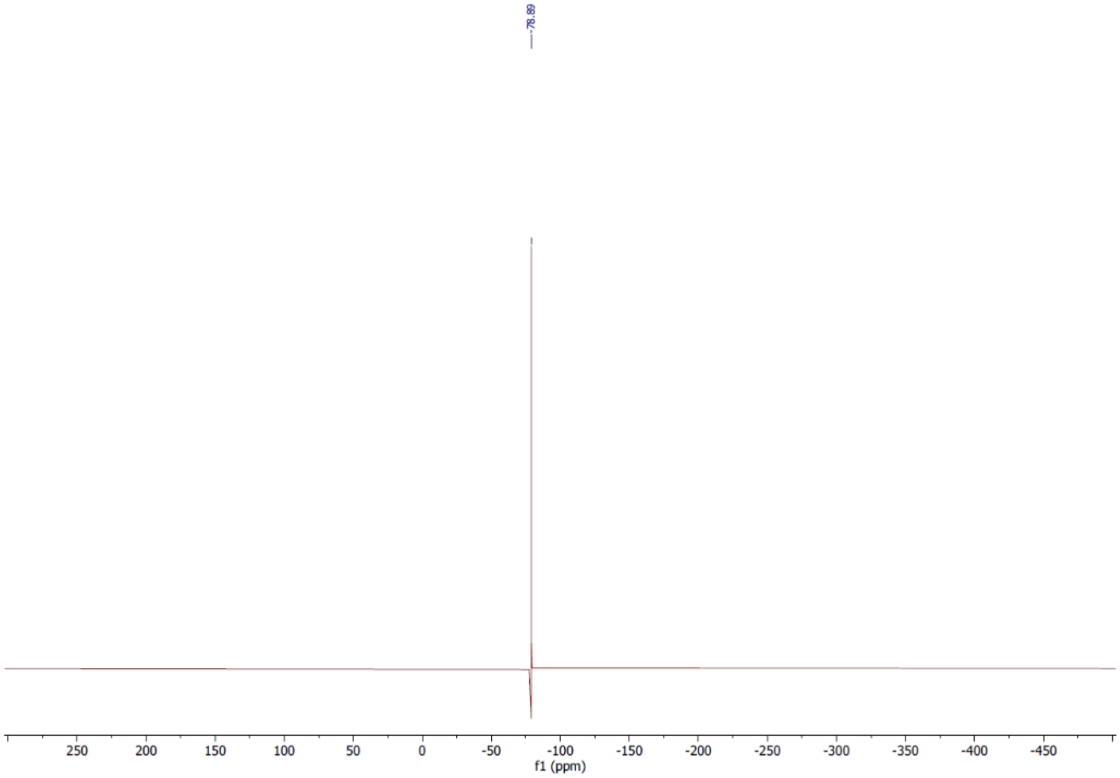

-78.89
250
200
150
100
50
0
-50
-100
-150
-200
-250
-300
-350
-400
-450
f1 (ppm)

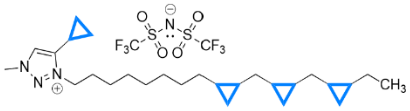

4-CP

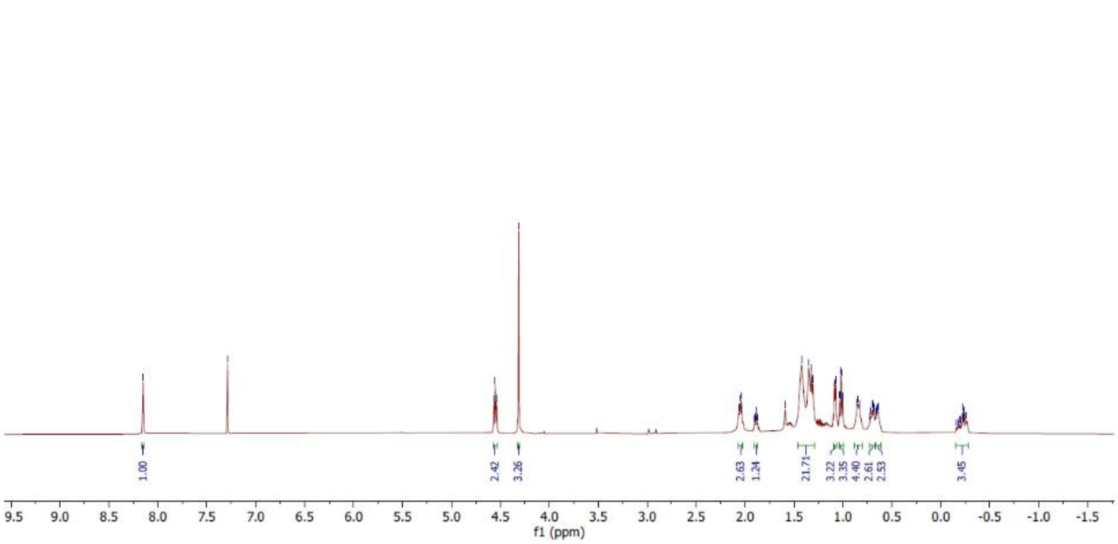

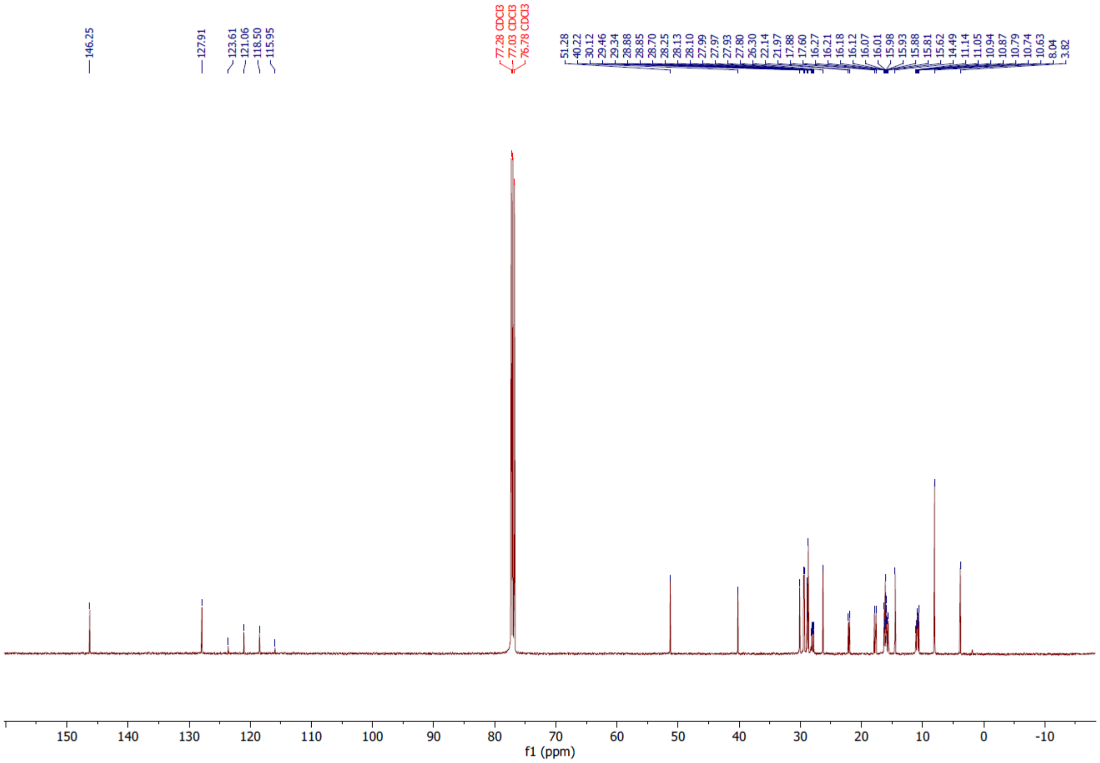

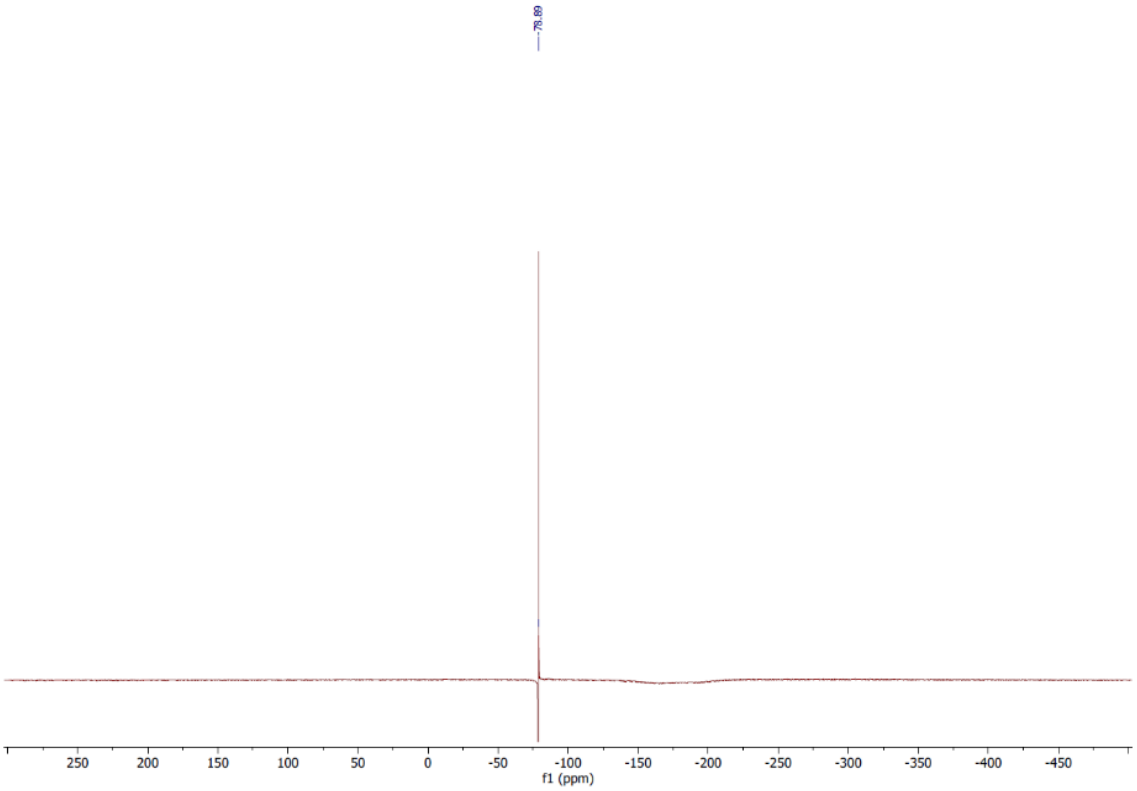

-78.89
250
200
150
100
50
0
-50
-100
-150
-200
-250
-300
-350
-400
-450
f1 (ppm)

DSC and TGA Thermograms of the Products

1 (4-Cyclopropyl-1-methyl-1,2,3-triazole)

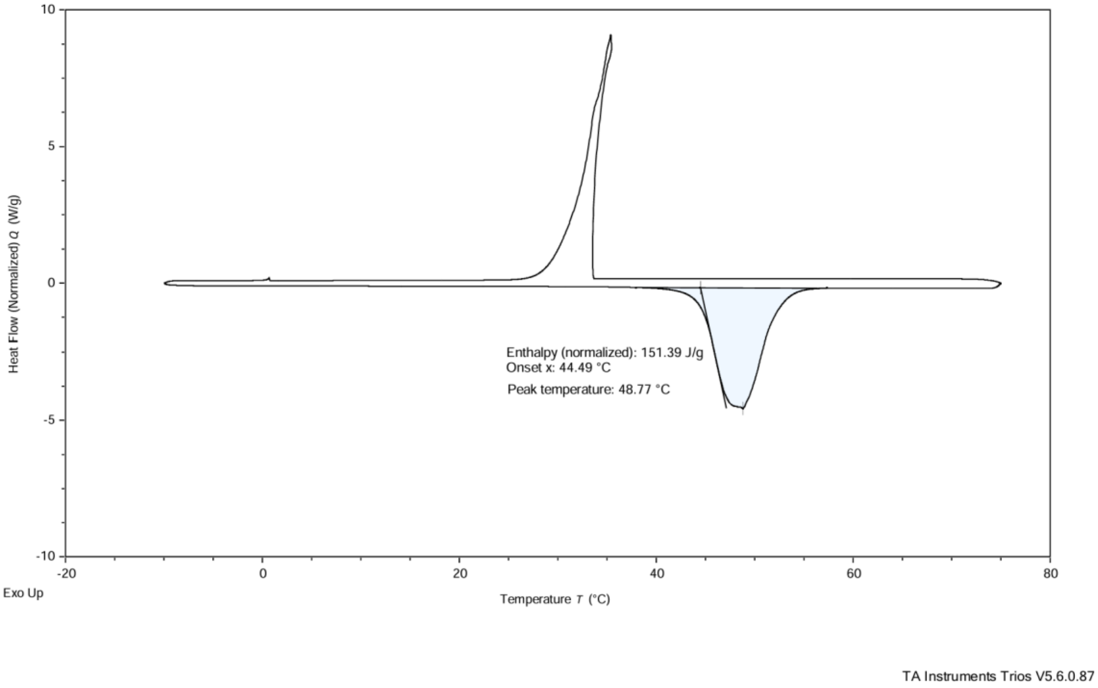

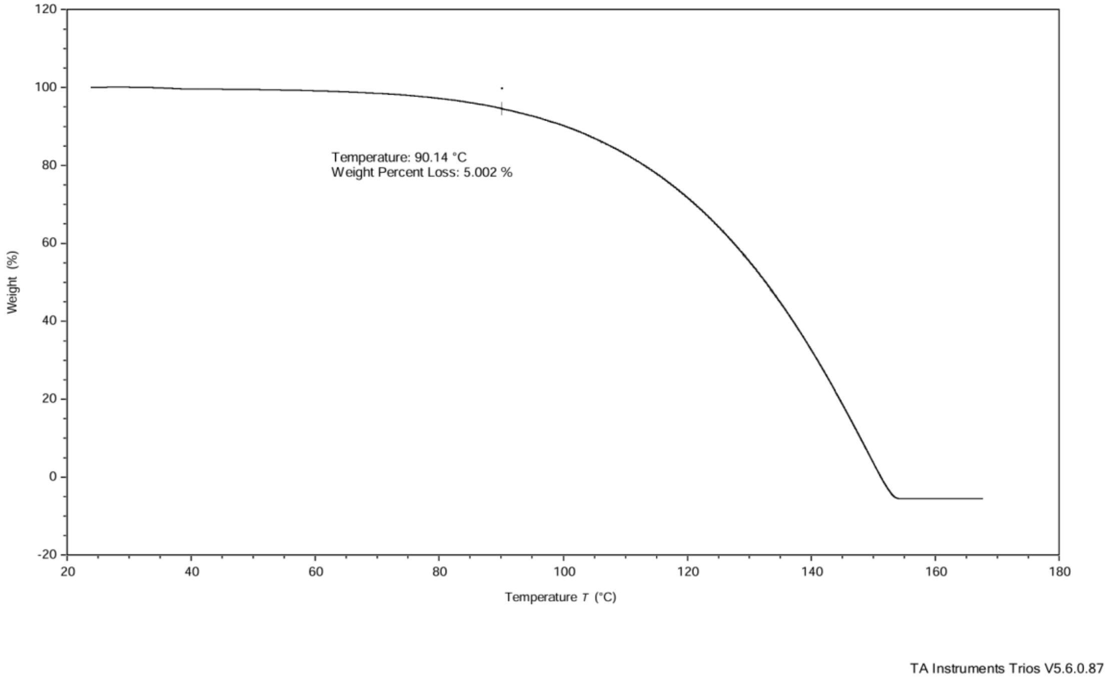

1-AL

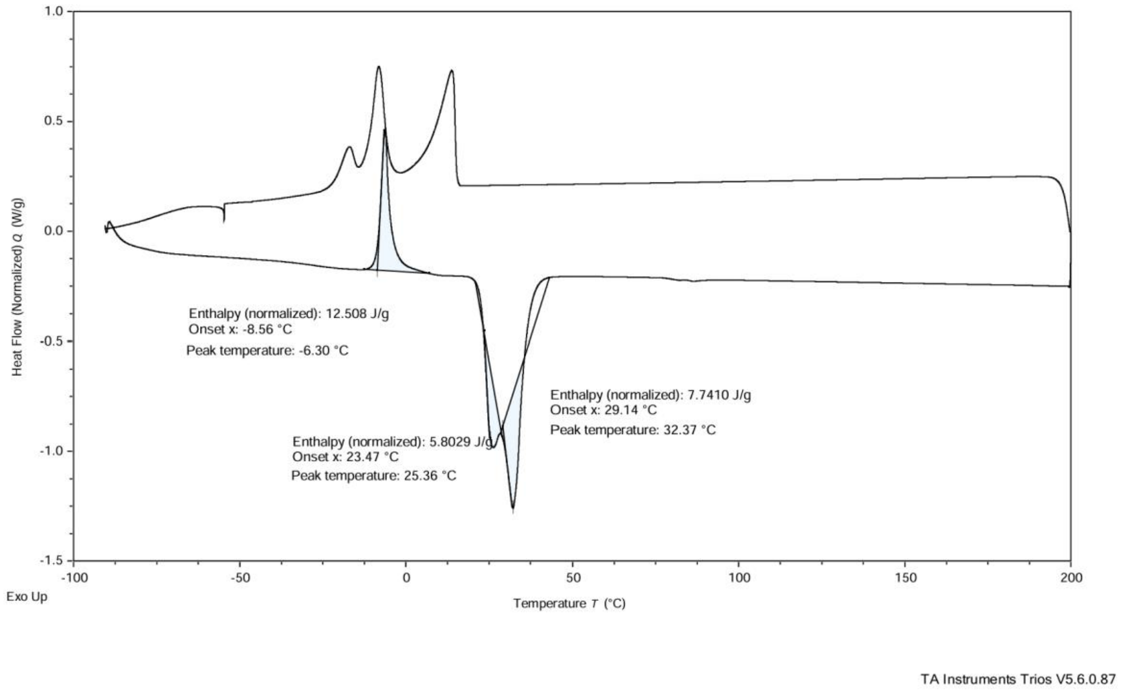
Enthalpy (normalized): 12.508 J/g
Onset x: -8.56 °C
Peak temperature: -6.30 °C
Enthalpy (normalized): 7.7410 J/g
Onset x: 29.14 °C
Peak temperature: 32.37 °C
Enthalpy (normalized): 5.8029 J/g
Onset x: 23.47 °C
Peak temperature: 25.36 °C
Heat Flow (Normalized) Q (W/g)
Temperature T (°C)
Exo Up
TA Instruments Trios V5.6.0.87

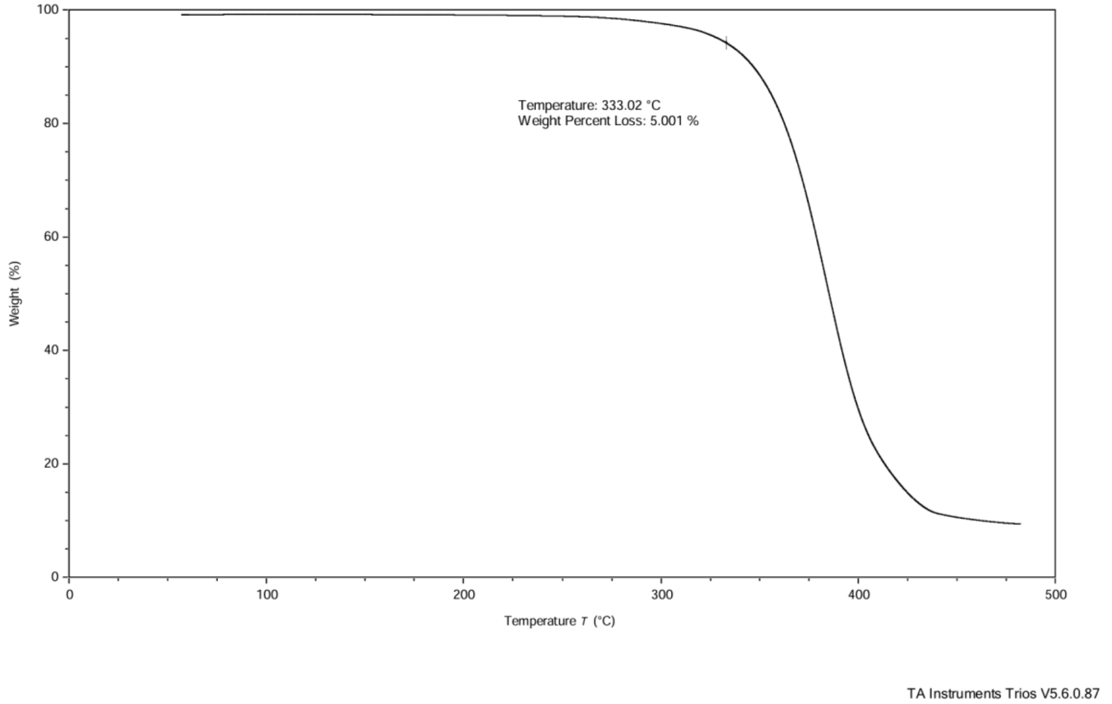
Temperature: 333.02 °C
Weight Percent Loss: 5.001 %
Weight (%)
Temperature T (°C)
TA Instruments Trios V5.6.0.87

2-AL

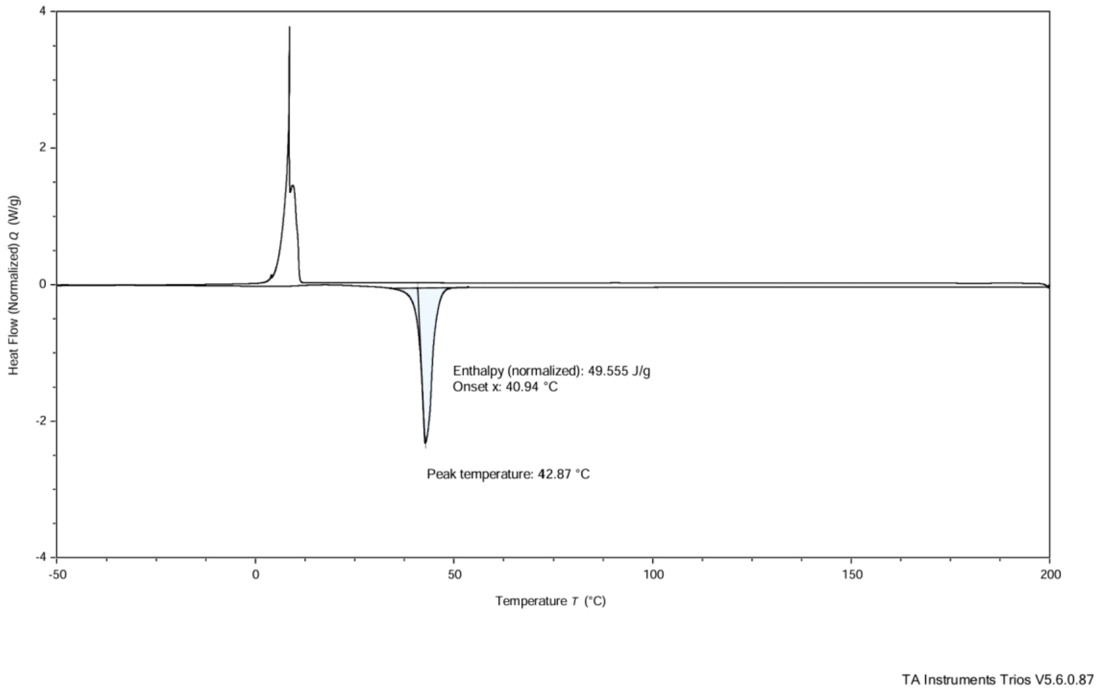
Heat Flow (Normalized) Q (W/g)
Temperature T (°C)
Enthalpy (normalized): 49.555 J/g
Onset x: 40.94 °C
Peak temperature: 42.87 °C
TA Instruments Trios V5.6.0.87

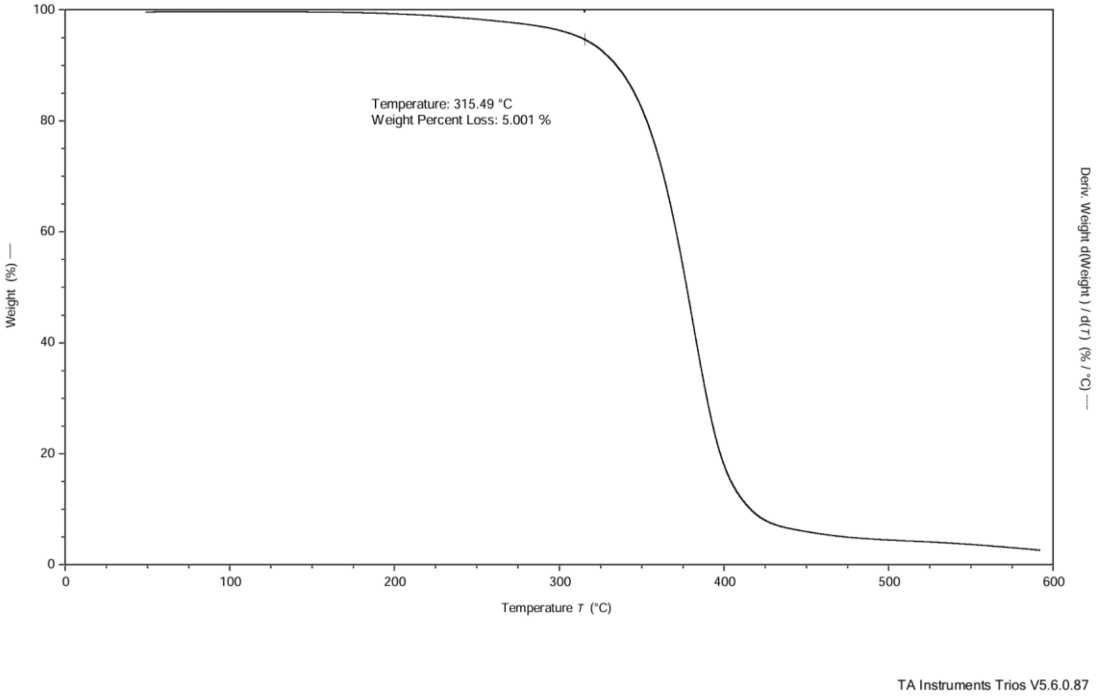
Weight (%)
Deriv. Weight d(Weight) / d(T) (% / °C)
Temperature T (°C)
Temperature: 315.49 °C
Weight Percent Loss: 5.001 %
TA Instruments Trios V5.6.0.87

1-CP

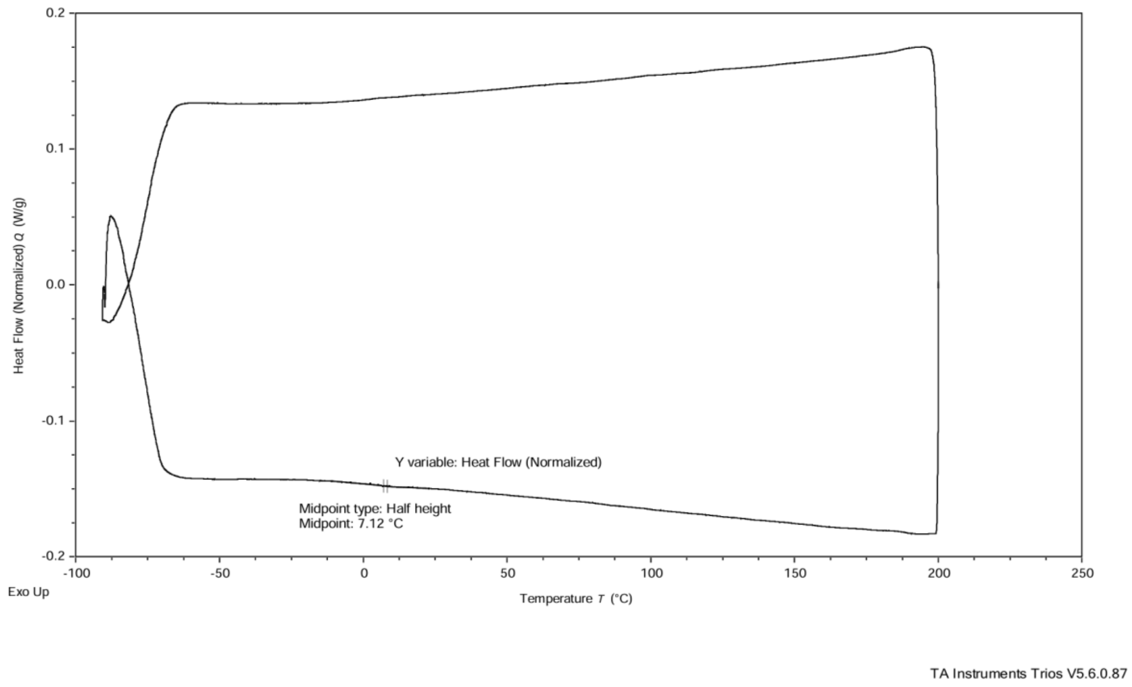
Y variable: Heat Flow (Normalized)
Midpoint type: Half height
Midpoint: 7.12 °C
Heat Flow (Normalized) Q (W/g)
Temperature T (°C)
Exo Up
TA Instruments Trios V5.6.0.87

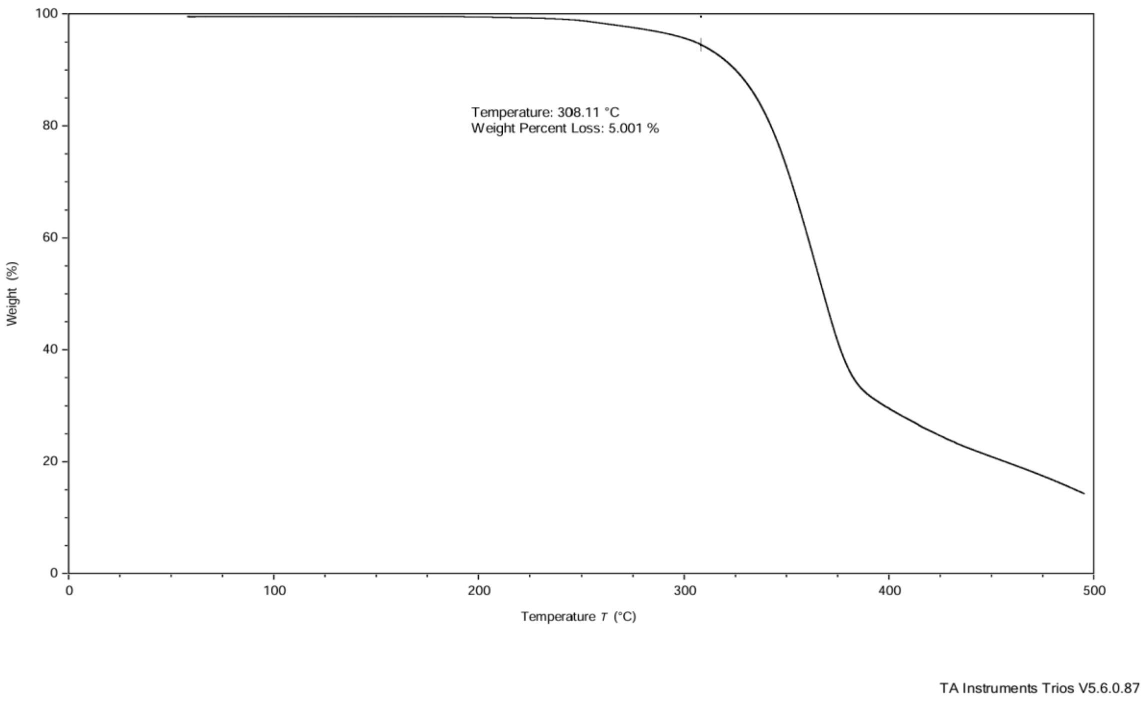
Temperature: 308.11 °C
Weight Percent Loss: 5.001 %
Weight (%)
Temperature T (°C)
TA Instruments Trios V5.6.0.87

2-CP

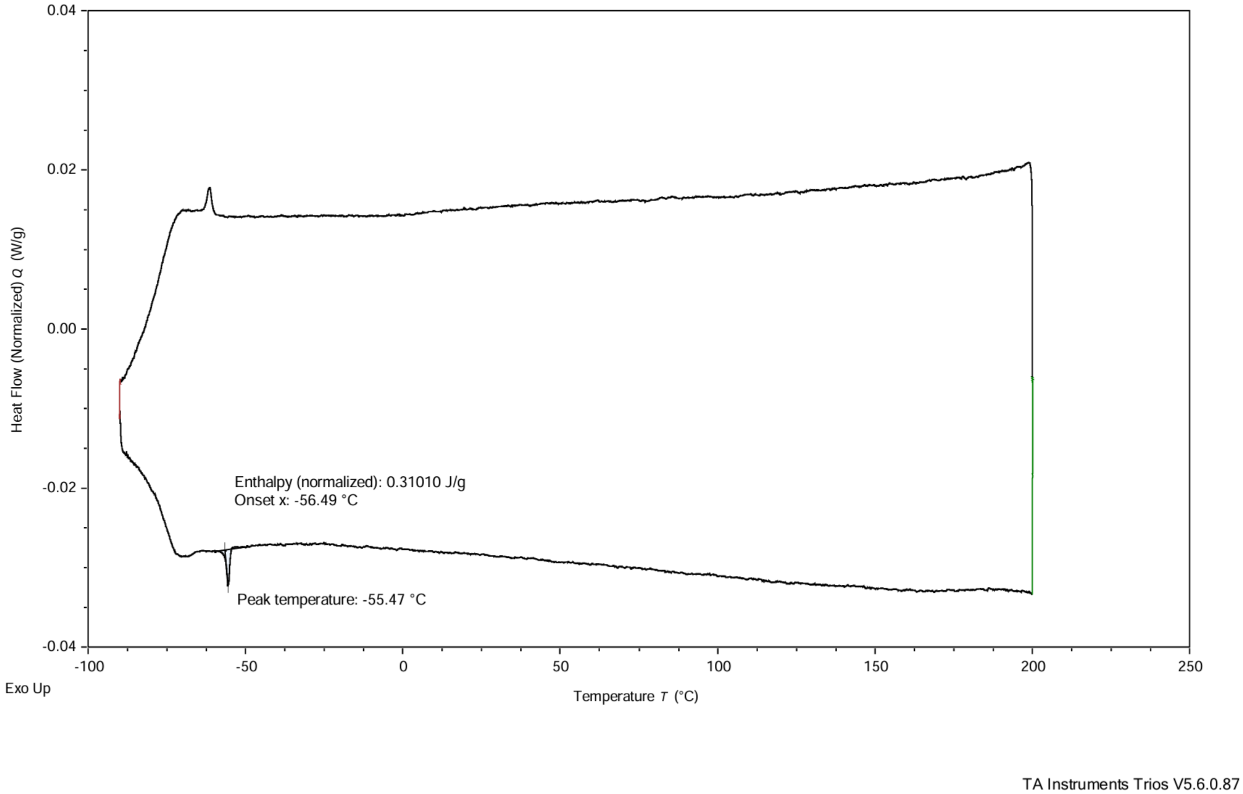

0.04
0.02
0.00
-0.02
-0.04
Heat Flow (Normalized) Q (W/g)
Enthalpy (normalized): 0.31010 J/g
Onset x: -56.49 °C
Peak temperature: -55.47 °C
-100
-50
0
50
100
150
200
250
Exo Up
Temperature T (°C)
TA Instruments Trios V5.6.0.87

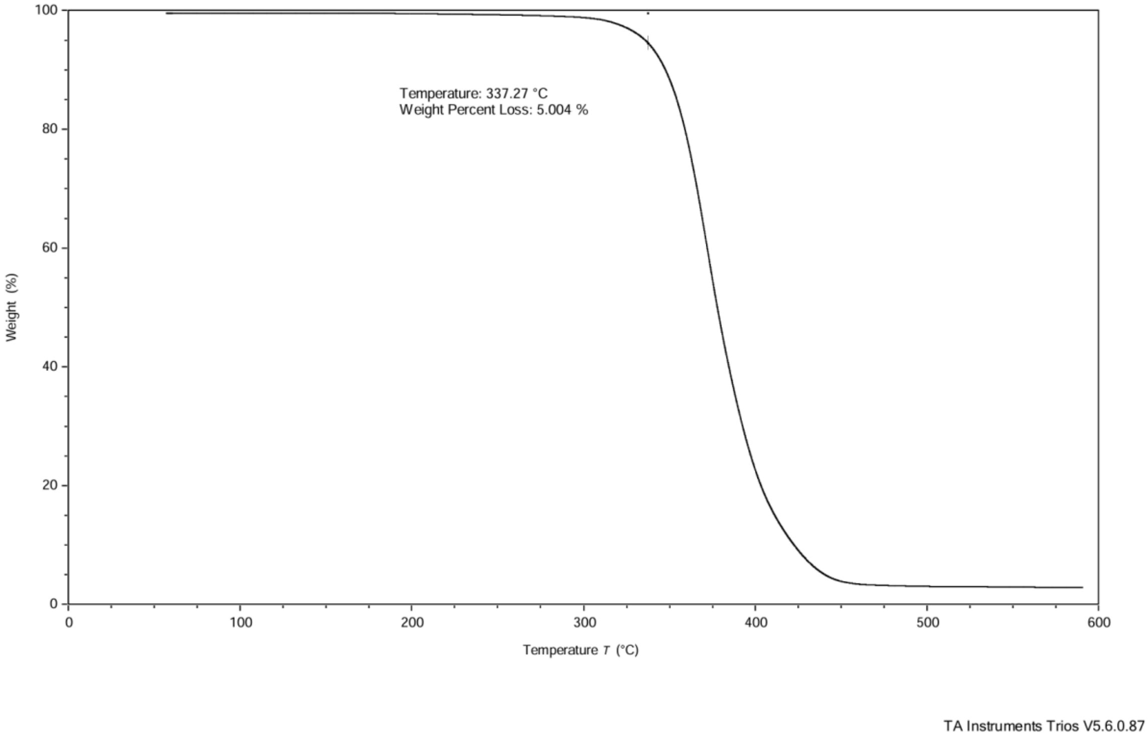

100
80
60
40
20
0
Weight (%)
Temperature: 337.27 °C
Weight Percent Loss: 5.004 %
0
100
200
300
400
500
600
Temperature T (°C)
TA Instruments Trios V5.6.0.87

3-CP

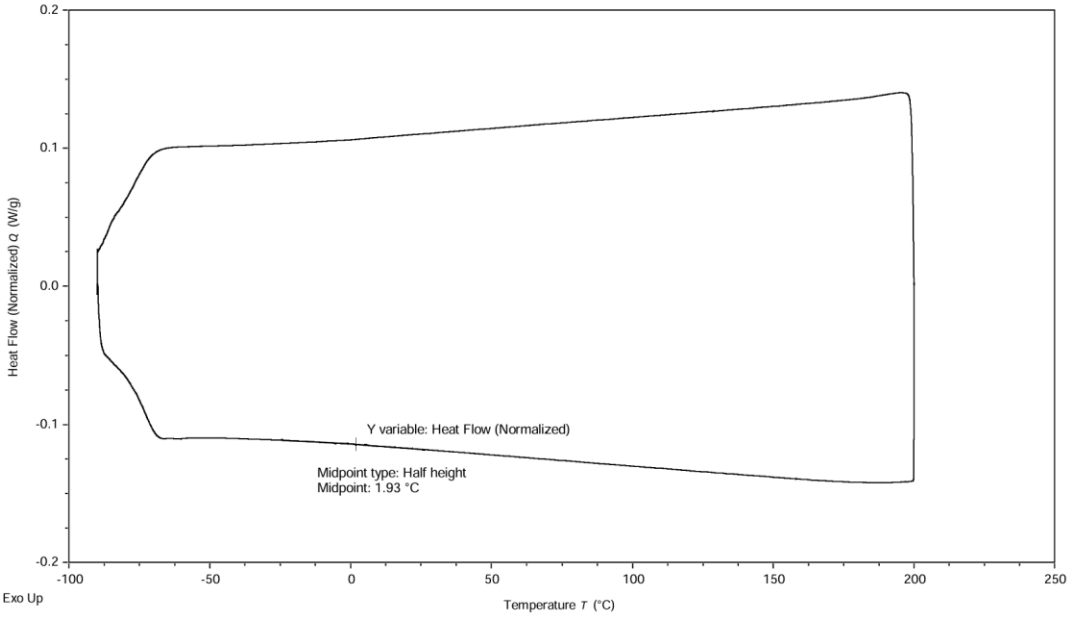

TA Instruments Trios V5.6.0.87

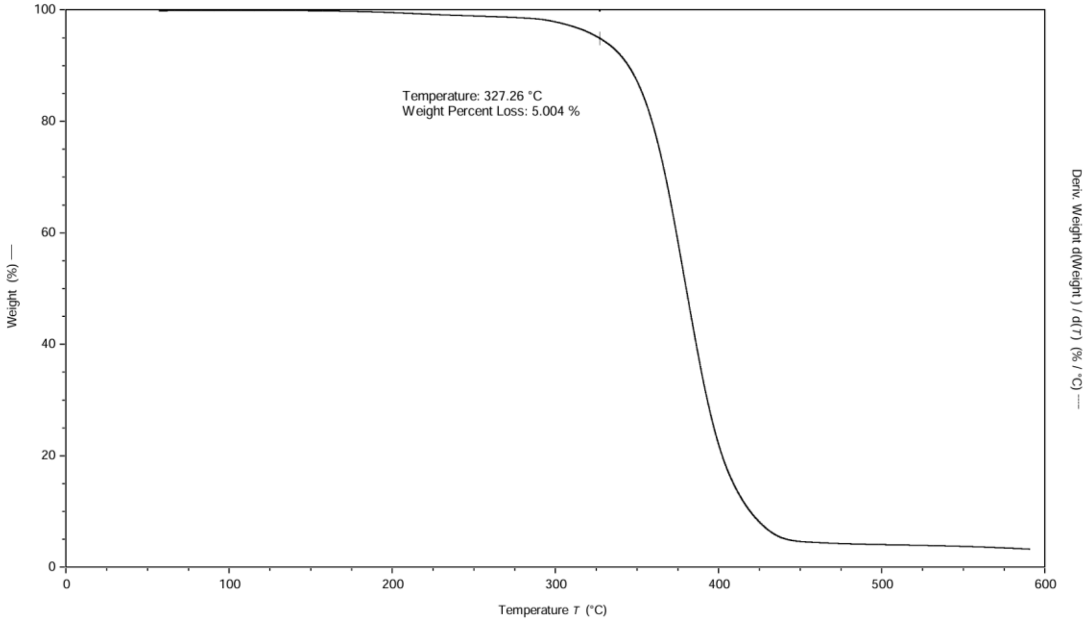

TA Instruments Trios V5.6.0.87

4-CP

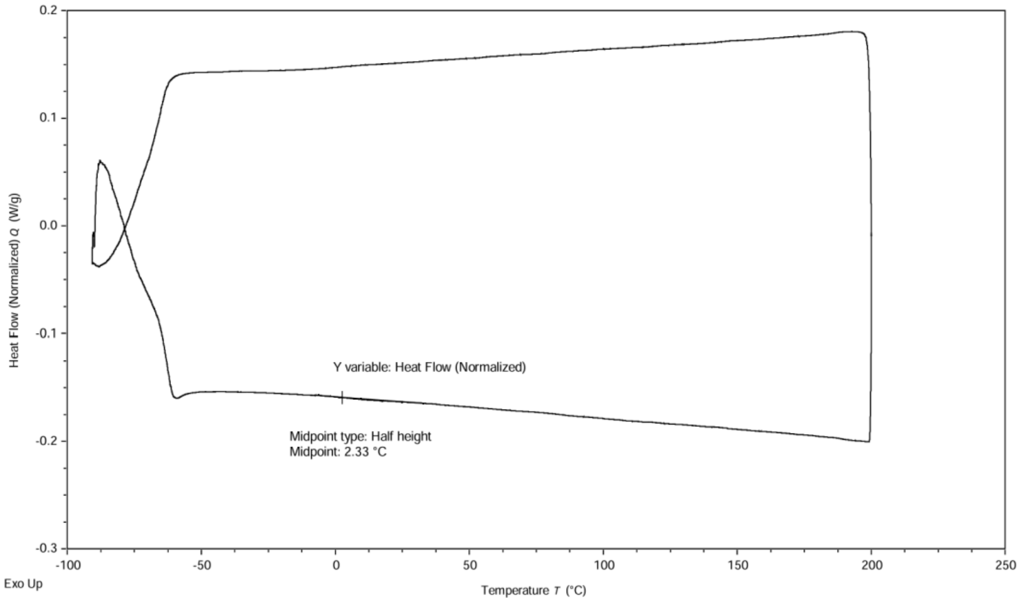

TA Instruments Trios V5.6.0.87

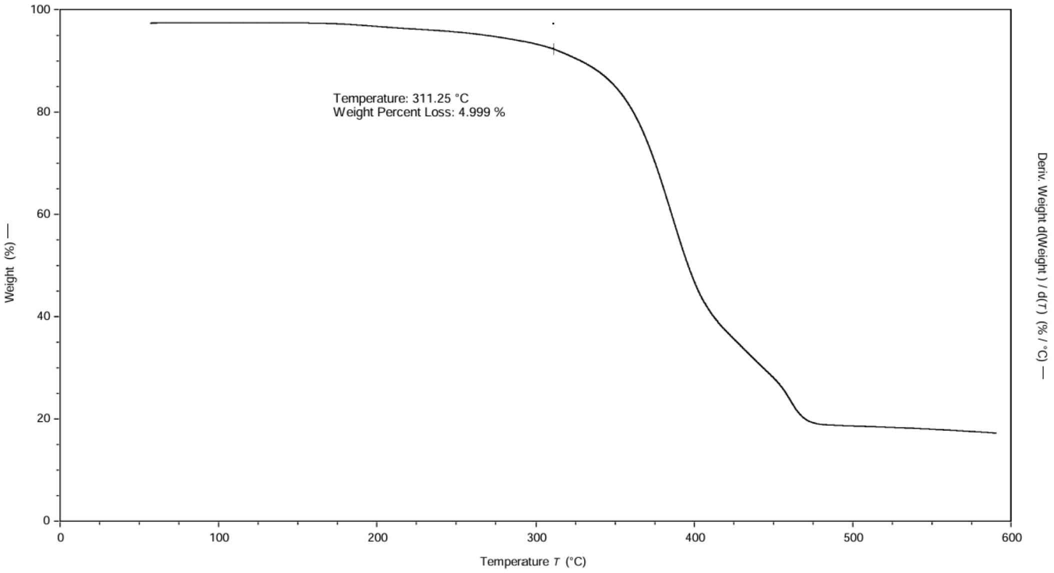

TA Instruments Trios V5.6.0.87

3-AL

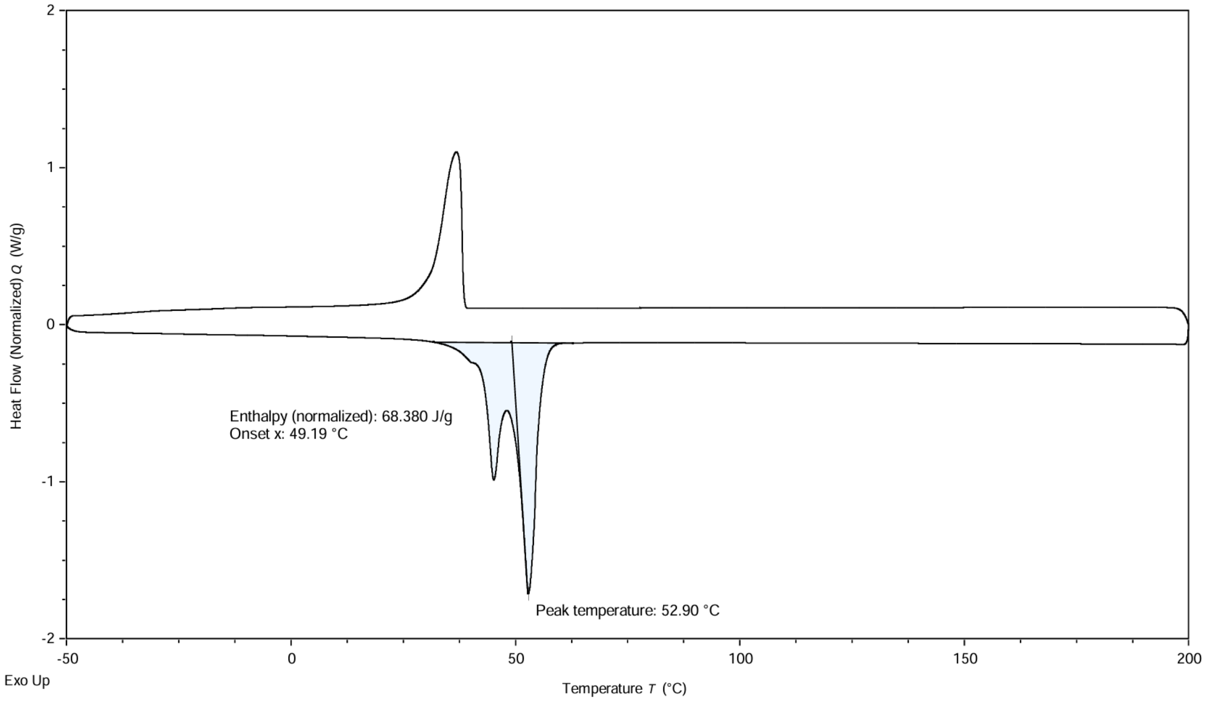

TA Instruments Trios V5.6.0.87

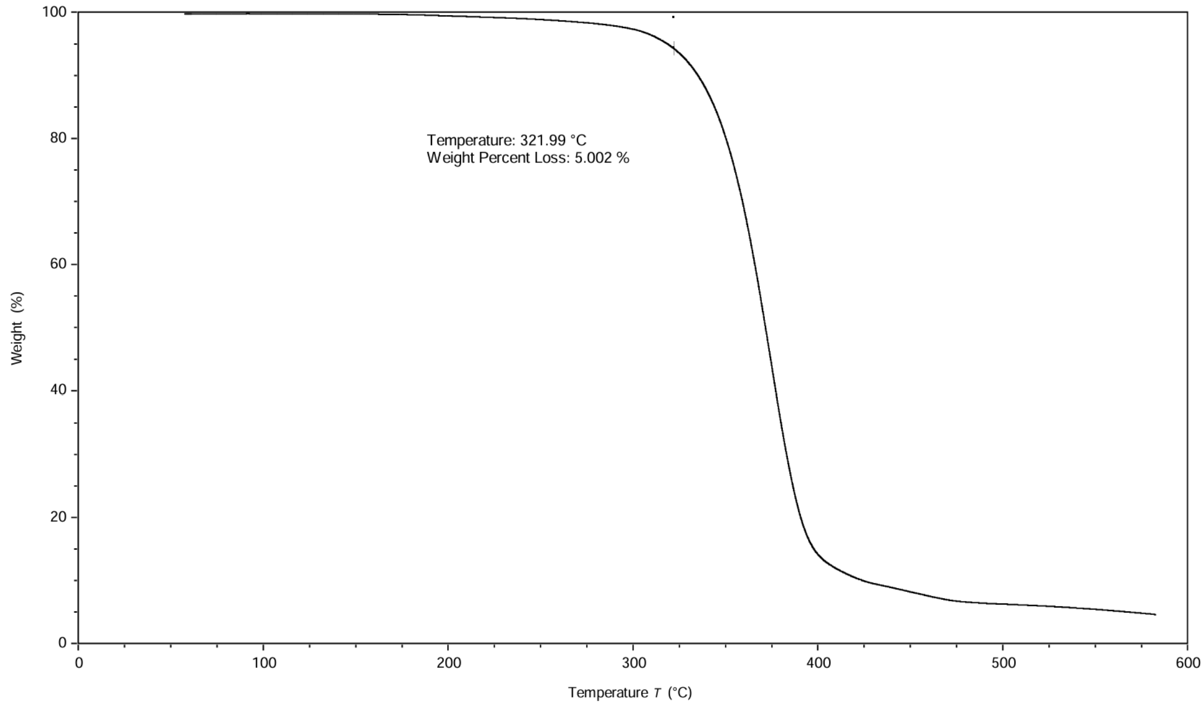

TA Instruments Trios V5.6.0.87

Heat Capacity Thermograms of Products

1-AL

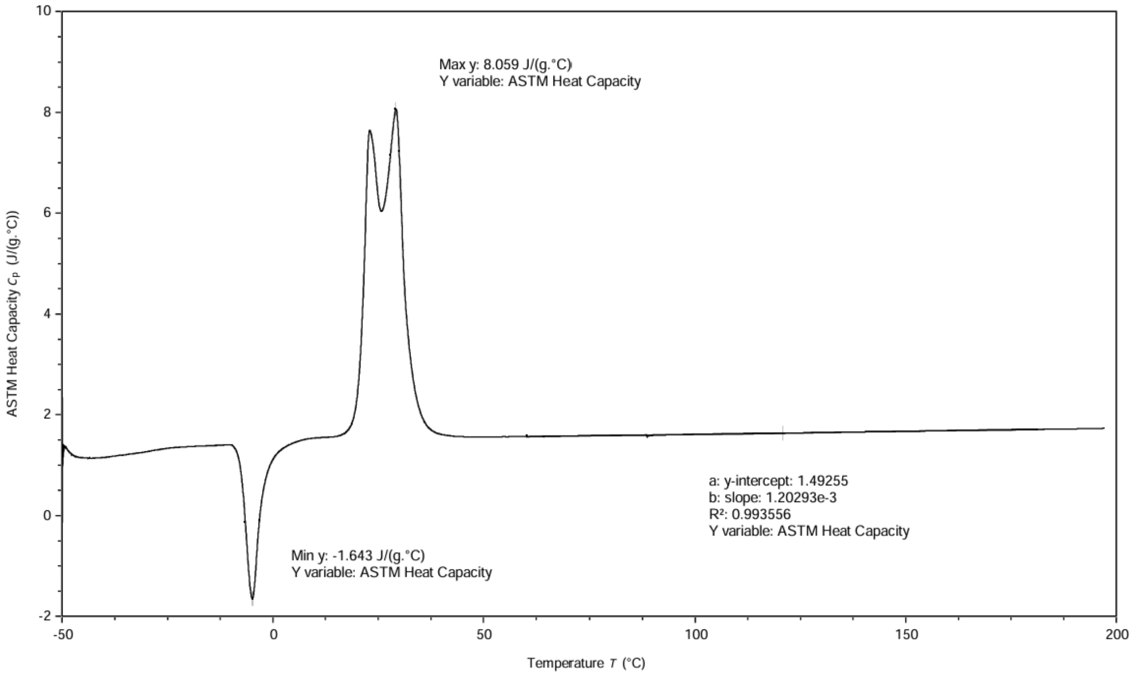

2-AL

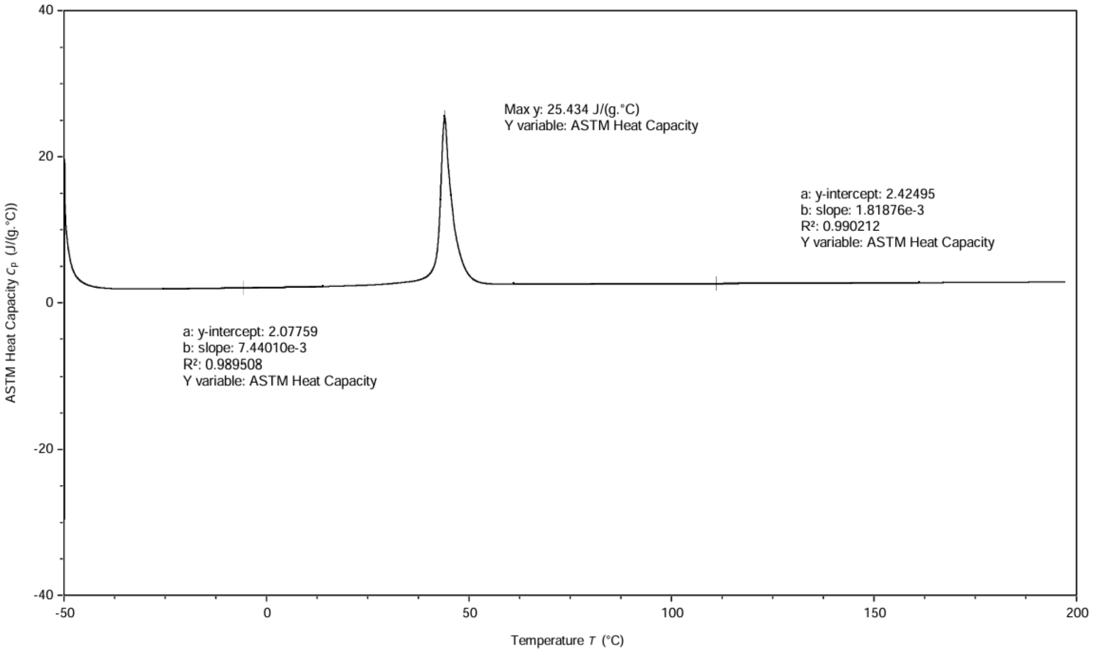

1-CP

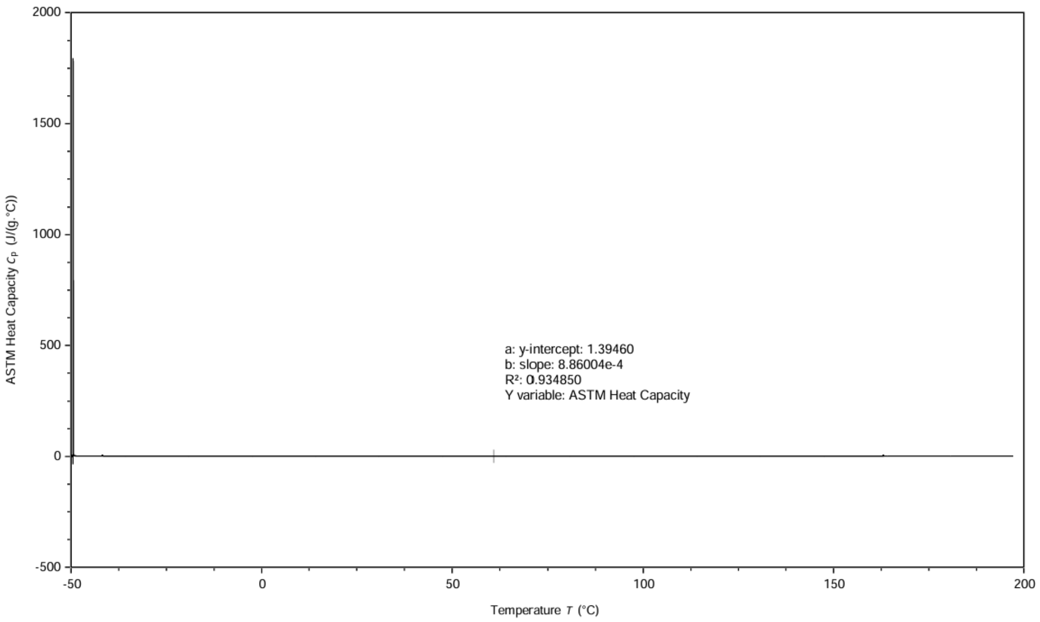
2000
1500
1000
500
0
-500
-50
0
50
100
150
200
ASTM Heat Capacity c_p (J/(g·°C))
Temperature T (°C)
a: y-intercept: 1.39460
b: slope: 8.86004e-4
R²: 0.934850
Y variable: ASTM Heat Capacity

2-CP

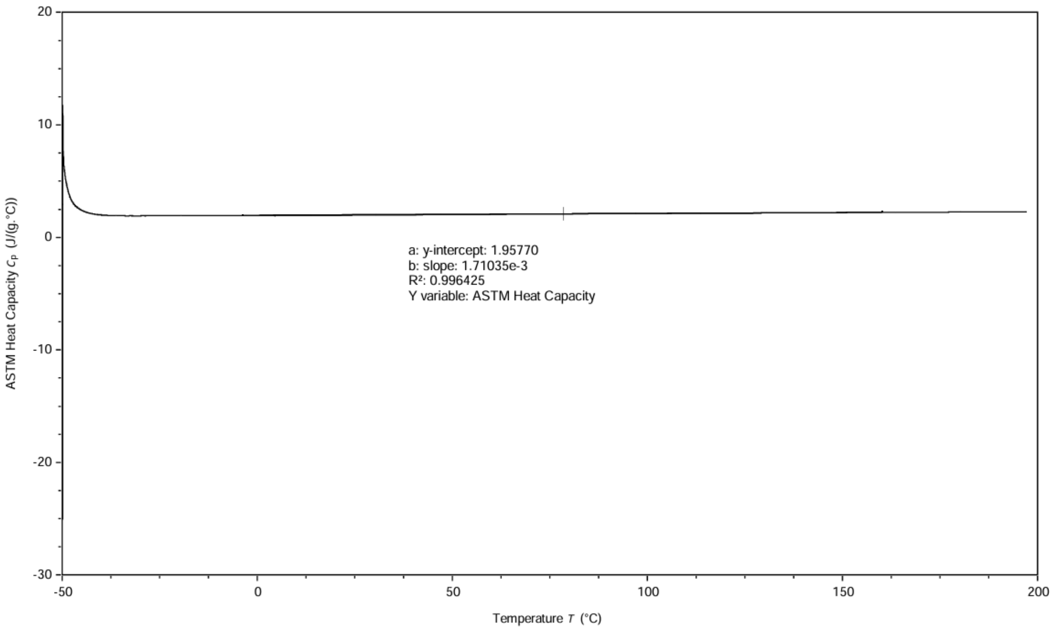
20
10
0
-10
-20
-30
-50
0
50
100
150
200
ASTM Heat Capacity c_p (J/(g·°C))
Temperature T (°C)
a: y-intercept: 1.95770
b: slope: 1.71035e-3
R²: 0.996425
Y variable: ASTM Heat Capacity

3-CP

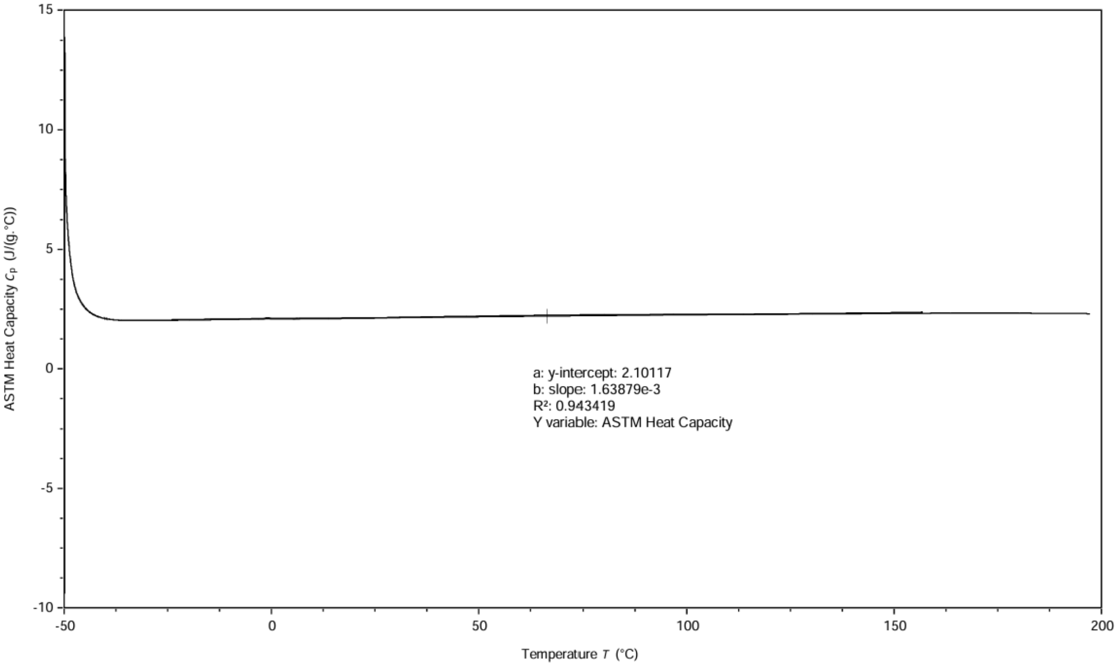

a: y-intercept: 2.10117
b: slope: 1.63879e-3
R²: 0.943419
Y variable: ASTM Heat Capacity
ASTM Heat Capacity c_p (J/(g·°C))
Temperature T (°C)

4-CP

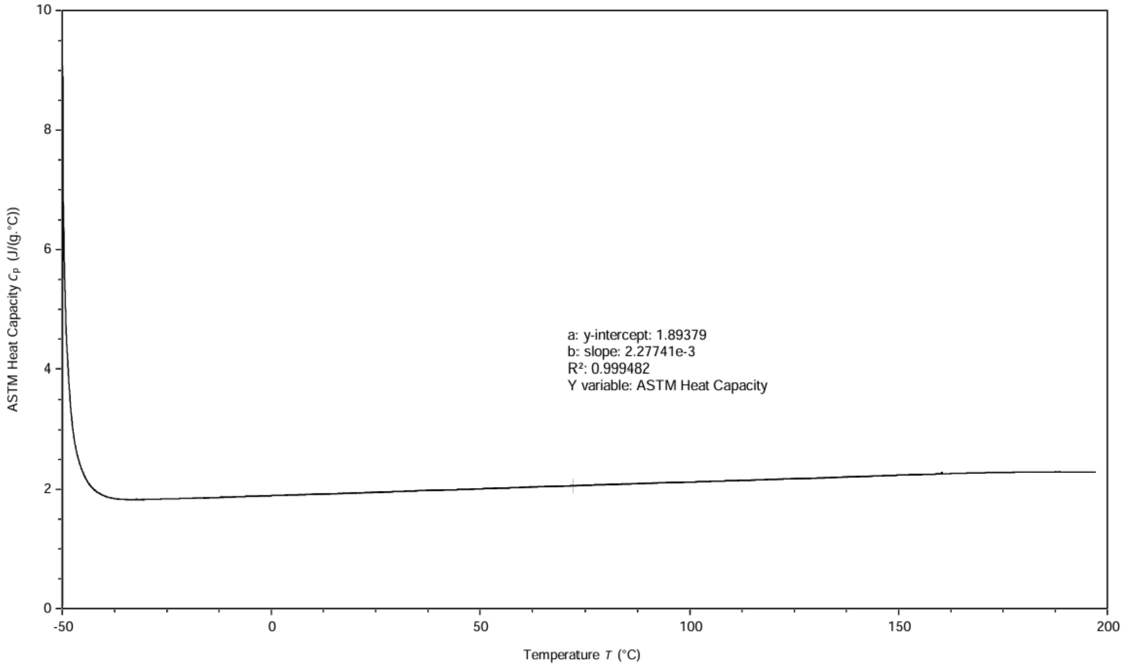

a: y-intercept: 1.89379
b: slope: 2.27741e-3
R²: 0.999482
Y variable: ASTM Heat Capacity
ASTM Heat Capacity c_p (J/(g·°C))
Temperature T (°C)

3-AL

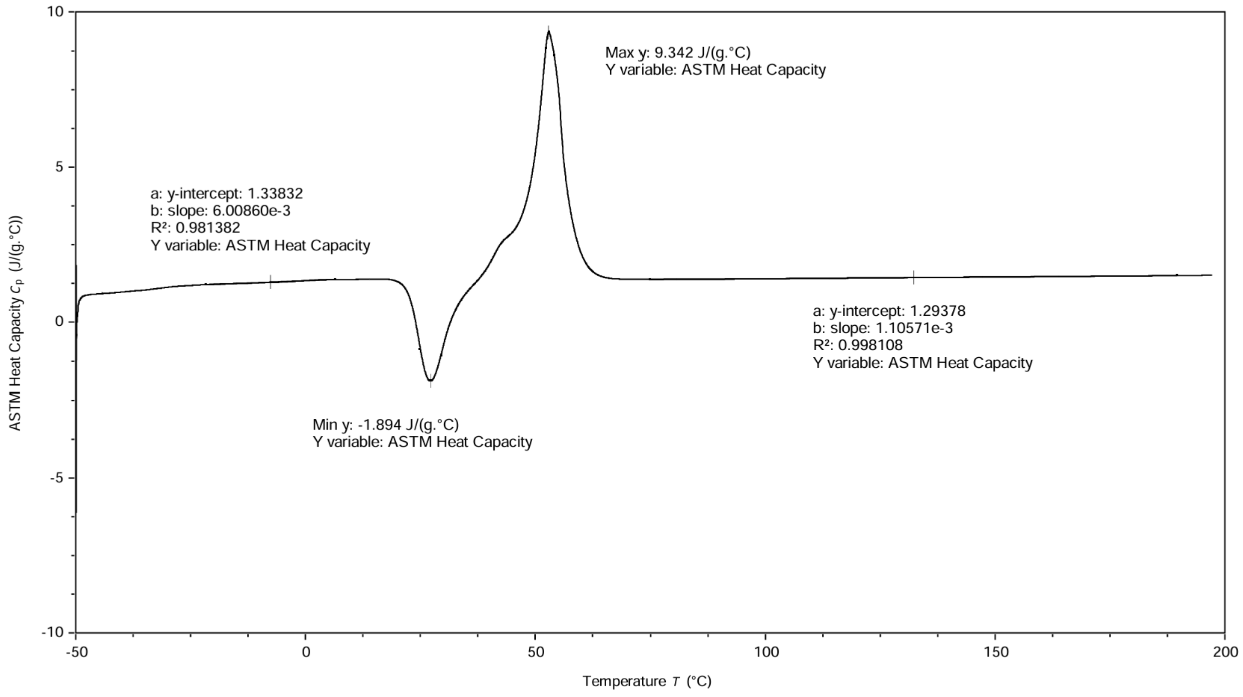

Max y: 9.342 J/(g.°C)
Y variable: ASTM Heat Capacity
a: y-intercept: 1.33832
b: slope: 6.00860e-3
R²: 0.981382
Y variable: ASTM Heat Capacity
a: y-intercept: 1.29378
b: slope: 1.10571e-3
R²: 0.998108
Y variable: ASTM Heat Capacity
Min y: -1.894 J/(g.°C)
Y variable: ASTM Heat Capacity
ASTM Heat Capacity c_p (J/(g.°C))
Temperature T (°C)
