# Supplementary material for: Polycyclopropanated Lipid-Inspired Ionic Liquids as High Energy-Density Fuel Candidates
Source: ACS Sustain Chem Eng. 2026 Feb 23;14(9):4596–607. doi: 10.1021/acssuschemeng.5c13132 (PMC12977155; doi:10.1021/acssuschemeng.5c13132)
Supplement: Supplementary file 2 [file sc5c13132_si_002.zip › Compound 1.pdf]

checkCIF (basic structural check) running

Checking for embedded fcf data in CIF ...
Found embedded fcf data in CIF. Extracting fcf data from uploaded CIF, please wait . .

checkCIF/PLATON (basic structural check)

Structure factors have been supplied for datablock(s) Butch_C_CB1_020124_0m

THIS REPORT IS FOR GUIDANCE ONLY. IF USED AS PART OF A REVIEW PROCEDURE FOR PUBLICATION, IT SHOULD NOT REPLACE THE EXPERTISE OF AN EXPERIENCED CRYSTALLOGRAPHIC REFEREE.

No syntax errors found. CIF dictionary
Please wait while processing .... Interpreting this report

Structure factor report

Datablock: Butch_C_CB1_020124_0m

Bond precision: C-C = 0.0014 A Wavelength=0.71073

Cell:	a=10.6759(6)	b=5.5233(3)	c=11.1634(7)
	alpha=90	beta=92.689(3)	gamma=90
Temperature:	150 K		

	Calculated	Reported
Volume	657.54(7)	657.54(7)
Space group	P 21/c	P 21/c
Hall group	-P 2ybc	-P 2ybc
Moiety formula	C6 H9 N3	?
Sum formula	C6 H9 N3	C6 H9 N3
Mr	123.16	123.16
Dx,g cm-3	1.244	1.244
Z	4	4
Mu (mm-1)	0.081	0.081
F000	264.0	264.0
F000'	264.07	
h,k,lmax	16,8,17	16,8,17
Nref	2520	2520
Tmin,Tmax	0.987,0.991	0.656,0.747
Tmin'	0.956	

Correction method= # Reported T Limits: Tmin=0.656 Tmax=0.747 AbsCorr = MULTI-SCAN

Data completeness= 1.000 Theta(max)= 33.165

R(reflections)= 0.0436(1883) wR2(reflections)= 0.1253(2520)

S = 1.063 Npar= 83

The following ALERTS were generated. Each ALERT has the format
test-name_ALERT_alert-type_alert-level.
Click on the hyperlinks for more details of the test.

Alert level G

PLAT910_ALERT_3_G Missing # of FCF Reflection(s) Below Theta(Min). 1 Note
1 0 0,
PLAT933_ALERT_2_G Number of HKL-OMIT Records in Embedded .res File 1 Note
1 0 0,
PLAT969_ALERT_5_G The 'Henn et al.' R-Factor-gap value .... 3.73 Note
Predicted wR2: Based on SigI**2 3.36 or SHELX Weight 11.98

PLAT978_ALERT_2_G Number C-C Bonds with Positive Residual Density. 5 Info

0 **ALERT level A** = Most likely a serious problem - resolve or explain
0 **ALERT level B** = A potentially serious problem, consider carefully
0 **ALERT level C** = Check. Ensure it is not caused by an omission or oversight
4 **ALERT level G** = General information/check it is not something unexpected

0 ALERT type 1 CIF construction/syntax error, inconsistent or missing data
2 ALERT type 2 Indicator that the structure model may be wrong or deficient
1 ALERT type 3 Indicator that the structure quality may be low
0 ALERT type 4 Improvement, methodology, query or suggestion
1 ALERT type 5 Informative message, check

It is advisable to attempt to resolve as many as possible of the alerts in all categories. Often the minor alerts point to easily fixed oversights, errors and omissions in your CIF or refinement strategy, so attention to these fine details can be worthwhile. In order to resolve some of the more serious problems it may be necessary to carry out additional measurements or structure refinements. However, the purpose of your study may justify the reported deviations and the more serious of these should normally be commented upon in the discussion or experimental section of a paper or in the "special_details" fields of the CIF. checkCIF was carefully designed to identify outliers and unusual parameters, but every test has its limitations and alerts that are not important in a particular case may appear. Conversely, the absence of alerts does not guarantee there are no aspects of the results needing attention. It is up to the individual to critically assess their own results and, if necessary, seek expert advice.

Publication of your CIF in IUCr journals

A basic structural check has been run on your CIF. These basic checks will be run on all CIFs submitted for publication in IUCr journals (*Acta Crystallographica*, *Journal of Applied Crystallography*, *Journal of Synchrotron Radiation*); however, if you intend to submit to *Acta Crystallographica Section C* or *E* or *IUCrData*, you should make sure that full publication checks are run on the final version of your CIF prior to submission.

Publication of your CIF in other journals

Please refer to the *Notes for Authors* of the relevant journal for any special instructions relating to CIF submission.

PLATON version of 06/01/2024; check.def file version of 05/01/2024

Datablock Butch_C_CB1_020124_0m - ellipsoid plot

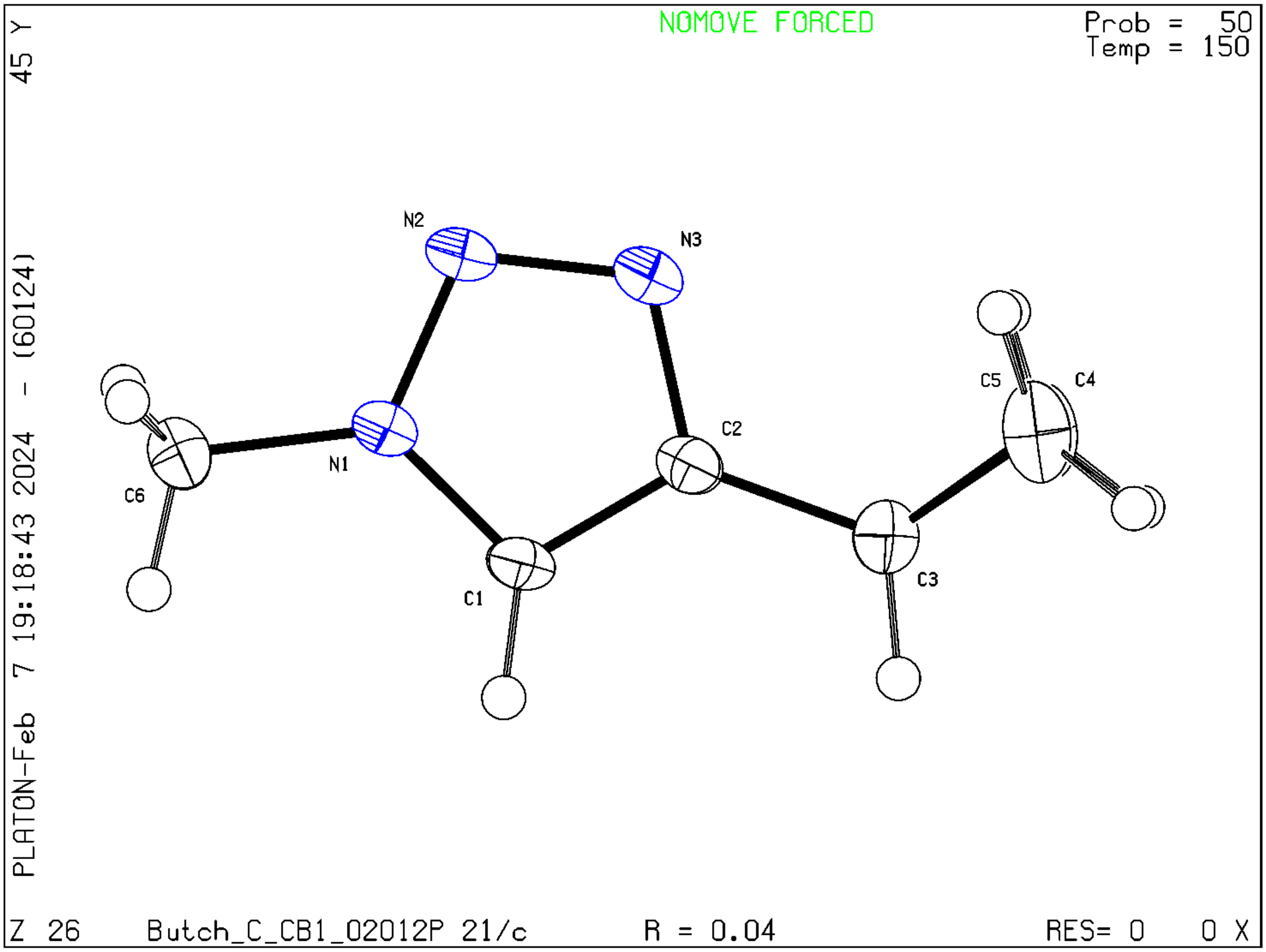

Download CIF editor (publCIF) from the IUCr
Download CIF editor (enCIFer) from the CCDC
Test a new CIF entry
